# *Ezh2* does not mediate retinal ganglion cell homeostasis or their susceptibility to injury

**DOI:** 10.1371/journal.pone.0191853

**Published:** 2018-02-06

**Authors:** Lin Cheng, Lucy J. Wong, Naihong Yan, Richard C. Han, Honghua Yu, Chenying Guo, Khulan Batsuuri, Aniket Zinzuwadia, Ryan Guan, Kin-Sang Cho, Dong Feng Chen

**Affiliations:** 1 State Key Laboratory of Ophthalmology, Zhongshan Ophthalmic Center, Sun Yat-sen University, Guangzhou, Guangdong, P. R. China; 2 Schepens Eye Research Institute, Massachusetts Eye & Ear, Department of Ophthalmology, Harvard Medical School, Boston, Massachusetts, United States of America; 3 Eye Center, Medical Center, Faculty of Medicine, University of Freiburg, Freiburg, Germany; 4 Department of Ophthalmology and Ophthalmic Laboratories, State Key Laboratory of Biotherapy and Cancer Center, West China Hospital, Sichuan University, Chengdu, Sichuan, P. R. China; National Eye Centre, UNITED STATES

## Abstract

Epigenetic predisposition is thought to critically contribute to adult-onset disorders, such as retinal neurodegeneration. The histone methyltransferase, enhancer of zeste homolog 2 (*Ezh2*), is transiently expressed in the perinatal retina, particularly enriched in retinal ganglion cells (RGCs). We previously showed that embryonic deletion of *Ezh2* from retinal progenitors led to progressive photoreceptor degeneration throughout life, demonstrating a role for embryonic predisposition of *Ezh2-*mediated repressive mark in maintaining the survival and function of photoreceptors in the adult. Enrichment of *Ezh2* in RGCs leads to the question if *Ezh2* also mediates gene expression and function in postnatal RGCs, and if its deficiency changes RGC susceptibility to cell death under injury or disease in the adult. To test this, we generated mice carrying targeted deletion of *Ezh2* from RGC progenitors driven by *Math5-Cre* (mKO). mKO mice showed no detectable defect in RGC development, survival, or cell homeostasis as determined by physiological analysis, live imaging, histology, and immunohistochemistry. Moreover, RGCs of *Ezh2* deficient mice revealed similar susceptibility against glaucomatous and acute optic nerve trauma-induced neurodegeneration compared to littermate floxed or wild-type control mice. In agreement with the above findings, analysis of RNA sequencing of RGCs purified from *Ezh2* deficient mice revealed few gene changes that were related to RGC development, survival and function. These results, together with our previous report, support a cell lineage-specific mechanism of *Ezh2-*mediated gene repression, especially those critically involved in cellular function and homeostasis.

## Introduction

Epigenetic predisposition in the embryo is reported to regulate postnatal cell homeostasis and gene expression [1]; its disruption contributes critically to the progression of neurodegenerative disorders in adults [2]. Emerging evidence suggests that gene loci linked with retinal diseases, such as age-related macular degeneration and glaucoma, are also mediated by an epigenetic mechanism [3, 4]. Changes in histone modifications have been associated with the incidence and/or progression of optic nerve injury-induced neuron loss [5, 6]. Dysregulation of epigenetic modifications during development led to chronic and progressive photoreceptor death throughout the postnatal life [7, 8]. These observations suggest a role for histone modifiers in stress response and tissue vulnerability.

Accumulating data implicate that histone modification is regulated in a cell type-specific manner [9]. An intensively studied histone modifier is *Ezh2* (Enhancer of Zeste homlog 2), a major histone methyltransferase (HMTase). It catalyzes the tri-methylation of histone H3 at lysine 27 (*H3K27me3*) to establish a repressive chromatin structure [10]. *Ezh2* has been extensively investigated for its roles in stem cell pluripotency, neural development, tumorigenesis, and inflammation [11–13]. It is reported that *Ezh2*-mediated histone methylation also influences tissue homeostasis, including in the retina [8], by silencing developmental genes to allow completion and stabilization of cell maturation [11, 14, 15]. Mice carrying retinal deficiency of *Ezh2* develop either retinal progenitor cell defects or selective degeneration of photoreceptors in the postnatal life [8, 16, 17], suggesting its regulation in both stem cell differentiation and retinal homeostasis. Although the mRNA or protein of *Ezh2* is not detected in the adult mouse retina, its expression is found in the embryonic eye, with a particularly high level in perinatal post-mitotic RGCs [18]. To date, the involvement of *Ezh2* in RGC development or homeostasis remains unreported.

The highly enriched expression of *Ezh2* in perinatal RGCs raises the question if *Ezh2* plays a key role in mediating gene expression and maturation in postnatal RGCs and if its dysregulation in the embryonic stage contributes to RGC degeneration under the disease or injury in the adult. Adult-onset RGC degenerative diseases, such as glaucoma, are a leading cause of blindness worldwide [19, 20]. Although elevated intraocular pressure (IOP) is recognized as a major risk factor, progressive RGC degeneration occurs only in some, but not all people with elevated IOP, and even in many patients who have a normal IOP. Thus, the mechanisms underlying glaucomatous neuron loss remain unknown. We thus asked if dysregulation of epigenetic markers, such as those mediated by *Ezh2*, contribute to this process. To test this, we generated mice carrying *Ezh2* inactivation driven by *Math5*-*Cre* [21]. *Math5* is a basic helix-loop-helix (bHLH) proneural gene that is essential for RGC development [22, 23]. *Math5* activates a comprehensive transcription network for RGC differentiation [21]. While absence of *Math5* induces selective loss of RGCs in the mouse retina [24], only ~11% of *Math5*-expressing cells adapt an RGC fate [25], and the rest of the *Math5*+ retinal progenitors differentiate into other retinal cell types and contribute to a small proportion (<10%) of non-RGC lineages, such as photoreceptors and amacrine cells [21, 26, 27]. It is reported that ~55% of adult RGCs in *Math5*-Cre transgenic reporter mice were positive for Cre expression and that *Math5-Cre* drives targeted gene deletion in approximately half of the RGC population [27]. Mice carrying targeted deletion of *Ezh2* driven by *Math5*-*Cre* were employed for elucidating the effects of *Ezh2* on RGC development, survival and gene expression.

## Materials and methods

### Generation of mice with conditional knockout of *Ezh2* in RGCs

All animals were housed in the animal facility with 12 h light/dark cycle, and all animal procedures were approved by the Institutional Animal Care and Use Committee at the Schepens Eye Research Institute and were performed according to the ARVO Statement for the Use of Animals in Ophthalmic and Vision Research. Adult mice were sacrificed using CO_2_ exposure followed by cervical dislocation. Adult wild-type (WT) C57BL/6J mice were purchased from Jackson laboratory (Cat. No. 000664; Bar Harbor, ME.). *Ezh2*^*flox/flox*^ mice were acquired (RRID:MMRRC_015499-UNC from NIH/MMRRC) as previously reported [8]. In this mouse line, the *loxp* sites are flanking exons 16 to 19 which encode the essential SET domain of Ezh2 protein. The *Cre* recombinase knock-in mice driven by *Math5* promoter (*Math5-Cre*) were a gift from Dr. Lin Gan (Center for Aging and Developmental Biology, University of Rochester) [21, 24, 28, 29]. *Math5-Cre* mice were crossed with *Ezh2*^*flox/flox*^ mice to generate homozygous *Ezh2* knockout from Math5-expressing cells (*Math5-Cre;Ezh2*^*flox/flox*^ or mKO). In all experiments, littermate *Ezh2*^*flox/flox*^ mice were used as controls if not stated otherwise. Mouse genotypes were determined by polymerase chain reaction (PCR), using mouse tail DNAs. Briefly, mouse tails were incubated in a PCR buffer (Cat. No. 102-T; Viagen Biotech Inc., Los Angeles, CA.) containing 8 unit/ml proteinase K (Cat No. AM2548; Invitrogen, Grand island, New York) to extract mouse genomic DNA according to the manufacturer’s instruction. One μl of genomic DNA from mouse tails and primers for *Math5-Cre* (F: CCAGCTAAACATGCTTCATCGTC, R: TCTACACCTGCGGTGCTAACCA; 10 μM) were added to the Hotstart PCR Master Mix (Cat. No. DP-008-0250; eEnzyme, Gaithersburg, MD). The PCR was performed at a thermocycler with the following protocol: 95°C for 2 min, 35 cycles at 94°C for 20s, 56°C for 30s, 72°C for 30s, and 72°C for 5 min, which yielded a PCR product of 351 bp. To detect the *Ezh2* floxed gene, one μl of genomic DNA from a mouse tail and primers for *Ezh2* (F: CTGCTCTGAATGGCAACTCC; R: TTATTCATAGAGCCACCTGG) were added to a mixture of solution containing Apex TaqDNA Polymerase (Cat. No. 42–409), Apex buffer, and MgCl_2_ from Genesee Scientific (San Diego, CA), and dNTP (Cat. No. 10297–018; Invitrogen). The PCR was performed at 95°C for 2 min, 35 cycles at 94°C for 30s, 56°C for 30s, 72°C for 60s, and 72°C for 5 min (PCR products: WT: 430bp, floxed: 470bp). All PCR products were differentiated on a 2% agarose gel.

### Western blot

The quantification of Ezh2 protein in RGCs was assessed using protein lysates of purified RGCs as previously described [18]. Briefly, RGCs purified from new born (P0) mouse pups were lysed by sonication in ice-cold RIPA buffer (Cat. No. 20–188; Milipore, Billerica, MA) containing proteinase cocktail inhibitor (Ref: 05892953001; Roche, Indianapolis, IN) and phosphatase inhibitor cocktail (Cat. No. 78420; Thermo Scientific, Waltham, MA). Protein concentration was measured using a NanoDrop 2000 Spectrophotometer (Thermo Scientific). Two μg of protein from each sample were added to a gel electrophoresis and transferred to a nitrocellulose membrane. The blots were incubated with primary antibodies against Ezh2 (1:200; Cat. No. 5246S; Cell Signaling Technology, Danvers, MA) and GAPDH (glyceraldehyde 3-phosphate dehydrogenase (1:1000; Cat. No. 3683S; Cell Signaling Technology) as a loading control in a solution containing 5% non-fat dry milk and 0.05% Tween-20 overnight. The blot was incubated with goat anti-rabbit 680LT antibody (1:600; Cat. No. 827–11081; LICOR Inc, Lincoln, NE). The chemiluminescent signals were recorded by Odyssey Imager (LI-COR^®^ Inc, Lincoln, NE).

### Electroretinography (ERG)

Retinal functions of control and mKO mice were assessed by ERG as previously described [8]. Briefly, mice were dark adapted overnight and anesthetized by intraperitoneal injection (i.p.) of a ketamine-xylazine mixture (1:5; 5 μl/g bodyweight). Pupils were dilated topically by tropicamide (1%; Cat No. NDC 61314-355-01; Falcon Pharmaceuticals, Fort Worth, TX). A recording electrode was placed on the center of the cornea, with two grounding electrodes placed subcutaneously (s.c.) in the mid-frontal area of the head and the back area near the tail, respectively. To assess the functions of rod photoreceptors, mice were subjected to scotopic stimulation that was delivered at the light intensities of 0.0002, 0.02, 2, 200, and 600 cd•s/m^2^ through Xenon light; functions of cones were assessed using photopic stimulations that were delivered at 600 cd•s/m^2^, with a green light intensity of 13 cd•s/m^2^ and blue light of 1 cd•s/m^2^ in sequence. Flicker tests were assessed under a 6,500K white light stimulation at 15 cd•s/m^2^ and a frequency of 3, 10, and 15Hz, respectively. The data were recorded and processed by the ERG system (Espion Electroretinography System; Diagnosys LLC, Lowell, MA).

### Spectral-domain optical coherence tomography

Retinal laminar morphology and thickness of the ganglion cell complex (GCC) were assessed in live mice non-invasively using spectrum domain optical coherence tomography (SD-OCT) as established in our lab [30]. GCC includes the nerve fiber layer, ganglion cell layer (GCL), and inner plexiform layer. Mice were anesthetized by i.p. injection of a ketamine/xylazine mixture, and pupils were dilated using 1% tropicamide. Lubricant gel drops (Novartis Pharmaceuticals Corp, East Hanover, NJ) were applied to maintain the moisture of the cornea. Images were acquired using SD-OCT (InVivoVue Clinic; Bioptigen Inc, Research Triangle Park, NC), and 100 radial volume scans covering 360° of the retina (centered on optic disc, diameter 1.3 mm) were collected. The GCC thickness was assessed automatically with Diver 2.0 software (Bioptigen Inc, Research Triangle Park, NC), measured at four points in each scan (200 and 400 μm from the central of the optic disk at both sides, respectively) and averaged from 100 scans of each retina.

### Immunofluorescence labeling

Eyeballs were dissected and fixed in 4% paraformaldehyde (Cat. No. BM-698; Bostonbioproducts, BioProducts, Ashland, MA) for 2 hours at room temperature followed by immersing into 20% sucrose solution in phosphate buffered saline (PBS) for 2 hours. The eyeballs were embedded in O.C.T. compound (Cat. No. 4583; Sakura Finetek USA, Inc., Torrance, CA) on dry ice. Frozen sections of the retina (10 μm) were incubated with a blocking buffer containing 4% bovine serum albumin and 0.5% Triton X-100 in PBS for 1 hour followed by incubation with a primary antibody against β-III-tubulin (1:500; Cat. No. MAB5564, Millipore), Recoverin (1:500; Cat. No. AB5585, Millipore) or H3K27me3 (1:500; Cat. No. 9733S, Cell Signaling Technology, Danvers, MA) in the blocking buffer for overnight at 4°C. Slides were washed with PBS 3x at 10 min. each before incubation with Cy3/Cy2-conjugated secondary antibody in the blocking buffer [Cy3-AffiniPure Donkey Anti-Mouse (1:500; Cat. No. 715-165-151, Jackson ImmunoResearch) or Cy2-AffiniPure Donkey Anti-Rabbit (1:500; Cat. No. 711-095-152, Jackson ImmunoResearch)] were applied for 1 hour at room temperature. Slides were washed with PBS 3 x at 10 min. each. The slides were mounted in a mounting media containing DAPI (Cat. No. H-1200; Vector Laboratories Inc.) and imaged with a TSC SP5 confocal microscope (Leica Microsystems, Richmond, IL).

### RGC counts in retinal flat-mounts

RGC quantification was carried out as we previously described [31]. The mouse retina was dissected and flat mounted. RGCs were double labeled with anti-β-III-tubulin and DAPI. The specimens were visualized and photographed under a confocal microscope. For RGC counting, retinal flat-mounts were divided into quadrants using the optic nerve head (ONH) as the origin: superior, temporal, nasal and inferior. Within each quadrant, four squares (198 μm × 198 μm) distributed at a 1 mm interval along the radius were selected: one from the peripheral region (2 mm from the ONH), two from the intermediate region (1 mm from the ONH), and one from the central region. A total of 16 square regions of each eye were photographed, and all β-III-tubulin+ cells in the GCL were counted. Average RGC densities of the entire retina were calculated, and the percentage of RGC loss was determined by comparing RGC densities with that obtained from the contralateral control eyes.

### RNA sequencing (RNA-seq) and quantitative real-time PCR (qPCR)

RGCs from new born (P0) WT and mKO mice were purified as previously described [32]. In brief, dissociated retinal cells were incubated with a magnetic bead conjugated anti-Thy-1 antibody, and RGCs were purified following the manufacturer’s instruction. For RNA-seq gene profiling, RNAs were extracted using an RNeasy Plus Mini Kit (Cat. No. 74134; Qiagen, Limburg, Netherlands). Each group of RNA samples contained a triplicate from 3 independent RNA extractions. The quantity and quality of total RNAs were tested using a Nanodrop 2000 spectrophotometer (Cat No. ND-2000; Thermo Scientific, Cambridge, USA), followed by verification on an Agilent 2100 BioAnalyzer. RNA was then reverse-transcribed into cDNA using One-Cycle cDNA synthesis kit (Affymetrix, Santa Clara, CA, USA) according to the manufacturer’s instructions. The RNA-seq study was carried out in the Center for Cancer Computational Biology, Dana-Farber Cancer Institute, Boston, MA. Samples were prepared for sequencing using the NEBNext Ultra RNA Library Prep kit after isolating mRNA using a poly-A bead based selection. Each library was normalized to a concentration of 2 nM to create a multiplex pool for sequencing. The final denatured and diluted pool was loaded onto the NextSeq at a concentration of 2 pM and ran on Single Read (SR50) flowcells with inputs not lower than 100 ng total cDNA/sample. Sequencing was completed on the NextSeq500, using a high-throughput single-end 75 cycle flowcell. All 12 libraries were loaded into one lane for sequencing. Each library returned an average of 40–50 million reads. Alignments were carried out with STAR aligner (version 2.3) [33] against the mm10 genome available at ftp://ftp.ensemble.org/pub/release-75/fasta/mus_musculus/dna/. RNA-seq quality metrics controls were accessed by the Broad Institute’s RNA-SeQC tool [34]. Read quantification was carried out with featureCounts [35]. Read normalization and Differential expression testing were performed with DESeq package in R [36], and sequence data quality was assessed based on FastQC package. Network analysis of differentially regulated genes was performed using GO (Gene Ontology enrichment analysis and visualization tool; https://david.ncifcrf.gov/) [37].

For qPCR, total RNAs from purified RGCs were converted to cDNA using SuperScript Reserve Transcriptase (Cat. No. 18080–400; Life Technology) according to the manufacturer’s instruction. RT-PCR was performed in Roche LightCycler^®^ 480Ⅱ(Roche Biosystems; Indianapolis, IN) using SYBRGreen fast qPCR Master Mix (Cat. No. KK4611; KAPA Biosystems, Wilmington, MA, USA) with specific primers listed in Table 1. Samples were analyzed in duplication, and the relative amounts of mRNAs were calculated by normalizing to GAPDH expression level. The entire RNA-seq data was uploaded on NCBI GEO, with the Accession Number: GSE93674.

**Table 1:** Primer sequences for RT-PCR.

	Forward	Reverse
***GAPDH***	AACTTTGGCATTGTGGAAGG	ACACATTGGGGGTAGGAACA
***Six1***	CTTGTACATAGAAGCCAGGGACAA	AGGGACTACTGTAAAGGATGCC
***Cralbp***	CTGTCCAGGGTGGAGGTCAT	CCCCAGCACCAAGGATCAC
***Tuj1***	CCAAGTTCTGGGAGGTCATC	TGAGAGGAGGCCTCATTGTAG
***Brn3a***	CTCACGCTCTCGCACAAC	AGAGCTCCGGCTTGTTCAT
***Math5***	CAGGACAAGAAGCTGTCCAA	CATAGGGCTCAGGGTCTACCT
***Sox2***	AGAACCCCAAGATGCACAAC	CTCCGGGAAGCGTGTACTTA
***Pax6***	AACAACCTGCCTATGCAACC	ACTTGGACGGGAACTGACAC
***Eya1***	CGTCCACCAATGCCACTTAC	GTGGAAAACAATGATGGTCTCGT
***Eya2***	CTCCCTGAAAGCCCTCAATC	TGTCTTGGTCGCACTGTAGATG
***Ezh2***	TGGTGGATGCAACCCGAAAG	ACTCTTCACCAGTCTGGATAGC
***Rhodopsin***	CATGCCAATATGCCCACCTT	GCACTGTGTTTCTGAACTCTTCAGA
***Recoverin***	GCAGCTTCGATGCCAACAG	TCATGTGCAGAGCAATCAGGTA
***Ezh1***	TGTGAAAAGTTCTGCCAGTGC	CACACTCACGAACTGCCAAG

### Microbeads induced glaucoma mouse model and IOP measurements

Our previous study [31] described a simple and reproducible method to induce high IOP and glaucoma in the rodent eye by injecting polystyrene microbeads into the anterior chamber. Briefly, three-month-old (M) mice were anesthetized by i.p. injection of a ketamine-xylazine mixture and supplemented by topical proparacaine hydrochloride (0.5%; Bausch & Lomb, Tampa, FL). Pupils were dilated with 1% tropicamide solution. Microbeads (Cat. No. F884; Life Technology) were resuspended in sterile saline at a final concentration of 12 × 10^6^ beads/ml. The cornea of the right eye was punctured using a 30G needle. Two μl of 15 μm diameter polystyrene microbeads were injected into the anterior chamber via a glass micropipette which was connected to a Hamilton syringe; 1% chloramphenicol eye ointment was applied onto the cornea immediately following microbead injection. The contralateral eye that received 2 μl PBS injection was served as a control. The IOP was measured by tonometer (TonoLab; Colonial Medical Supply, Espoo, Finland) prior to microbead injection (day 0) and twice a week as previously described [38]. The tonometer takes six measurements and displays an average after elimination of high and low readings. We considered this machine-generated average as one reading; ten readings were obtained from each eye, and the means of ten readings were calculated to determine the IOPs. Mice were euthanized on day 28 post-microbead injection.

### Optic nerve crush injury

The optic nerve of the right eye was exposed and crushed with fine forceps for 5 seconds at 1–2 mm posterior to the optic nerve head [39]. Immediately following the injury, mice were given buprenorphine (50 μl/g body weight (Cat. No. 12496-0757-1; Reckitt Benckiser Pharmaceuticals Inc., Parsippany, NJ) by s.c. injection every 8 to 12 h for 24 h. On day 14 post-injury, mice were sacrificed. The optic nerves were collected and processed for semi-thin sections, and the retinas were processed for immunolabeling. For optic nerve counting, mouse optic nerve samples were fixed with half strength Karnovsky’s fixative (2% paraformaldehyde + 2.5% glutaraldehyde in 0.1 M sodium cacodylate buffer, pH 7.4; Electron Microscopy Sciences, Hatfield, Pennsylvania) for a minimum of 24 hours. Optic nerve samples were rinsed with 0.1 M sodium cacodylate buffer and post-fixed in 2% osmium tetroxide in 0.1 M sodium cacodylate buffer. Following *en bloc* staining with 2% uranyl acetate in distilled water, the samples were dehydrated with graded ethyl alcohol solutions through transition with propylene oxide and resin, in which samples were infiltrated in tEPON-812 epoxy resin (Tousimis, Rockville, Maryland) using an automated EMS Lynx 2 EM tissue processor (Electron Microscopy Sciences, Hatfield, Pennsylvania). Processed tissues were oriented in tEPON-812 epoxy resin and polymerized for 48 hours in silicone molds in an oven set for 60°C. Semi-thin cross-sections were cut at 1-micron with a Histo diamond knife (Diatome, Hatfield, Pennsylvania) on a Leica UC-7 ultramicrotome (Leica Microsystems, Buffalo Grove, IL), collected on slides, and dried on a slide warmer. The slides were then stained with 2% aqueous paraphenylenediamine (MP Biomedicals LLC, Solon, Ohio) solution at room temperature, rinsed in tap and deionized water solutions, air-dried, and mounted with a glass coverslip before light microscopic analysis and nerve counting.

### Statistical analysis

All numerical variables in this article were presented as Mean ± Standard Error of Mean (SEM). The two-sided student's *t*-test was used on numerical variables of independent samples; one-way ANOVA analysis was applied for comparisons of data among three or more groups. P-value < 0.05 was considered statistically significant. Volcano plot was created with ggplot2 in R. Statistical analysis in RNA-seq was carried out as stated above. GO analysis was carried out with DAVID (Database for Annotation, Visualization and Integrated Discovery) from http://david.abcc.ncifcrf.gov/.

## Results

### RGCs develop normally in mKO mice

**Fig 1:**
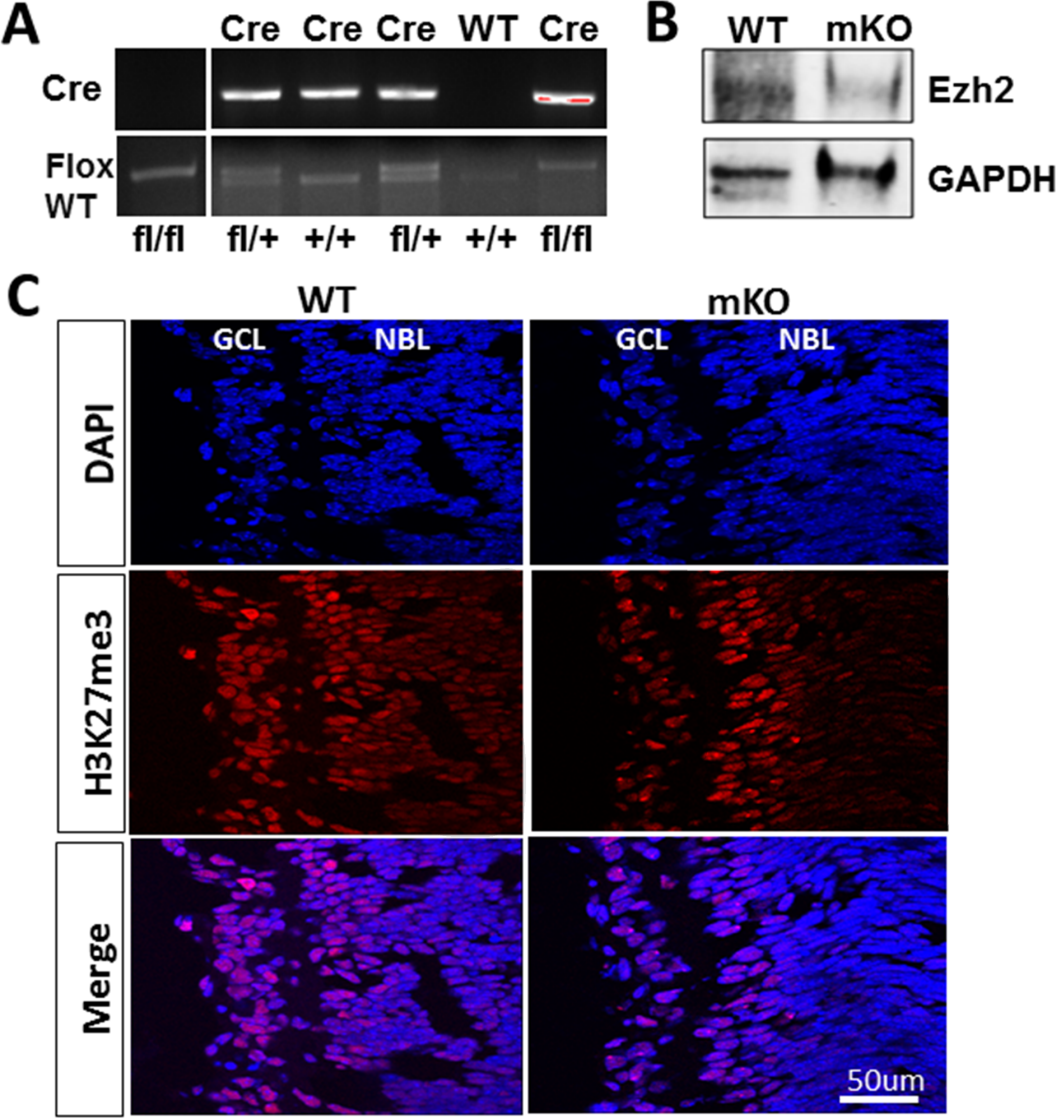
Deletion of *Ezh2* driven by *Math5-Cre* in mKO mice. **(A)** PCR genotyping of *Ezh2* and *Cre* genes. **(B)** Representative result of Western blot of Ezh2 expression in RGCs purified from P0 WT and mKO mice. GAPDH was used as a loading control. A strongly reduced level of Ezh2 was found in mKO RGCs as compared to WT RGCs. (**C)** Epifluorescence images of retinal sections taken from P0 WT and mKO mice that were immunolabeled for H3K27me3 (red) and nuclear marker 4’,6-Diamidino-2-Phenylindole (DAPI; blue). Note the higher level of H3K27me3 signals in the mKO retina compared to WT retina. Scale bar: 50 μm.

**Fig 2:**
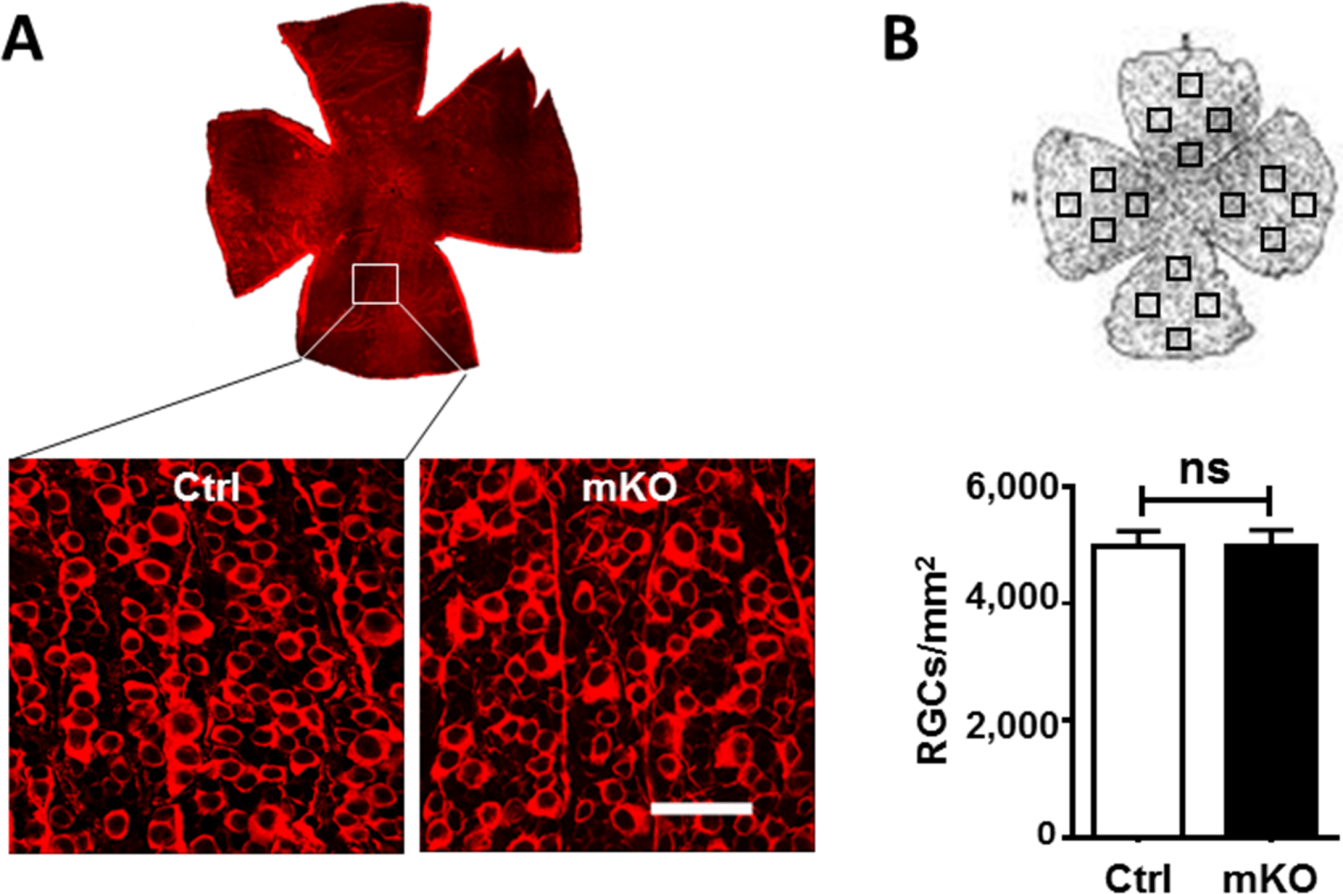
Similar RGC densities in adult mKO and littermate floxed control mice. **(A)** Representative en face (top) and β-III-tubulin-immunolabeled retinal flat-mounts taken from littermate floxed control and mKO mice; scale bar: 50 μm. **(B,C)** Schematic illustration of RGC quantification (B) and RGC densities in adult littmate floxed and mKO mice (C; n = 6/group). No significant difference (NS) was noted between the floxed and mKO mice (*P* = 0.98; two-tailed student *t* test).

To investigate the roles for *Ezh2* in RGC development and function, we generated Math5-driven *Ezh2* knockout by crossing *Math5-Cre* with *Ezh2*^*flox/flox*^ mice (mKO). Mouse genotypes were determined by PCR with tail DNA (Fig 1A). High levels of *Cre* expression were detected in E16 –P0 RGCs of mKO, but not WT, mouse pups (S1 Fig). Consequently, P0 RGCs of mKO mice revealed largely diminished levels of *Ezh2* compared to WT mouse pups, as demonstrated both by qPCR (S1 Fig) and Western Blot (Fig 1B). Immunolabeling of *H3K27me3* confirmed downregulation of its signal selectively in the GCL of mKO mouse pups as compared to P0 WT control mice (Fig 1C). In agreement with the reports that ~50% RGCs were positive for *Cre* detection in *Math5-cre* mice [27], our result confirmed that some RGCs of mKO mice indeed retained *H3K27me3* signals. Despite the diminished *H3K27me3* deposition in RGCs, mKO mice survived to adulthood without apparent growth or morphological defects in the retina. Immunolabeling of RGC marker β-III-tubulin and photoreceptor marker Recoverin in retinal sections showed comparable patterns in *Ezh2*^*flox/flox*^ littermate controls and mKO mice up to 12 months of age (S2 Fig). RGC densities in *Ezh2*^*flox/flox*^ littermate control and mKO mice were 4,988 ± 254/mm^2^ and 4,997 ± 260/mm^2^, respectively, and no significant difference in RGC counts was noted (Fig 2A–2C). Thus, selective deletion of *Ezh2* driven by *Math5*-*Cre* does not affect RGC survival.

### The retinas of mKO mice have normal light-induced ERG responses

**Fig 3:**
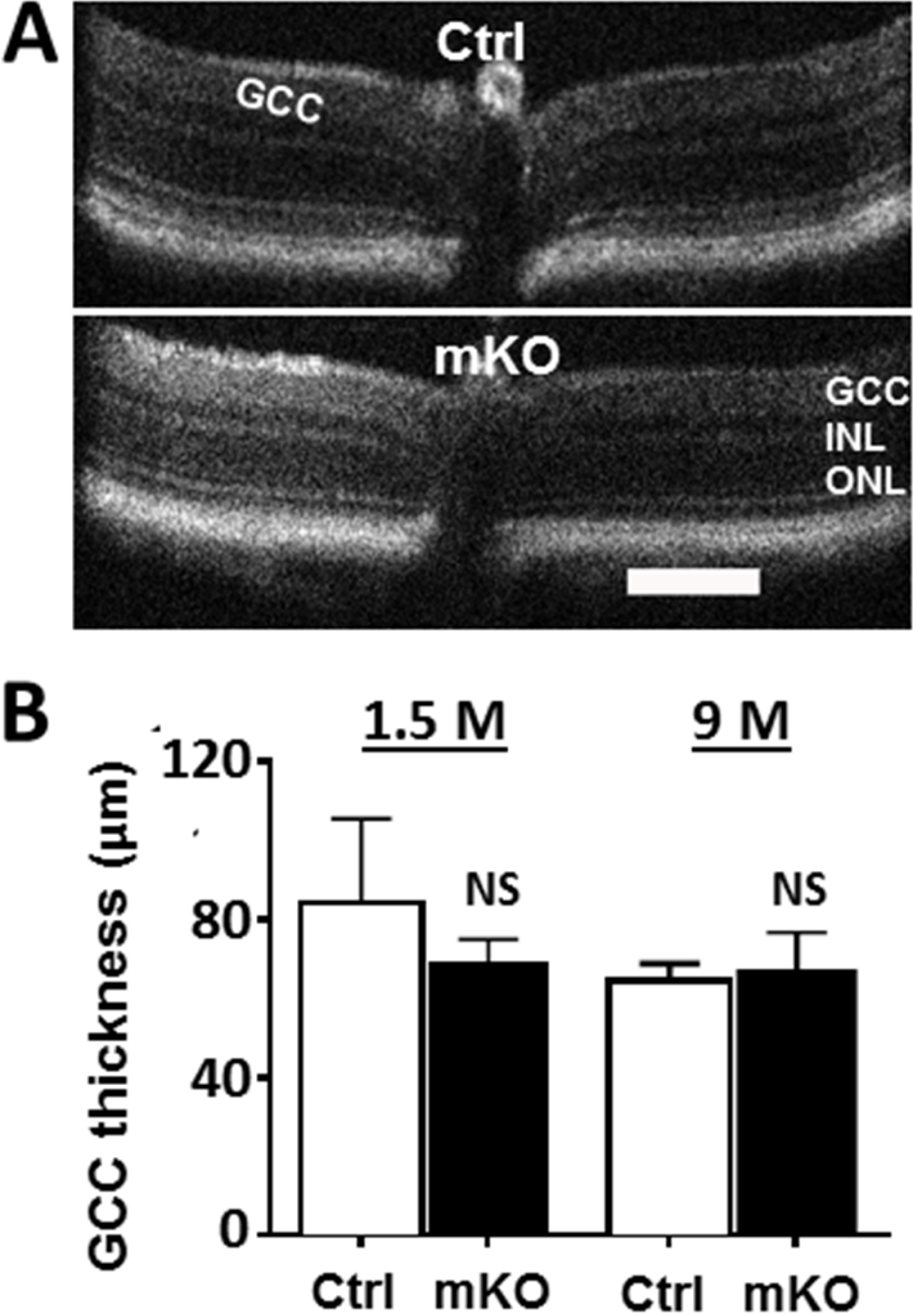
Retinal morphology in mKO and littermate control mice assessed by SD-OCT. **(A**) Representative radial volume scans of the floxed control and mKO mouse retinas with SD-OCT. No apparent defects were noted. Scale bar: 200 μm **(B)** Quantification of GCC thickness. No significant difference (NS) was noted between the floxed and mKO mice at either 1.5 or 9 month old (n = 8/group; *P* > 0.05 by two-tailed student *t* test).

**Fig 4:**
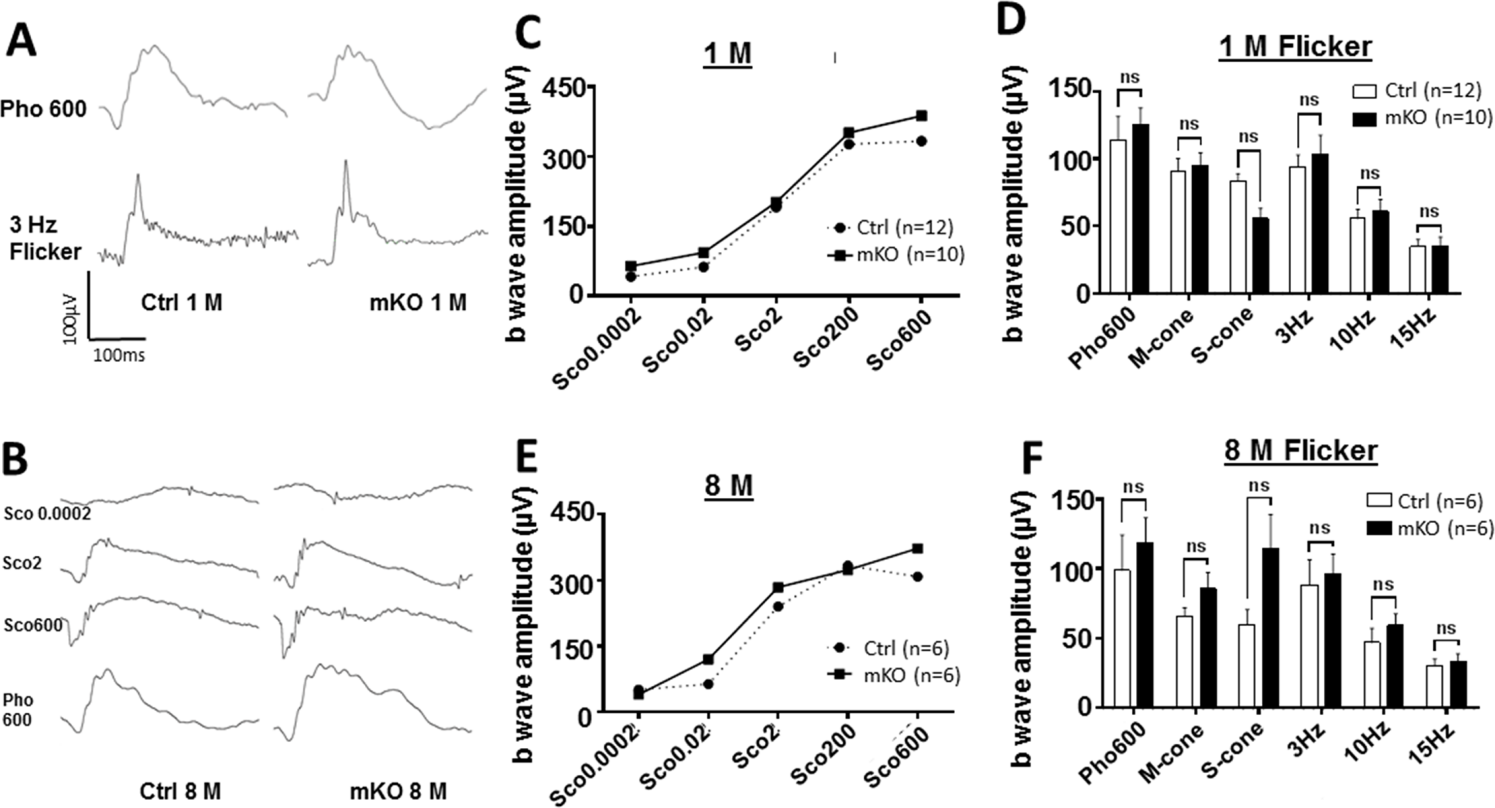
ERG assessment for retinal function. **(A,B)** Representative ERG waveforms in floxed control and mKO mice that were subjected to photopic (A) or scotopic (B) flashes of increasing intensities. **(C-F)** Amplitudes of ERG b-waves assessed at 1 (C, D) and 8 (E, F) month-old mice. NS indicates no significant difference (n = 10/group at 1M, n = 6/group at 8M; *P* > 0.05 by two-tailed student *t* test).

We previously reported that mice carrying embryonic deletion of *Ezh2* in *Chx10*-positive retinal progenitors were born with normally structured retina but developed progressive photoreceptor degeneration in postnatal life [8]. To investigate if deletion of *Ezh2* in developing RGCs causes any progressive changes in the postnatal retina, we non-invasively tracked retinal morphology and function in live mice using SD-OCT and ERG from 1 to 9 months. Nevertheless, we detected no apparent malformation of the GCL or retinal laminar structure in mKO mice (Fig 3A). It is suggested that combined thickness of the nerve fiber layer, GCL, and inner plexiform layer, together defined as GCC, has diagnosis capability for RGC and/or axon degeneration [30, 40–44]. Our quantification results showed that the GCC thicknesses of littermate controls at 1 and 9 months-old were 84.2 ± 6.7 and 64.6 ± 1.5 μm, and those of mKO mice at 1 and 9 month old were 69.3 ± 1.9 and 67.3 ± 4.3 μm, respectively; no significant difference between the control and mKO mice, at either 1 or 9 month-old, was noted (Fig 3B). The evaluation of light-induced retinal activities with ERG also did not reveal any apparent abnormalities in the photopic or scotopic responses (Fig 4A and 4B); quantification of scotopic and photopic b wave amplitudes showed no significant differences between control and mKO mice at either 1 month or 8 months-old (Fig 4C–4F). These data suggest that targeted deletion of *Ezh2* from RGCs does not affect the normal function and morphology of RGCs, which supports the notion that *Ezh2* mediates retinal homeostasis in a cell lineage-specific manner.

### Targeted deletion of *Ezh2* does not affect RGC susceptibility to elevated IOP- or optic nerve injury-induced damage

**Fig 5:**
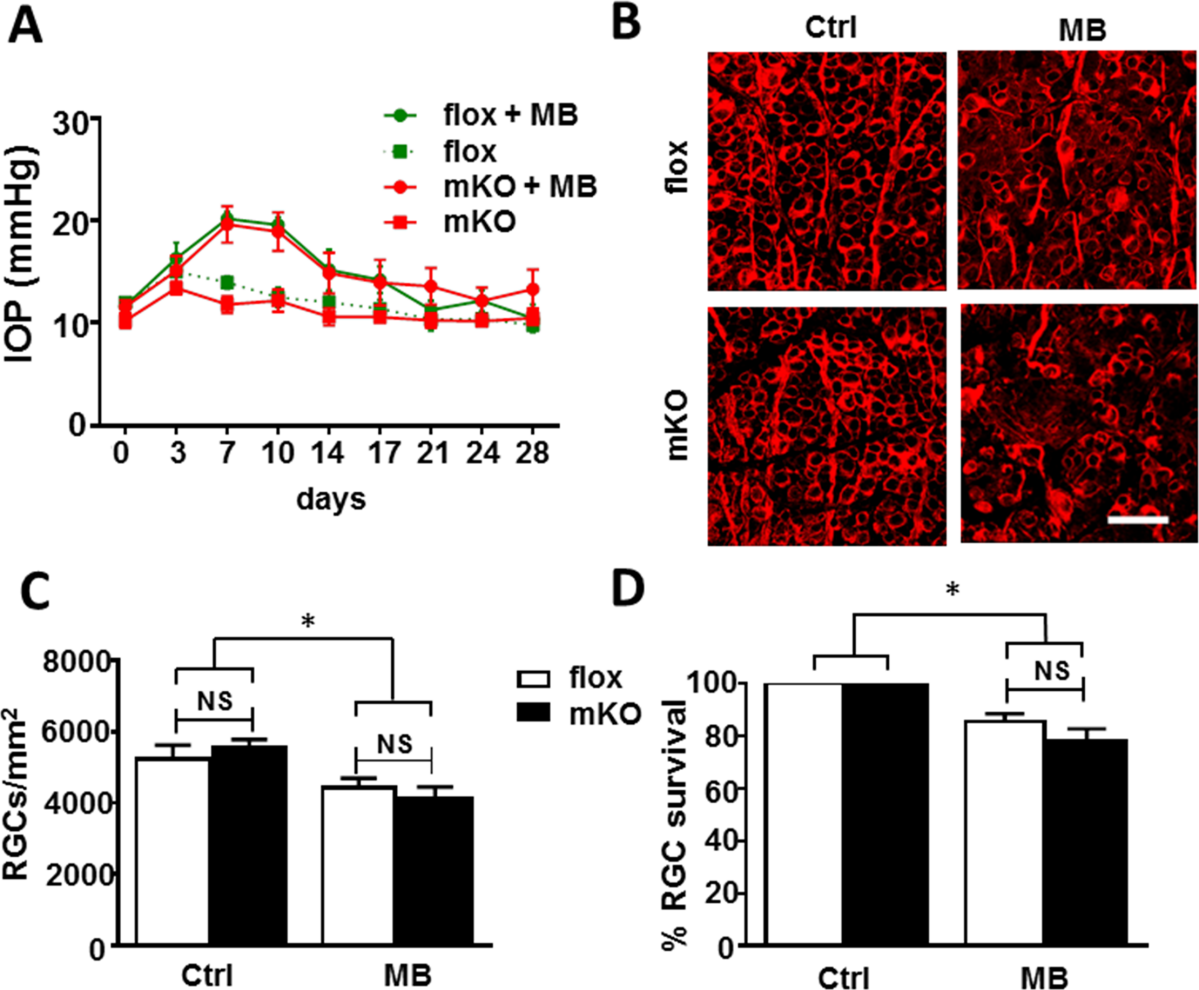
Comparable RGC loss under ocular hypertension in control and mKO mice. **(A)** Assessment of intraocular pressure (IOP) following injection of microbeads into the anterior chamber in littermate floxed control (flox + MB) and mKO mice (mKO + MB); contralateral eyes that were injected with PBS were served as normal IOP controls (flox or mKO). **(B)** Representative images of retinal flat-mounts stained with anti-β-III-tubulin to reveal RGC morphology. Scale bar: 50 μm. **(C, D)** RGC densities (C) and survival rates (D) following induction of ocular hypertension in floxed control (white bar; n = 6) and mKO (black bar; n = 15) mice. While ocular hypertension induced significant RGC loss in both littermate control and mKO retinas (*P < 0.05 by one-way ANOVA), no significant difference (NS) in RGC densities (C) and survival rates (D) were noted between littermate control and mKO mice (*P* = 1.00 and 0.56, respectively by one-way *ANOVA*).

**Fig 6:**
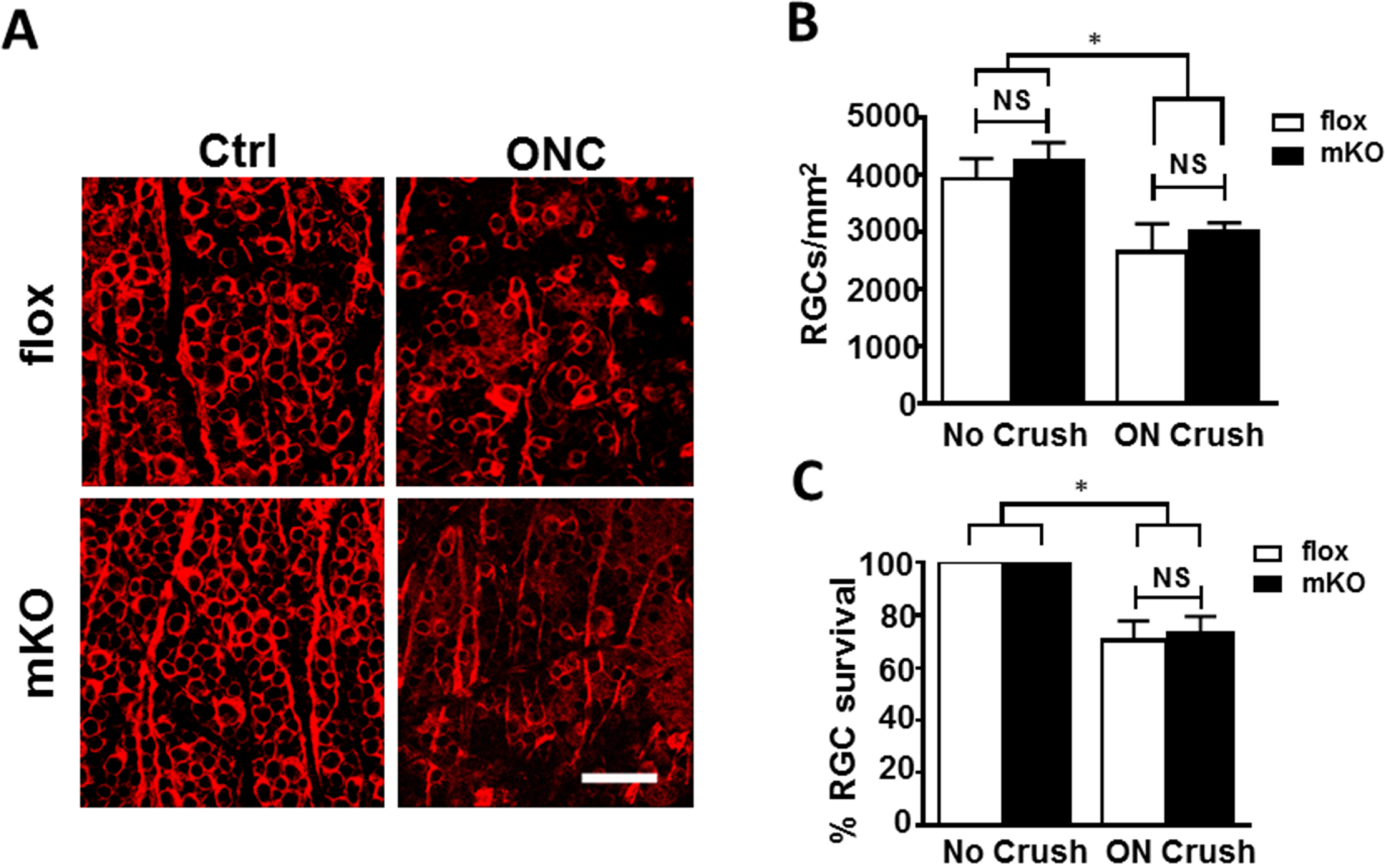
Comparable optic nerve crush injury-induced axon loss in control and mKO mice. **(A)** Representative images of retinal flat-mounts taken from littermate floxed control and mKO mice with (ON crush) or without (Ctrl) optic nerve crush injury that were immunolabeled for β-III-tubulin to reveal RGC morphology. Scale bar: 50 μm. **(B,C)** Quantifications of RGC loss density (B) and survival rate (C) following optic nerve crush injury in WT (white bar; n = 4) and mKO (black bar; n = 7) mice. (*P < 0.05; NS *P >* 0.05 one-way ANOVA).

Dysregulation of *Ezh2* function in development has been associated with adult-onset neurodegeneration [45, 46]. We therefore asked if its deletion alters RGC vulnerability to optic nerve injury or disease, such as glaucoma, a leading cause of blindness worldwide. Adult floxed littermate control and mKO mice were subjected to microbead injection to induce elevated intraocular pressure (IOP); the contralateral eyes were injected with PBS to serve as control. Before microbead injection, the baseline IOP levels were assessed, which averaged 10–12 mmHg (OD) in both control and mKO mice. A single injection of microbeads induced a similar kinetic of IOP elevation in mKO and control groups, peaking at day 7 post injection (20.3 ± 0.7 mmHg in control, 19.6 ± 1.8 mmHg in mKO) and gradually returning to the baseline by ~28 days post injection (Fig 5A). The IOP of uninjected contralateral eyes of mKO and littermate control mice showed no significant change and remained steady throughout the period (averaged 11.95 ± 0.32 mmHg in control and 11.09 ± 0.26 mmHg in mKO mice). Microbead injection induced comparable (~20%) reduction of RGC densities or survival in both mKO and littermate control mice in 4 weeks (Fig 5B–5D). RGC densities in WT and mKO mice with a normal IOP were 5,220 ± 394 cells/mm^2^ and 5,553 ± 223 cells/mm^2^, and RGC densities of littermate control and mKO mice 4 weeks after IOP elevation were 4,421 ± 266 cells/mm^2^ (or 85.3 ± 3.1% survival) and 4,097 ± 345 cells/mm^2^ (or 78.0 ± 4.8% survival) respectively. No significant difference of RGC loss was noted between littermate control and mKO mice, suggesting that their RGCs are similarly susceptible to elevated IOP-induced neuronal damage. We next introduced optic nerve crush injury, which presents a more severe and acute insult to RGCs than microbead injection [47]. At 14 days post optic nerve crush injury, RGC densities in littermate control and mKO mice before injury were 3,935 ± 360 and 4,248 ± 325 cells/mm^2^, those after optic nerve injury in littermate control and mKO mice were 2,646 ± 503 (or a 70.3 ± 7.4% survival rate) and 3,001 ± 162 cells/mm^2^ (or a 73.0 ± 6.5% survival rate), respectively. The floxed littermate control and mKO mice exhibited similar extent of neurodegeneration as compared to their uninjured contralateral eyes (Fig 6A–6C). No significant difference of RGC degeneration was noted between mKO and control mice. Thus, *Ezh2* deficiency driven by *Math5-*Cre does not alter RGC susceptibility to optic nerve injury.

### Targeted deletion of *Ezh2* caused few RGC-related gene expression changes

**Fig 7:**
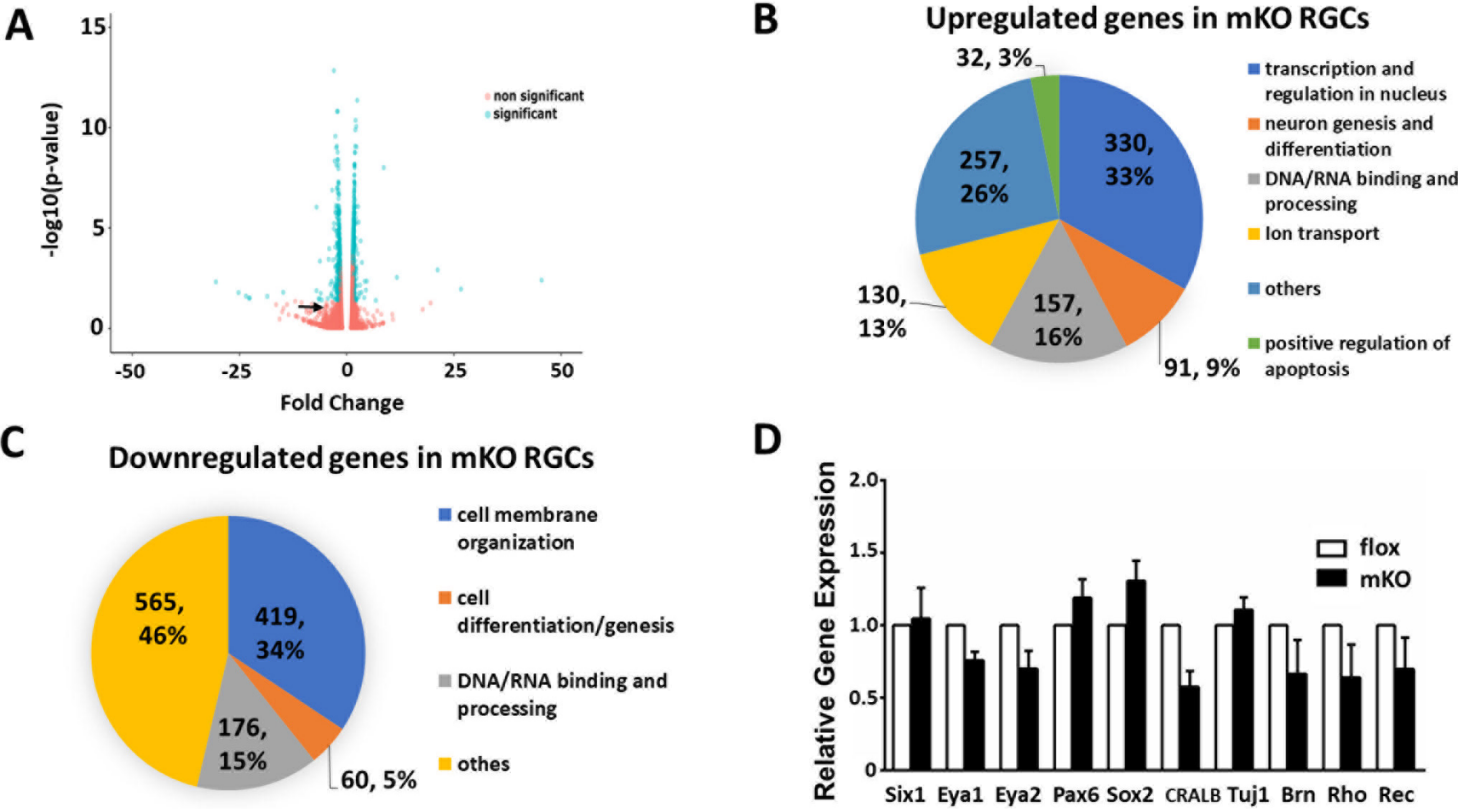
Gene Ontology analysis for differentially expressed genes in WT and mKO mice. **(A)** Volcano plot showing fold changes (fc) of all genes detected from RGCs of mKO mice against control mice. Statistical significance (green dots) was defined as *P* <0.05 with a fold change ≥ +1.5 or ≤ -1.5 when compared to WT; non-significant changes (orange dots) fulfill either one or none of these two criteria. 997 genes were found with fc ≥ +1.5 and 1,220 genes with fc ≤ -1.5. Arrow points to the *Ezh2* site. **(B,C**) Pie charts represent depicted GO terms for upregulated **(B)** and downregulated **(C)** genes with │fc│ ≥ 1.5. No GO term in either up- or downregulated gene group were found to be specifically related to eye development. (**D)** RT-PCR verification of mRNA levels of retinal related genes in RGCs purified from P5 floxed littermate control (white bar; n = 4) and mKO (black bar; n = 7) mouse pups. CRAL: *Cellular retinaldehyde binding protein*; Tuj1: *βIII-tubulin (Tubb3)*; Brn: *Brn3a (Pou4f1)*; Rho: *Rhodopsin*; Rec: *Recoverin* (*P < 0.*05* by one-way *ANOVA*).

To further determine the impact of targeted *Ezh2* deletion on RGCs, we performed RNA-seq gene profiling with RGCs isolated from P0 pups, when is the earliest time point that differentiated RGCs can be efficiently isolated from the mouse retina. The full list of genes identified with RNA-seq was uploaded in the NCBI data base with an assigned accession number as GSE93674. Among 13,549 genes detected, we identified 997 significantly upregulated and 1,220 downregulated genes at a cut-off of 1.5 fold changes (fc) with a p-value of *P < 0*.*05* in *Ezh2*-deficient RGCs as compared to littermate control RGCs (Fig 7A and S1 Table). In agreement with that ~50% RGCs of *Math5-Cre* mice were detected positive for Cre [24], the result of RNA-seq revealed ~1 fold change in *Ezh2* level in RGCs of P0 mKO mice compared to WT mice (Fig 7A). With Gene Ontology (GO) analysis [37, 48], differentially expressed genes were shown to generally relate to transcription regulation, membrane organization, and DNA/RNA binding (Fig 7B and 7C). Among them, 33% of 997 detected upregulated genes were mapped to the keyword ‘transcription regulation and nucleus”, 16% were related to DNA or RNA binding and processing. Notably, 32 out of 997 (3%) genes were associated with apoptosis (Fig 7B). Among 1,220 downregulated genes, 34% showed annotation to cell membrane organization, 15% to DNA/RNA processing and 5% to cell differentiation and genesis (Fig 7C). However, none of the genes that made to the 1.5 fold cut-off line were found to associate with GO terms specifically related to RGC development or retinal functions. IPA (Ingenuity Pathway Analysis) (QIAGEN Inc., https://www.qiagenbioinformatics.com/) was also applied to analyze the same pool of differentially expressed genes (DEG). In agreement with GO analysis, IPA revealed upregulation in pathways and functions that have not been associated with the retina, but some appeared to associate with tumorogenesis or gastrointestinal diseases. For instance, *Tktl1* (+24 fc) and *Capn11* (+18.5 fc), the most upregulated genes, were found in malignant tumors of ocular adnexa [49] or belonged to the family of calcium-activated neutral proteinases. *Umod* (-58 fc), *Kap* (-50 fc) and *Aldob (*-9 fc), which were among the most downregulated genes, were reported to mediate renal development. Yet, none of these genes were involved in retinal development, disease, or function.

Next, we pulled out 21 RGC-lineage specific genes, especially those known to be downstream of *Math5* [50, 51] (Table 2). By applying a cut-off of gene expression change over 1.5 fold and both q- and p-value over 0.05, 4 RGC-lineage genes barely passed this cut-off line, while the expression of other 17 genes were not significantly different in WT and mKO RGCs. RT-PCR was applied to further verify the RNA-seq results. Using RGCs isolated from P0 mKO and littermate controls, 18 genes, including 6 RGC-lineage genes listed in Table 2 (*Eya1*, *Eya2*, *Pou4f1*, *Sncg*, *Thy1*, and *Tubb3*) and retinal specific genes, such as *CRALBP* (Müller cell marker), *Rhodopsin* and *Recoverin* (photoreceptor cell markers), and retinal progenitor or development related genes, *Pax6* and *Sox2* were selected for further testing. We also tested *Six1*, which was identified as a direct target of *Ezh2* [1, 8], and its coactivators *Eya1* and *Eya2*. RT-PCR revealed no significant differences in the levels of these genes, including the 4 RGC-lineage genes that had passed the cut-off line in RNA-seq analysis, in RGCs of mKO mice compared to littermate controls (Fig 7D and not shown). Collectively, these results indicate no significant differences in the levels of expression of RGC-lineage specific genes between WT and mKO mice, supporting that *Ezh2* mediates suppression of certain fetal genes in a cell-lineage specific manner.

**Table 2:** Detection of RGC lineage related genes in RNA-seq.

Gene	Ensembl gene ID	mKO reads	WT reads	Fold change	p-value	q-value
Atoh7	ENSMUSG00000036816	64.78	152.90	-2.36	0.23000628	0.612234901
Ccnd1	ENSMUSG00000070348	2506.12	4050.32	-1.62	0.009271956	0.064024791
Eya1	ENSMUSG00000025932	234.66	198.52	1.18	0.201418606	0.565063613
Eya2	ENSMUSG00000017897	272.11	309.27	-1.14	0.262897825	0.662658338
Gli1	ENSMUSG00000025407	557.03	867.00	-1.56	0.308207147	0.722775334
Isl1	ENSMUSG00000042258	1728.85	1782.07	-1.03	0.694533191	0.970137019
Map2	ENSMUSG00000015222	6386.62	6704.21	-1.05	0.070014361	0.948427399
Mapt	ENSMUSG00000018411	6150.18	8922.69	-1.45	1.86E-05	0.000539374
Mstn	ENSMUSG00000026100	1526.35	1384.10	1.10	0.308573542	0.723314714
Nefl	ENSMUSG00000022055	2456.47	3276.35	-1.33	0.001262743	0.01407436
Neurod1	ENSMUSG00000034701	1406.83	1807.07	-1.28	0.021689304	0.12110204
Nrp1	ENSMUSG00000025810	2172.64	1693.14	1.28	0.046334564	0.209231488
Nrp2	ENSMUSG00000025969	1458.65	1543.71	-1.06	0.6877792	1.0000000
Pou4f1	ENSMUSG00000048349	2704.04	4192.92	-1.55	8.27E-07	4.95E-05
Pou4f2	ENSMUSG00000031688	2067.77	2817.67	-1.36	0.000500115	0.006899612
Six1	ENSMUSG00000051367	12.35	15.72	-1.27	0.690077596	1.0000000
Six3	ENSMUSG00000038805	2230.39	3329.67	-1.49	8.83E-06	0.000299378
Sncg	ENSMUSG00000023064	888.58	1328.46	-1.50	2.84E-05	0.000748377
Thy1	ENSMUSG00000032011	1424.39	2373.56	-1.67	2.48E-08	3.52E-06
Tubb3	ENSMUSG00000062380	1976.53	3174.79	-1.61	1.81E-07	1.54E-05
Vegfa	ENSMUSG00000023951	945.68	1232.08	-1.30	0.034151158	0.168474811

These 22 RGC-lineage genes were selected based on the reports by Mu et al. 2004, 2005 using *math5*-null or *Pou4f2*-knockout mice [50, 51]. The p-value was calculated with one way ANOVA. The q-value reflects the adjusted p-value that has been optimized using characteristics of p-value distribution or a FDR approach to more precisely predict the chance of false positives.

## Discussion

The roles of *Ezh2* have attracted significant attention due to its critical involvement in tissue growth, homeostasis, and cancer development. By selectively deleting *Ezh2* from Math5^+^ lineage progenitors driven through *Math5-*Cre in the present study, we showed that absence of *Ezh2* in the perinatal period has little impact on the expression of retinal specific genes or its direct target genes identified in photoreceptor progenitors, such as *Six1* [8]. Neither does this alter the maturation, function, and homeostasis of RGCs, nor does it affect their susceptibility to injury and stress in the adult.

Our findings are unexpected in light of the previous report which suggests a role for *Ezh2* in orchestrating photoreceptor homeostasis in postnatal life when mice with *Chx10-*Cre-driven deletion of *Ezh2* from retinal progenitors were used [8]. *Ezh2* does so by functioning at the nexus point of retinal progenitors to suppress transcription of specific fetal genes, such as *Six1*, and mediates the delicate balance between proliferation and maturation. Involvement of *Ezh2* in postnatal homeostasis has also been reported in cardiac and other cell types [1,8]. Given to the high enrichment of *Ezh2* in the perinatal GCL, we asked if *Ezh2* also plays a role in the survival and function of postnatal RGCs. To our surprise, selective deletion of *Ezh2* driven by *Math5-*Cre did not result in apparent morphological or functional abnormalities in RGCs, or in the retina. It has been shown that *Ezh2* mediates a feed-forward pathway contributing to tissue homeostasis in adults; thus, it is tempting to speculate that RGCs of *Ezh2* knockout mice may exhibit different susceptibility to stress or injury. Using both ocular hypertension (glaucoma) and optic nerve crush injury models, however, we observed no significant difference of RGC loss between littermate control and mKO mice. These data suggest a cell-lineage specific functionality of *Ezh2*.

The cell-lineage specific effect of *Ezh2* is further supported by gene expression profiling studies. The global changes in RGC gene expression induced in the absence of *Ezh2* was analyzed using RNA-seq. In the previous report, we found much less differentially expressed genes in mKO mice using Affymetrix cDNA microarray [8]. This observation is in agreement with the other reports which compared the readouts of the two technologies [52]. Reassuringly, these two independent measures of transcript abundance are highly correlated. Due to the limitation of the array technology that measures only genes with corresponding probes, which in most cases are designed to cover a very small portion of the 3’-end of the gene, numerous differentially expressed genes were missing in the array. Many of these genes, especially those carrying novel alternatively spliced forms, are identifiable with RNA-seq. By re-evaluating mRNA expression in the control and mKO RGCs with RNA-seq, many more differentially expressed genes were revealed. Consistent with the previous findings using the microarray technology, GO and IPA analysis detected none of the genes that are directly related to RGC development, survival or function. Most up- and downregulated genes are not specifically related to the retina. Expression of transcription factors that are known to mediate retinal or RGC differentiation, such as *Pax6*, *Sox2*, and *Brn3a*, showed no significant difference as compared to littermate control or WT mice. We showed previously that the expression of *Six1* and its cofactor *Eya1* was detected in the embryonic neuroretina of WT mice but were downregulated in the postnatal life. Deletion of *Ezh2* driven by *Chx10-Cre* led to derepression of *Six1* and *Eya2* expression in postnatal photoreceptors, but this was not observed in RGCs of mKO mice. These results are consistent with the absence of the phenotype in mKO RGCs, and it further supports a cell lineage-specific mechanism of *Ezh2-*mediated gene expression.

Our findings suggest a previously uncharacterized mechanism of *Ezh2* regulation in gene expression. Histone methylation is thought to be gene- or loci-specific, and the removal of specific histone methyltransferase or demethylase activities is more likely to change methylation at the level of specific gene rather than at a global level. The present study revealed that the gene suppression-mediated by *Ezh2* may be cell type specific, as absence of *Ezh2* caused ~20 fold increase of *Six1* expression in other retinal cell types, specifically photoreceptors, but not RGCs despite of highly enriched *Ezh2* levels. Previous report by Iida *et*.*al* [17] and Zhang *et*.*al* [16] also reported drastic changes in the expression of *Six1* and genes relating to early RPCs in *Dkk3-Cre* or *Pax6*-α-*Cre* driven *Ezh2* deficiency in the retina. The difference between the phenotypes observed in mKO and those from the previous reports, in part, is likely due to all PRC, particularly photoreceptor progenitor, deletion of *Ezh2* as driven by *Chx10* [53], *Pax6*-α Cre [54] and *Dkk-Cre* [55]. *Math5* drives gene expression in RGC lineage cells, beginning at E11.5 [56], a comparable time as *Chx10* [53] and a day later than *Pax6*-α [54] and *Dkk* [55]. The different phenotypes observed between mKO and other mutant mice carrying retinal deletion of *Ezh2* cannot be explained by the compensatory effect of *Ezh1*, as the expression of *Ezh1* was not changed in mKO mice (not shown). Thus, these studies reveal a novel insight into *Ezh2-*mediated gene suppression.

At this stage, we cannot completely rule out the possibility that the lack of defects in RGCs of mKO mice may be a result of non-cell autonomous effects from the wild-type cells. It has been reported that only 3% of total retinal cells, including 55% of RGCs, are labeled positive for transgene expression in adult *Math5*-Cre mice [27]. The fact that such a small percentage of non-RGC retinal cells are derived from *Math5*^*+*^ progenitors offers an explanation to why no phenotype is observed in other retinal cell types of mKO mice. On the other hand, because *Math5-Cre* drives *Ezh2* deletion in only 55% of RGCs, RNA-seq is much less effective in detecting gene expression changes in *Ezh2* deficient RGCs, especially those that were down-regulated. Just as it was shown for *Ezh2* itself, the biggest detectable change for the down-regulated genes theoretically is only 2 fold; thus the number of genes and their fold changes detected likely are truncated. In the present study, we have also applied RGC count, a sensitive method which can detect a less than 10% RGC loss. By quantifying RGCs under both the normal and diseased/injury conditions, nevertheless, we found no significant differences between WT and mKO mice, strongly suggesting that the 50% of *Ezh2-*deficient RGCs in mKO mice behaved similarly to WT cells.

In summary, our results indicate that despite the high levels of *Ezh2* expression in RGCs during early development, selective deletion of *Ezh2* from *Math5*^+^ progenitors does not affect RGC maturation and function, nor does it alter their injury responses and survival in the adult. These data suggest that *Ezh2*-mediated gene repression is not required for stabilizing RGC homeostasis. While the cell lineage specific mechanism of *Ezh2* remains to be elucidated, it is tempting to speculate that the variable composition of the polycomb repressive complex (PRC) which is required for *Ezh2* function, may contribute to the cell-specific fine-tuning during development [57]. Further exploration of the roles for *Ezh2* in specific cell lineages will be beneficial for future development of epigenetic therapies for injuries and diseases.

## Supporting information

S1 Fig The level of *Cre* mRNA in WT and mKO retina. Results of qPCR detecting *Cre* mRNA levels in E16 retinas and purified RGCs of P0 mouse pups of WT (white bar) and mKO (black bar) mice. Note the high levels of Cre expression were detected only in mKO retina or RGCs (n > 3/group).
(TIF)

S2 Fig Normal retinal morphology in mKO mice. Retina sections of 12-month-old WT (Control) and mKO mice that were double-immunolabeled for RGC marker β-III-tubulin (red) and photoreceptor marker Recoverin (green) and counterstained with DAPI (blue). Note the normal retinal laminar structure, morphology, and comparable immunolabeling intensity in retinal sections of both control and mKO mice. Scale bar: 100 μm.
(TIF)

S1 Table Gene Ontology analysis for differentially expressed genes in WT and mKO mice in numerical form. (PDF)
