# Supplementary figures and images for: Ezh2 does not mediate retinal ganglion cell homeostasis or their susceptibility to injury
Source: PLoS One. 2018 Feb 6;13(2):e0191853. doi: 10.1371/journal.pone.0191853 (PMC5800601; doi:10.1371/journal.pone.0191853)

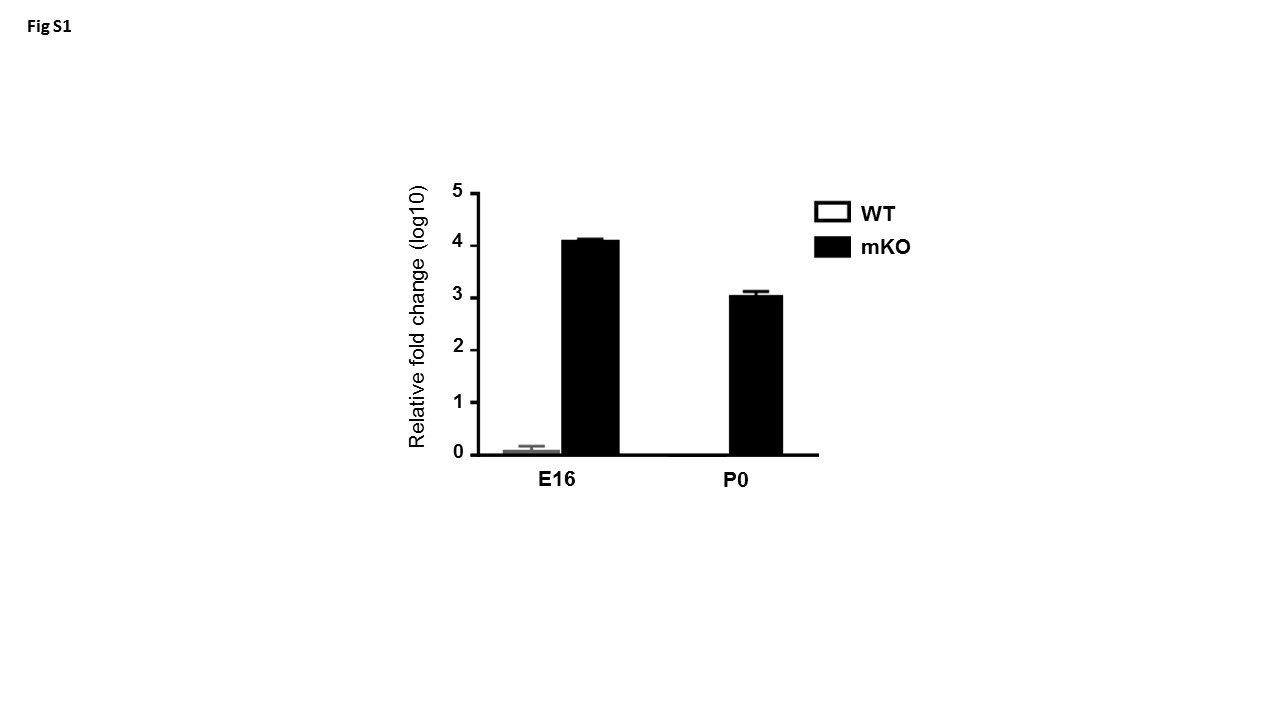

Supplement: S1 Fig — Results of qPCR detecting Cre mRNA levels in E16 retinas and purified RGCs of P0 mouse pups of WT (white bar) and mKO (black bar) mice. Note the high levels of Cre expression were detected only in mKO retina or RGCs (n > 3/group). (TIF) [file pone.0191853.s001.TIF]

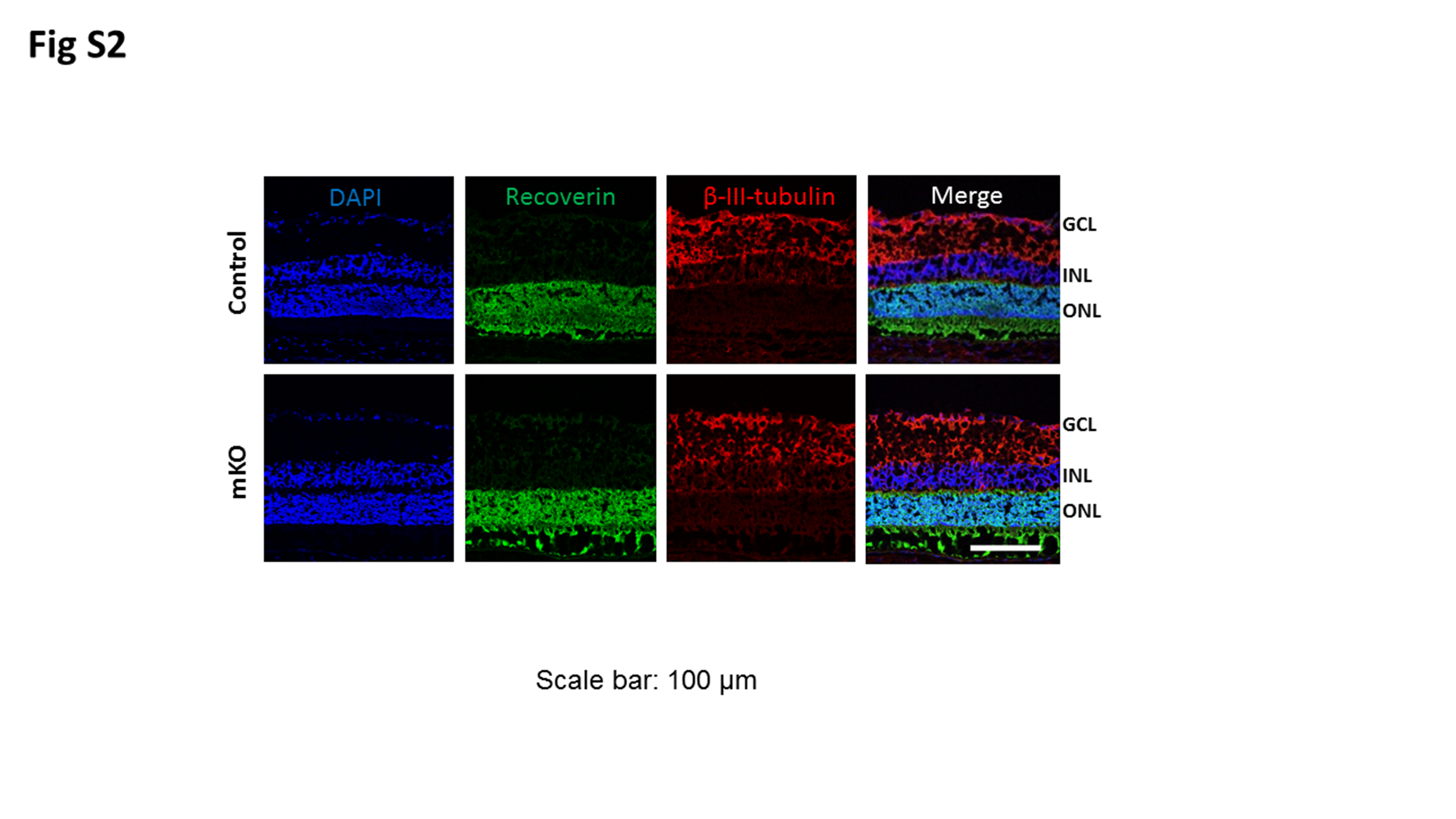

Supplement: S2 Fig — Retina sections of 12-month-old WT (Control) and mKO mice that were double-immunolabeled for RGC marker β-III-tubulin (red) and photoreceptor marker Recoverin (green) and counterstained with DAPI (blue). Note the normal retinal laminar structure, morphology, and comparable immunolabeling intensity in retinal sections of both control and mKO mice. Scale bar: 100 μm. (TIF) [file pone.0191853.s002.TIF]
