# Supplementary material for: Ezh2 does not mediate retinal ganglion cell homeostasis or their susceptibility to injury
Source: PLoS One. 2018 Feb 6;13(2):e0191853. doi: 10.1371/journal.pone.0191853 (PMC5800601; doi:10.1371/journal.pone.0191853)
Supplement: S1 Table — (PDF) [file pone.0191853.s003.pdf]

Gene Symbol	mKO RGC reads	WT RGC reads	FC mKO vs. WT	pva	padj
Gfap	264.4068341	37.30473667	7.087755005	0.255924407	0.651068942
Zbtb20	1710.131804	413.294537	4.137804039	4.19E-12	3.70E-09
Cdr1	196.4022366	48.88584202	4.017568861	2.66E-06	0.000119255
Serpina3n	108.8361709	27.43265676	3.967394475	0.003289567	0.02934931
Slc16a4	56.23565402	15.96287202	3.522903268	0.018993146	0.109364226
Irs4	55.13261435	15.73357942	3.50413678	0.000332879	0.005012279
Muc6	45.15058144	13.23668078	3.411019892	0.167875479	0.504942131
Aph1c	116.0355932	34.80156801	3.334205894	4.10E-10	1.45E-07
Alas2	36.92737276	11.53591628	3.20107843	0.008214576	0.058625873
Hbb-bt	54.71770724	17.58328465	3.111916136	1.39E-05	0.000430733
Hmga1-rs1	90.90428121	30.61712259	2.969066768	1.28E-07	1.21E-05
Clic6	33.40176118	11.27321192	2.962932075	0.001042191	0.012141037
Gabra2	977.105324	330.1998263	2.959133368	3.66E-17	1.62E-13
Ucp1	32.24301682	10.91830763	2.95311489	0.005165629	0.041244149
Efcab5	102.332949	34.81930472	2.938971637	5.80E-08	6.63E-06
Ttn	114.4334291	39.44144932	2.901349497	0.024691149	0.133071242
PYURF	78.54693748	27.45077484	2.861374148	1.55E-06	7.80E-05
Zfp459	49.74034978	17.60729139	2.824985893	0.000109495	0.002099618
Onecut2	1316.027683	471.9589322	2.788436859	6.88E-12	5.07E-09
RP23-308M1.2	46.56082595	16.69914465	2.788216219	0.020055808	0.114022256
Vcp-rs	77.68628512	28.13726867	2.760974636	2.43E-06	0.000112001
Lrrc70	42.40322707	15.42982361	2.748134272	0.000480719	0.006694717
Zfp455	62.77845116	22.92916184	2.737930483	2.24E-05	0.000622659
Spata9	53.58487756	19.66678717	2.724638097	0.000105231	0.002026805
Slc6a16	44.81746131	16.461139	2.722622128	0.000387294	0.00563329
Mmrn1	84.88576911	31.38436395	2.704715292	2.09E-06	9.94E-05
RP24-68F22.6	41.98802645	15.76141315	2.663976006	0.000697064	0.00891623
Mterf	41.93108545	15.83656835	2.647738104	0.000695958	0.008911501
Col8a1	201.0919117	75.99560799	2.646099124	0.004695944	0.038313126
Zfp442	83.56935756	31.93514908	2.6168457	0.000297884	0.004603844
Pkhd1	36.51292677	13.99382512	2.609217025	0.001787191	0.018311107
Il1rapl1	445.2578596	171.238055	2.600227267	2.06E-13	3.03E-10
Hba-a2	43.37126703	16.82231331	2.578198742	0.000611006	0.008071201
Nts	67.82707943	26.32549418	2.576478868	3.46E-05	0.000872331
Nbeal1	1357.583075	527.1356976	2.575395826	1.11E-08	1.89E-06
Rtl1	63.8921335	24.89439604	2.566526755	0.150456646	0.47023909
Tlr3	28.39276984	11.14690824	2.547143048	0.006238265	0.047547548
Zfp937	71.58458271	28.37283129	2.52299751	3.03E-05	0.000782086
Wdfy1	1564.734251	620.9498852	2.519904244	5.19E-21	3.44E-17
Vmn2r1	32.51240806	12.91136126	2.518123954	0.004153029	0.035014854
Ccdc18	173.5813525	69.52727454	2.496593656	3.51E-09	8.58E-07
Tmprss11e	102.86928	41.21599935	2.495857959	2.03E-06	9.70E-05

Lrrtm4	166.5725524	67.2238137	2.477880132	1.14E-08	1.90E-06
Lrrc39	45.75849734	18.50847612	2.472299559	0.000838466	0.010292942
Xist	5570.583825	2255.96214	2.469271858	0.000803181	0.009951941
Col4a3	47.14837876	19.11844507	2.466119948	0.000695506	0.008911501
Lpar4	200.3476782	81.74654963	2.450839565	6.17E-09	1.26E-06
Nexn	31.623369	12.92527097	2.446631028	0.004553346	0.037426079
Zfp947	56.82209702	23.32620523	2.435976896	0.000308212	0.004721281
Zfp820	137.6585976	56.51988636	2.435578104	1.05E-07	1.05E-05
Lin28b	306.1425721	125.9006353	2.43162055	2.01E-07	1.68E-05
Hpgd	384.0187453	158.5929144	2.421411742	9.74E-13	1.12E-09
Zfp458	257.7274121	106.7303355	2.414753134	2.01E-10	7.94E-08
Hdx	347.6666276	144.5622319	2.404961676	1.26E-11	7.96E-09
Hnf4g	39.20564454	16.32404633	2.401711177	0.001880979	0.019016697
Neurod6	62.66672712	26.15362007	2.396101456	0.000174228	0.003031385
F3	25.41939003	10.62776954	2.391789729	0.012783384	0.081200378
Zfp229	514.7436889	215.566867	2.387860881	3.17E-14	7.15E-11
Adam7	60.72989257	25.48103596	2.383336874	0.000216306	0.003562295
Cd46	45.03732758	18.9277609	2.379432402	0.001291658	0.014294489
Zfp758	318.8671618	134.1713947	2.376565903	1.66E-11	9.98E-09
Gpr101	41.98695776	17.70087528	2.372027207	0.004975559	0.040047615
Znf41-ps	41.25282038	17.45315399	2.363631261	0.002091485	0.020646992
Ell2	94.92023955	40.33116736	2.353520757	1.01E-05	0.000331803
Zfp119b	128.7016921	54.79542574	2.34876708	7.29E-07	4.58E-05
Sh2d4b	66.28692629	28.33804985	2.339149188	0.000456161	0.006403161
Fancb	124.1458826	53.11866797	2.33714224	1.08E-06	5.93E-05
Nup62cl	24.83275885	10.63090455	2.33590272	0.016156052	0.096790299
Zmat1	325.7694343	139.6637696	2.332526433	3.88E-11	2.06E-08
Erbb4	184.2533619	79.0742605	2.330130699	1.98E-08	2.95E-06
Bche	58.14533758	24.95876225	2.329656295	0.000327362	0.004951697
Slc16a9	36.10543568	15.49870879	2.32957701	0.004797708	0.038939705
Nlrp10	29.88216231	12.91763128	2.313284972	0.011462306	0.074731869
Pmel	280.993662	121.828626	2.306466642	1.31E-05	0.000411488
Lrp2bp	28.27551476	12.28052058	2.302468741	0.013280772	0.083538667
Papolb	81.95803894	35.63455954	2.299959365	7.79E-05	0.00160523
Itgbl1	160.5793474	69.88387341	2.297802619	1.80E-07	1.54E-05
Fam46a	515.0361651	226.1253843	2.277657445	7.20E-13	9.47E-10
Col19a1	182.1367591	80.33107008	2.267326439	3.11E-05	0.000801021
Xlr3b	31.59280381	13.97156944	2.261220828	0.081586362	0.311900805
Tdrp	72.57269225	32.13485691	2.258379194	0.000211582	0.003515238
Dbpht2	319.0678808	141.4692883	2.255386201	3.10E-10	1.14E-07
Smc1b	23.74036185	10.55574935	2.249045621	0.025529671	0.136343355
Mcf2	64.22248751	28.55724544	2.24890344	0.000371061	0.005447981
Nkapl	25.96697129	11.55992301	2.246292754	0.019553214	0.111860417

Kcnh7	1533.292179	683.2619059	2.244076782	3.66E-05	0.000911458
Arsk	158.3675494	70.65080415	2.241553388	3.88E-07	2.78E-05
Tpm3-rs7	25.31152075	11.30035366	2.239887487	0.021742825	0.121349767
Pmaip1	63.58557674	28.53185474	2.228581959	0.054984147	0.235572734
Ccdc176	99.84773742	44.86914739	2.225309444	2.97E-05	0.000773848
Rbm4	191.2494674	85.96196378	2.22481501	8.08E-08	8.40E-06
Zxdb	238.5081874	107.291599	2.222990333	1.09E-08	1.87E-06
Ermn	138.554571	62.74714991	2.208141266	8.71E-05	0.001747445
Prss41	44.25890276	20.06520023	2.205754353	0.003846837	0.033042672
Cers3	23.99077276	10.88106887	2.204817655	0.028116379	0.146441547
Wif1	36.3685207	16.56970597	2.19488027	0.029059191	0.150088333
Cdk15	54.04189361	24.67030011	2.190564905	0.001603717	0.016935184
Grem1	286.917126	131.3637925	2.184141615	4.01E-09	9.32E-07
Prex2	37.60178307	17.24229008	2.180788218	0.011054709	0.072917079
Tnfrsf10b	46.61728527	21.39263175	2.179128113	0.003618648	0.031496539
Irf4	33.02420716	15.18799097	2.174363102	0.011423815	0.07459096
Hba-a1	53.10085758	24.47128426	2.169925249	0.001777279	0.018237742
Slc5a7	147.2539431	67.89258511	2.168925264	2.69E-06	0.000120038
F8	104.9896116	48.50241202	2.164626609	0.000550382	0.007411852
Nanos2	55.942946	25.87769798	2.16182081	0.001823694	0.018591725
Lrriq1	65.95102009	30.54265937	2.159308372	0.000655485	0.008490878
Slc3a1	25.96912923	12.061325	2.153090911	0.031805112	0.159796782
Six4	69.18055056	32.21314712	2.147587452	0.000565894	0.007572348
Has2	61.90068291	28.90068304	2.141841521	0.000762948	0.009565172
Nup210l	51.17990323	23.92186881	2.13946091	0.034498007	0.16986985
Slc26a7	22.63315335	10.62463453	2.130252414	0.036036193	0.175112431
Il12rb2	155.0477678	73.00603469	2.123766459	2.66E-06	0.000119199
Slc5a3	1356.208718	639.899054	2.119410413	9.70E-07	5.53E-05
Dynlt1-ps1	22.06797452	10.42004065	2.117839581	0.045321385	0.206115396
Rmst	199.568834	94.39660479	2.114152669	4.17E-07	2.89E-05
Hsbp1l1	30.87519531	14.62397382	2.111272606	0.01756419	0.103060868
Fst	250.5006307	118.6664302	2.110964578	5.94E-08	6.73E-06
Nox4	176.9984873	83.86241914	2.110581701	1.38E-06	7.20E-05
Pate2	27.36640612	13.0136582	2.102898793	0.027007438	0.142145541
Lrif1	712.646944	339.5569473	2.098755304	5.33E-12	4.40E-09
Zfp712	178.1465889	84.92193534	2.09776883	8.74E-07	5.18E-05
Cwf19l2	778.625408	371.924966	2.093501322	1.75E-12	1.86E-09
Zfp58	150.359096	71.8773084	2.091885455	3.77E-06	0.000156925
Ccdc79	77.27925515	36.94248901	2.091880033	0.000461889	0.006466444
Vwa3a	23.41402962	11.20119172	2.090315941	0.042327011	0.196282447
Chic1	482.0553443	231.0801918	2.086095483	2.60E-08	3.63E-06
Klhl10	24.54191528	11.7714789	2.08486253	0.040627226	0.191209002
Zfp759	166.3358843	79.92294137	2.081203237	2.80E-06	0.00012342

Slitrk4	380.5536771	183.5318076	2.073502583	9.36E-09	1.69E-06
Neat1	47.96828439	23.14698775	2.07233377	0.004753838	0.038666492
Gk5	189.0920119	91.33255398	2.070368162	1.19E-06	6.39E-05
Kcnrg	50.58757341	24.44727753	2.069251815	0.003759544	0.032450544
Cetn4	57.19826828	27.64596369	2.068955487	0.001850425	0.018784788
Serpinb12	30.25971633	14.69355098	2.059387576	0.024097313	0.130828082
AI504432	492.4837095	239.1835396	2.059020074	2.97E-10	1.11E-07
Fut9	863.4171746	419.4098594	2.058647777	6.05E-10	1.91E-07
Trim12c	31.41152634	15.25931917	2.058514274	0.01558037	0.094386356
Gramd1c	70.11773183	34.14114256	2.053760553	0.001119742	0.012831058
Hrc	36.53328986	17.78857052	2.053750739	0.015669718	0.09471125
Cntn3	184.6713486	89.92681795	2.053573703	1.19E-06	6.39E-05
Zfp942	411.1122401	200.2393404	2.053104247	1.11E-09	3.19E-07
Ankrd12	3585.567036	1746.912029	2.052517229	5.86E-15	1.73E-11
Edn1	27.3498978	13.35151776	2.048448595	0.032359765	0.161818824
Tchh	50.1442796	24.5130277	2.045617547	0.004451359	0.036804563
Dct	153.3598349	75.17553787	2.040023115	0.005789004	0.044870284
Zfhx4	2172.350477	1065.437015	2.038929046	2.34E-07	1.89E-05
Ftx	279.5933061	137.1310869	2.038876176	7.83E-05	0.001613152
Zfp160	817.4814864	401.0893702	2.038152958	9.45E-12	6.77E-09
Asgr1	20.40901593	10.04043764	2.032681906	0.075186842	0.294136074
Spata1	89.72655653	44.17079121	2.031354976	0.000287745	0.004469734
Vps13a	735.9043751	362.7296642	2.028795678	1.91E-11	1.10E-08
Sacs	2327.821046	1147.91173	2.027874604	1.48E-07	1.36E-05
H2-M5	115.671929	57.05991107	2.027201355	0.06870673	0.274744029
mt-Tc	23.16005746	11.43048432	2.026165891	0.056400247	0.239818626
Spdya	44.81283136	22.11880737	2.02600577	0.006827471	0.051039534
Ghrl	27.58457557	13.63822885	2.02259222	0.038051314	0.181802873
Tyrp1	63.23224058	31.2827447	2.021313704	0.014022128	0.086952932
Dnah5	64.91665333	32.12789491	2.020569773	0.003065753	0.027830025
Hbb-bs	114.6512839	56.77701268	2.019325753	5.61E-05	0.001248865
Vdac3-ps1	31.86112635	15.81256162	2.01492504	0.024348137	0.131677587
Vcan	1601.104047	795.9152963	2.011651308	7.54E-07	4.66E-05
Tmed5	579.0410291	287.8958551	2.011286439	1.17E-10	5.50E-08
Ano5	77.76574714	38.73164074	2.007809265	0.000962691	0.011426467
Flrt3	1075.961556	536.2200037	2.006567357	5.87E-12	4.45E-09
Rassf6	35.33966442	17.64903483	2.002356773	0.019167555	0.110129336
Mycbp	165.0021687	82.50263492	1.999962411	6.87E-06	0.000246782
Gbp7	56.50242619	28.30845079	1.99595614	0.058952092	0.247845653
Tmem27	93.81409974	47.03230979	1.994673452	0.000282417	0.004412826
Prkg2	42.04172025	21.1368894	1.989021159	0.013650296	0.085144966
Prrg1	98.65168109	49.70666058	1.984677303	0.000175968	0.003057648
Kcnt2	85.78685414	43.22748167	1.984544341	0.005632125	0.043986384

Pik3c2a	1323.876184	667.8813242	1.98220273	2.72E-12	2.57E-09
Ccdc73	266.5746044	134.6950524	1.979097226	3.30E-07	2.44E-05
Kbtbd8	272.4760696	137.9887486	1.974625268	1.79E-05	0.000524214
Cyp2r1	23.04697121	11.68691866	1.972031455	0.067579238	0.271957971
Eda2r	37.17638034	18.86024539	1.971150405	0.025288389	0.135272731
Zfp825	73.81783298	37.48425048	1.969302628	0.001043284	0.012143863
Eif2ak2	139.5332742	70.89400647	1.968195637	2.79E-05	0.000736891
Zfp932	224.6361235	114.2443462	1.966277816	9.08E-07	5.32E-05
Itga4	421.5203044	214.4495367	1.96559205	1.71E-07	1.49E-05
Katnbl1	377.0413267	191.937965	1.964391602	1.43E-08	2.23E-06
Cdhr2	38.61101033	19.66678717	1.963259682	0.018103068	0.105433895
Prorsd1	131.9229055	67.19705333	1.963224561	4.61E-05	0.001086637
Acvr1c	46.68224986	23.82584189	1.959311662	0.008729143	0.061379462
Zc3h6	486.6546892	248.9895444	1.954518573	3.24E-09	8.04E-07
Lmod1	45.35454696	23.21067627	1.954038152	0.02925655	0.150796651
Swt1	432.566433	221.4441123	1.953388728	7.73E-09	1.48E-06
Ccdc173	127.3216988	65.22417943	1.952062868	6.39E-05	0.001378199
Scai	2021.894426	1036.705961	1.95030655	1.63E-12	1.80E-09
Zfp709	184.5097855	94.87918242	1.944681444	6.64E-06	0.000241983
Ptgs2	155.8325194	80.13732166	1.9445686	2.27E-05	0.000629832
Ago3	862.5547412	444.389479	1.940988214	0.000871817	0.010597778
Cdh9	25.64172831	13.21580906	1.940231445	0.063478444	0.260435466
Atg4a	147.6817973	76.21519923	1.937694827	3.49E-05	0.000878258
Eif4e3	231.5387512	119.6093583	1.935791266	1.90E-06	9.20E-05
Serpine3	47.98125201	24.79973879	1.934748281	0.00965216	0.065912096
Foxp2	1286.211177	664.8997155	1.934443867	2.23E-11	1.23E-08
Efhc2	161.0321946	83.25177249	1.934279473	0.000643993	0.008371134
Zfp654	874.1900771	452.0169713	1.933976228	1.55E-10	6.51E-08
Zfp51	244.1690475	126.5471225	1.929471352	1.17E-06	6.30E-05
Zfp53	140.4085705	72.86688036	1.926918921	4.74E-05	0.001108147
Pls1	40.50850145	21.03527015	1.925741917	0.016397699	0.098016544
Efcab7	109.7174387	57.01326728	1.924419419	0.000233142	0.003767216
Rln1	27.38538645	14.23183077	1.92423497	0.044800292	0.204391699
Slc23a1	57.35472033	29.81579178	1.923635661	0.054930898	0.235458728
Birc3	19.33235206	10.05190433	1.923252692	0.097223291	0.351919651
Hist2h2ac	21.34401869	11.10133781	1.922652842	0.099345406	0.357185461
C77370	1650.823281	860.0396708	1.919473412	1.22E-11	7.91E-09
Nt5dc1	128.1035172	66.77951959	1.918305463	6.65E-05	0.001418397
Fam46b	22.50462748	11.74364517	1.916323863	0.067943216	0.272884326
Pcsk1	256.2960704	133.7625318	1.916052776	0.000290933	0.004511335
Amy1	147.3696484	76.94175622	1.915340326	0.008084604	0.058063748
Prrg4	22.65598845	11.83967209	1.913565534	0.061648527	0.255226978
Zfp943	388.3222795	202.9944387	1.91297004	4.78E-08	5.71E-06

Adam21	22.79547106	11.9217893	1.912084712	0.073358571	0.288558947
Cybrd1	28.22768662	14.79271291	1.908215672	0.098668733	0.355713205
Rgs1	25.60376764	13.42598098	1.907031425	0.069347874	0.276473949
Pcdh20	227.5442452	119.3835114	1.905993907	5.66E-06	0.000213305
Fign	673.170065	353.2490218	1.905653019	0.000250439	0.003997984
Zfp125	63.41599006	33.28276033	1.905370511	0.003164492	0.028530999
Rgs17	1313.765249	689.60676	1.905093344	1.10E-05	0.000355189
Shcbp1l	25.52445267	13.40335822	1.90433265	0.23473412	0.619817757
Tex15	123.7223649	65.0254742	1.90267532	0.000155135	0.00277944
Ildr1	20.75602531	10.91448063	1.901696106	0.081831215	0.312384491
Yod1	289.4507527	152.2867395	1.900695712	5.76E-07	3.79E-05
Dach2	245.0671788	128.9361318	1.900686606	1.44E-06	7.45E-05
Zfp934	24.3311413	12.80210231	1.900558261	0.061691591	0.255281176
Klhl15	626.5946356	330.4117207	1.896405595	3.58E-09	8.58E-07
Parp14	31.33036748	16.52688918	1.895720795	0.048708536	0.216813086
Dnah7b	174.6459916	92.12799614	1.895688595	0.054567262	0.234413727
Cox17	99.80286296	52.66529373	1.89504047	0.00064165	0.008352469
Dennd1b	873.0006417	460.8103007	1.894490293	4.20E-10	1.45E-07
Kcnb2	529.2798968	279.4040783	1.894317005	1.26E-08	2.03E-06
Dleu2	66.87031027	35.30364769	1.894147337	0.003015464	0.027524279
Esf1	1051.662279	555.471036	1.893280136	1.21E-10	5.54E-08
Mybpc1	40.38028965	21.33069428	1.893060259	0.016261424	0.097267747
Cmbl	36.15774672	19.10590503	1.892490655	0.025254662	0.135119573
Zfp708	135.7529153	71.76491445	1.891633486	7.76E-05	0.001601834
Epha6	549.6347373	290.5774506	1.891525774	1.21E-08	1.97E-06
Tceal7	29.97272752	15.86057508	1.889762973	0.042900592	0.197972866
Zfp963	138.7003804	73.4023861	1.889589532	9.93E-05	0.001939174
Csgalnact1	146.9834626	77.83003383	1.88851855	7.90E-05	0.001626032
Nsun7	158.3616838	83.8666275	1.888256253	0.003006311	0.02745964
Zfp605	600.7768674	318.3462724	1.887180468	5.69E-09	1.20E-06
Kcnj3	173.0178705	91.77721517	1.885194165	2.64E-05	0.000706706
AI606181	245.0578136	129.9977383	1.885092901	2.49E-06	0.000113556
Proca1	34.75104292	18.43714791	1.884838322	0.187852863	0.540201925
Fam126b	1179.533274	626.1735534	1.883716212	1.43E-10	6.32E-08
Ovgp1	88.45007542	46.98781271	1.882404613	0.0166489	0.099118226
Trpm6	21.21056935	11.27948194	1.880455988	0.086565168	0.324157887
Chpt1	390.3927927	207.6795798	1.879784199	8.32E-08	8.58E-06
Lrrcc1	1181.571528	629.2624587	1.877708596	1.72E-10	7.00E-08
Fcer2a	34.84516939	18.5843233	1.874976496	0.060836435	0.253007728
Slc30a1	583.1627193	311.0501155	1.87481917	5.53E-09	1.18E-06
Mtm1	183.1873612	97.78008646	1.873462868	2.18E-05	0.000610684
Lipo1	184.9049576	98.76299274	1.872208937	1.62E-05	0.00048663
Zfp595	117.5344126	62.91552193	1.868130613	0.000309588	0.004734164

Cd274	49.31218155	26.3992797	1.867936629	0.014010729	0.086902573
Zfp71-rs1	448.6496661	240.400399	1.866260073	4.81E-08	5.71E-06
Zfp850	183.5476317	98.42100986	1.864923272	1.84E-05	0.000535453
Prokr2	152.3746379	81.7510829	1.863885254	8.73E-05	0.001749203
Tyw5	222.1811425	119.2158313	1.863688237	7.26E-06	0.000258606
H2-M6-ps	43.01654811	23.11220632	1.861204747	0.024437266	0.132078793
Haus3	329.7270689	177.1640622	1.861139697	3.55E-07	2.59E-05
RP24-113D21.1	219.9334154	118.1764949	1.861058882	9.23E-06	0.000308461
Scarna10	25.2795728	13.58883142	1.860319847	0.348402696	0.771201684
Zbtb26	587.5589978	315.9283416	1.859785655	1.35E-08	2.15E-06
Zfp760	552.7305891	297.2675356	1.859370846	1.20E-08	1.97E-06
Xrra1	35.60243537	19.15774548	1.858383357	0.037809756	0.181000811
Depdc1a	99.07211923	53.3932061	1.855519203	0.029408926	0.151414741
Gdap10	92.57576747	49.92342743	1.854355204	0.093519897	0.342132117
Zfp120	360.6306715	194.8611248	1.850706096	3.08E-07	2.32E-05
AU041133	57.50501261	31.08442084	1.849962491	0.006868294	0.051262078
Rasef	54.64149241	29.53848572	1.849840677	0.009017154	0.062772083
BB031773	43.05126157	23.34217661	1.844355062	0.018103806	0.105433895
Ptchd4	303.1775094	164.4380612	1.843718584	0.000318295	0.004836625
Rpl14-ps1	52.85706696	28.7409676	1.839084463	0.010263114	0.069000188
Tpt1-ps3	39.42551081	21.44308824	1.838611601	0.024800614	0.133417
Zfp300	131.6821945	71.63861077	1.838145562	0.000187943	0.00321628
Lrrc6	41.87523371	22.78894845	1.837523736	0.025842987	0.137490499
Zfp935	120.4562565	65.55646096	1.837442943	0.000352035	0.005235305
AI429214	101.454385	55.265859	1.835751526	0.000798416	0.009902146
Pla2g4c	58.38988283	31.82658212	1.834626245	0.024258809	0.131435752
Zfp182	231.1661412	126.0788216	1.833504932	6.84E-06	0.000246167
Zfp597	572.0756358	312.0261026	1.833422368	3.11E-08	4.18E-06
Cep290	800.8459598	436.8140959	1.833379388	1.47E-07	1.36E-05
Col10a1	42.53560771	23.23920197	1.83033857	0.028908778	0.149569787
Zfp518a	551.6086102	301.7115361	1.828264896	2.34E-08	3.41E-06
Rab27b	466.3097984	255.1318242	1.827721022	1.78E-07	1.53E-05
Krt20	59.03435607	32.30534705	1.827386531	0.007949307	0.057327472
Zfp273	138.0730231	75.71135422	1.823676574	0.000213906	0.003538325
Ankrd61	43.68988972	23.98869233	1.821270168	0.020274557	0.114910316
Rwdd3	82.25552336	45.16625182	1.821172226	0.002768739	0.025732873
Vsig2	19.67904738	10.80591367	1.821136832	0.120838942	0.408277955
Ubn2	2572.064867	1412.527277	1.820895717	4.14E-10	1.45E-07
Gucy1a2	599.5008268	329.304883	1.820503909	5.08E-08	5.94E-06
Ccdc150	26.06570715	14.32266102	1.819892763	0.167792261	0.504785522
Uggt2	831.8213772	457.3552796	1.81876413	0.000403182	0.005807048
Tfpi	66.10981765	36.34956479	1.818723774	0.013396684	0.084077269
Nfat5	1849.157445	1016.942003	1.818350938	0.000512195	0.007029697

Zfp429	51.19595043	28.18704747	1.816293476	0.01357256	0.084839701
Mgat4c	633.667857	349.5872029	1.812617429	3.69E-08	4.72E-06
Cdh12	478.2485274	264.1906402	1.810240238	8.51E-06	0.000291099
Sass6	418.7321828	231.3147375	1.810226997	2.59E-07	2.04E-05
AI607873	32.22992271	17.80767691	1.809889233	0.508475626	0.926226179
Akap17b	805.6064247	445.2176696	1.809466424	1.41E-08	2.21E-06
Cenpk	127.3044152	70.38602384	1.808660417	0.001395087	0.015199503
Zfp87	435.7037009	240.9817857	1.808035821	3.25E-07	2.41E-05
Glmn	403.3813691	223.1987932	1.80727397	4.85E-07	3.29E-05
Rpl10a-ps1	27.58241762	15.26941619	1.806383249	0.065260106	0.265852968
Zfp72	80.93230335	44.82319559	1.805589769	0.00326286	0.029189608
Trmt13	186.8573789	103.5149717	1.805124185	5.07E-05	0.001160094
Vax2os	161.4481498	89.53789497	1.803126485	0.005062684	0.040642482
Zfp945	884.2403661	490.39961	1.803101691	5.55E-09	1.18E-06
Col28a1	63.64186904	35.29737767	1.80301975	0.006995746	0.051989683
Bicd1	1825.779349	1012.759648	1.802776555	6.21E-09	1.26E-06
Rel	106.1687191	58.89394125	1.802710378	0.004563848	0.037470093
Bace2	20.09703408	11.15523992	1.801577933	0.303669588	0.717144888
Tph2	32.14040563	17.84598902	1.800987639	0.05538707	0.236954429
Zfp599	217.7830208	121.0074261	1.799749221	2.31E-05	0.000635572
Ankrd32	689.3529761	383.6644028	1.796760323	2.62E-08	3.64E-06
Lcorl	1550.732361	863.3645337	1.796150178	7.45E-10	2.30E-07
Gnpnat1	169.7062801	94.48811273	1.79605958	9.18E-05	0.001823072
Nebl	351.3238652	195.756675	1.794696734	4.17E-05	0.001007653
Scn9a	2493.334999	1389.393366	1.794549376	1.89E-06	9.18E-05
Glra3	35.89575096	20.00778174	1.794089491	0.045426406	0.206431292
Diap2	278.6010271	155.3583592	1.793279927	6.18E-06	0.000228343
Tdrd5	45.63906376	25.49495996	1.790121021	0.092107948	0.33850788
Trf	24.10387956	13.4684164	1.789659514	0.085379742	0.321124716
Caap1	272.5951274	152.3827521	1.788884396	5.27E-06	0.000203857
Brwd3	928.0235223	518.8764328	1.788525097	7.84E-09	1.48E-06
Ncmap	39.81404212	22.26423172	1.788251336	0.090632277	0.334877644
Zfp874b	271.6862275	152.026803	1.787094263	1.02E-05	0.000334628
Zfp959	257.2833637	143.9773431	1.786971187	1.04E-05	0.000339967
Mme	102.6254894	57.45931244	1.786054949	0.001461956	0.015745418
Ccne2	316.7648478	177.5496246	1.784091904	5.65E-05	0.001256131
Npat	860.9834783	482.7830768	1.783375432	1.21E-08	1.97E-06
Zfp82	22.3736296	12.54949496	1.782831075	0.149715287	0.468695461
Kctd4	60.58871314	34.00091488	1.781973025	0.010254383	0.068958973
Ankef1	36.52480513	20.52936347	1.779149421	0.040741688	0.191533446
Zfp951	22.36899964	12.57350169	1.779058864	0.110557759	0.384163222
Polr3g	136.8945438	76.9688272	1.778571257	0.000389249	0.005652423
Fbxw10	19.77424255	11.1197665	1.778296563	0.140021685	0.449820086

Trpm1	120.5556411	67.80213623	1.778050778	0.000757887	0.009510712
Lyrm1	90.83686517	51.1200363	1.77693272	0.00255148	0.024136653
Ccdc148	198.5001937	111.7439455	1.77638433	7.41E-05	0.001540847
Jrkl	383.2803126	215.8713148	1.775503674	1.14E-06	6.18E-05
Zfp748	536.7993651	302.338889	1.775488978	1.70E-07	1.49E-05
Zfp944	303.277481	170.9176923	1.774406599	4.26E-06	0.000173629
Wnk3	1483.94788	836.6087963	1.773765572	2.97E-09	7.51E-07
Slc7a2	188.1597747	106.0964163	1.773479079	7.99E-05	0.001639437
Cacnb4	927.8552979	523.1857987	1.773471872	1.01E-06	5.65E-05
Dpy19l4	910.9657132	513.6739891	1.773431656	1.33E-08	2.12E-06
Lrp1b	795.2967154	448.5622312	1.772990814	0.02571673	0.13698369
St8sia4	858.7795569	484.4639722	1.77263864	1.89E-08	2.83E-06
Skor1	26.23219369	14.80525295	1.77181665	0.087874448	0.327546736
Kcne2	27.83391778	15.70957269	1.771780705	0.068884987	0.27508348
Kbtbd3	172.7938354	97.63358876	1.769819563	0.00011313	0.002153445
Steap2	753.4116167	425.902256	1.768977755	5.52E-08	6.41E-06
Zfp931	73.894823	41.77863253	1.768722874	0.005265561	0.041840457
Dcdc5	910.4468339	515.8723007	1.764868617	4.24E-05	0.001017404
Zfp85-rs1	109.2450036	61.96180476	1.76310235	0.001103454	0.012688289
Clec1a	22.19480357	12.60064343	1.761402399	0.120114121	0.406771783
Zfp329	1237.510032	702.5858062	1.761364977	8.61E-09	1.58E-06
Cage1	112.5644711	63.90892088	1.761326425	0.001395791	0.015199503
Klhl32	867.7380565	492.9980579	1.760124695	5.52E-06	0.00020948
Olfm3	138.8616294	78.90134162	1.759940029	0.005268732	0.041840583
Bod1l	3201.127539	1819.11035	1.75972147	7.70E-10	2.35E-07
Slc4a5	176.7824758	100.4766929	1.759437642	0.000151736	0.002727755
Cldn1	102.2917822	58.14580627	1.75922889	0.009905599	0.067244458
Hfm1	400.5638774	227.7635337	1.7586831	6.53E-05	0.001397623
Atr	1110.463276	631.4527486	1.758584912	9.84E-09	1.76E-06
Zfp958	167.1272562	95.16696687	1.756147765	0.000171397	0.002997862
Zfp933	278.2267203	158.4927213	1.755454244	6.98E-05	0.001470497
Ube2v2	436.2176373	248.5138437	1.75530518	1.04E-06	5.75E-05
Triqk	410.5399387	234.0878832	1.753785515	1.28E-06	6.74E-05
Atp11c	1474.598066	841.5124278	1.752318822	4.29E-09	9.73E-07
Cd55	179.8836062	102.6916958	1.751686004	0.000141408	0.002578793
Zfp433	33.90197541	19.35537736	1.751553316	0.049446463	0.219068071
Zfp949	667.2443608	381.3481199	1.749698834	1.84E-07	1.56E-05
AK010878	325.0138446	185.801546	1.749252638	5.87E-06	0.000219293
Fam122b	594.0603995	339.7737563	1.748399894	2.42E-07	1.94E-05
Zfp449	193.3454135	110.6538276	1.747299824	0.000105214	0.002026805
Wdr52	31.07469849	17.78543551	1.747199188	0.076968776	0.298588327
Fkbp7	105.565559	60.44124604	1.746581448	0.001619211	0.017045382
Klhl4	221.7978899	127.0252097	1.746093475	5.49E-05	0.001227569

Zfp964	42.98908305	24.62786469	1.745546502	0.031242275	0.157644182
Styk1	109.7877879	62.90474722	1.745302107	0.001962487	0.01962082
Msr1	29.55786153	16.95137063	1.743685639	0.546689541	0.95942947
Tbc1d32	350.3542543	201.0438205	1.742676067	4.34E-06	0.000176317
Pcdh11x	1651.87556	948.0468114	1.742398729	5.36E-05	0.001206274
Etohi1	98.96751771	56.81738645	1.741852695	0.002243554	0.021855279
Orc4	985.3425658	565.9363794	1.74108363	3.30E-08	4.33E-06
Slc6a2	64.68691958	37.16275795	1.740638294	0.010950749	0.072442124
Ccdc162	18.89400228	10.86019715	1.739747632	0.158672827	0.486907612
Prlr	36.13954158	20.7826628	1.73892739	0.054335615	0.233852162
Ap1s3	109.1087476	62.78471357	1.737823451	0.002113119	0.02080634
Lztfl1	721.6947082	415.2912913	1.737803617	1.29E-07	1.22E-05
Pura	1599.715988	921.859539	1.735314243	7.25E-09	1.43E-06
Cdc14b	199.166601	114.830619	1.734438102	0.000109538	0.002099618
Pirb	21.42410885	12.35254077	1.734388839	0.125003795	0.418060543
Pkd1l3	38.76484331	22.35819698	1.733809007	0.05068304	0.222822003
Acer3	244.9133458	141.2723199	1.733625852	3.40E-05	0.000862714
Mettl20	101.566109	58.58634415	1.733614044	0.002034953	0.02020929
Nkain3	735.6417101	424.5639647	1.73269936	0.000170553	0.002989009
G2e3	1202.290282	694.0047097	1.732394991	1.50E-08	2.33E-06
Mastl	277.0108673	159.9040354	1.732356952	1.31E-05	0.000410256
Zfp81	1344.454935	776.4244367	1.731597914	1.59E-08	2.44E-06
Ptx3	37.95480516	21.92498819	1.731120895	0.039882642	0.188608549
RP24-312B12.1	412.4513197	238.3718154	1.7302856	3.51E-06	0.000148581
Far1	1579.89392	913.0880563	1.73027553	8.73E-09	1.60E-06
Sgms2	78.49632326	45.38231239	1.729667774	0.017126787	0.101098372
Pih1d2	36.02781753	20.84565933	1.728312689	0.050518063	0.222366693
Uba6	917.7696673	531.0919238	1.728080632	7.30E-08	7.87E-06
Zfp607	34.26239298	19.83277262	1.727564453	0.063980614	0.262119702
Rag1	131.6783397	76.24164899	1.727118202	0.000720556	0.009150514
Magt1	332.5944645	192.6022175	1.726846496	6.00E-06	0.000223236
Slfn2	52.13097374	30.23331123	1.724289257	0.041718605	0.194447271
Murc	165.6116349	96.05003352	1.724222563	0.000306958	0.004712975
Cspp1	852.1735369	494.2793113	1.724072842	1.81E-05	0.000529632
Spopl	646.013343	374.8274652	1.723495215	3.21E-07	2.38E-05
Ttc39b	420.5476551	244.0412611	1.723264554	0.000862348	0.010512892
Ppm1k	525.4331905	305.00238	1.722718329	8.09E-07	4.91E-05
Zdhhc20	1223.123656	710.8319233	1.720693199	2.35E-08	3.41E-06
Pdgfd	30.76766066	17.88459745	1.720344042	0.077625715	0.300309812
Zfp946	556.5379314	323.589542	1.719888498	7.26E-07	4.58E-05
Hook3	2050.97424	1192.599797	1.719750619	7.75E-09	1.48E-06
Calb1	238.3299895	138.6428475	1.719021167	5.07E-05	0.001160094
Zfp119a	126.5647263	73.63029473	1.718921902	0.001070269	0.012387273

Chrna5	162.325184	94.43452123	1.718917848	0.000365684	0.005392929
Zfp677	104.7843687	60.97361677	1.718519816	0.002671774	0.025060045
Foxp3	23.47868015	13.66223558	1.718509391	0.12766281	0.424567731
Slc25a32	244.6371872	142.3569304	1.718477537	4.00E-05	0.000973802
Asb5	28.62883036	16.65946287	1.718472593	0.087350344	0.32640667
Mum1l1	269.4541718	156.8608563	1.71779103	2.14E-05	0.000603577
Ccdc37	23.65888894	13.78853926	1.715837225	0.158466059	0.486546012
Col11a1	374.2360897	218.1493993	1.715503646	0.000644342	0.008371134
Prkdc	775.3779023	452.3185519	1.714229715	3.05E-07	2.30E-05
Nrg4	50.24133921	29.35162854	1.711705336	0.143348087	0.455553553
Fam199x	1014.66351	593.1556156	1.710619411	7.39E-08	7.90E-06
Samsn1	167.2024023	97.7466747	1.7105687	0.000337023	0.005060308
Zkscan4	142.5642026	83.36834623	1.710051945	0.000611215	0.008071201
Arr3	27.16552018	15.89712185	1.708832607	0.101893231	0.36285266
Zfp101	437.0205324	255.7961753	1.708471723	8.67E-05	0.001741887
Clec18a	112.4029081	65.84493739	1.707085048	0.002270342	0.021989645
Hist3h2a	251.6266519	147.4327742	1.706721272	5.60E-05	0.001247875
Ptprq	28.43952929	16.66642487	1.706396513	0.101579218	0.362074815
Zfp239	362.4070528	212.3837852	1.706378161	9.07E-06	0.000305262
Itgb3bp	180.2644075	105.6482387	1.706269879	0.000201854	0.003387562
Vps37a	1163.004988	681.7905891	1.705809683	5.78E-08	6.63E-06
Cngb3	452.7265261	265.4134447	1.705740742	2.35E-06	0.000109546
Lcor	1100.073961	644.990008	1.705567447	7.83E-08	8.19E-06
Zbtb37	561.9470806	329.5434813	1.705228938	0.005147941	0.0411506
Abca12	40.07540976	23.50258403	1.70514909	0.046626003	0.21008243
Map3k2	1576.078173	924.3655042	1.705037851	4.90E-05	0.001134731
Zfp770	650.5563519	381.5728649	1.704933479	4.06E-07	2.88E-05
Mir17hg	153.8127881	90.21850015	1.704891877	0.040095062	0.189275531
Rfesd	346.5848521	203.3242772	1.704591586	1.10E-05	0.000355189
Kcnmb2	86.64101211	50.84028722	1.704180225	0.005988748	0.046108491
Fastkd2	379.0859688	222.5373652	1.703471093	6.41E-06	0.000234575
BB218582	52.37273292	30.7597933	1.702636049	0.058000167	0.244698876
Phf11c	33.70494424	19.79936086	1.702324862	0.070077036	0.278877786
Sypl	580.0814497	340.7869393	1.702182163	7.51E-07	4.65E-05
Gda	20.59586555	12.10306844	1.701706114	0.1642049	0.498061899
Scube2	58.2531863	34.23334249	1.701650556	0.019343698	0.110853144
Mbnl3	40.06661097	23.56244554	1.700443653	0.040518673	0.190893455
Rmdn1	188.0617729	110.6201052	1.700068649	0.000219008	0.003586911
Zfp930	88.8356736	52.29890846	1.698614296	0.004620842	0.037814748
Ugt8a	248.7128521	146.465825	1.698094775	5.02E-05	0.001153923
Chm	977.22331	575.6423888	1.69762222	1.24E-07	1.19E-05
Dmxl1	2241.640989	1320.553771	1.697500729	1.14E-08	1.90E-06
Zdbf2	2371.240373	1397.313514	1.696999527	0.000522381	0.007136253

Slitrk2	567.1998003	334.4358573	1.695989793	4.80E-06	0.0001904
Tmem245	1490.845961	879.8008467	1.694526627	6.51E-07	4.16E-05
Unc5c	1953.948289	1153.19337	1.694380439	0.000116785	0.00221204
Gnmt	35.40154943	20.90063479	1.693802594	0.068036124	0.273091108
Zmym1	410.579952	242.4556864	1.693422654	5.37E-06	0.000205737
Zfp938	297.4471807	175.6504655	1.693403885	2.14E-05	0.000604186
Cd59a	227.1747148	134.1915744	1.692913402	9.44E-05	0.001861085
Zic3	234.854637	138.7560042	1.692572788	8.78E-05	0.001757484
Map3k15	42.01687426	24.82932356	1.692227908	0.102022344	0.363117379
B3galt2	935.7242481	553.3141015	1.691126696	2.14E-07	1.75E-05
Kcnh8	272.5509871	161.1722974	1.691053559	4.23E-05	0.001017404
Duxbl1	34.78437362	20.57668495	1.690475104	0.061172505	0.254000962
Pgap1	1695.898338	1003.375469	1.690193143	3.45E-08	4.46E-06
Armcx5	319.0842215	188.8739142	1.68940334	1.61E-05	0.000483363
Pqlc3	37.71178973	22.34182994	1.687945429	0.059894223	0.250381016
Trove2	3225.300906	1911.523319	1.687293518	7.24E-09	1.43E-06
Gpr22	571.1651444	338.6943145	1.686373582	5.85E-05	0.001288475
Cdh7	459.460844	272.5769714	1.685618714	0.000637906	0.008320081
Hypk	77.24514926	45.8322553	1.685388353	0.008483433	0.060049845
Zufsp	355.6909401	211.0703341	1.68517732	1.23E-05	0.00039141
Slc27a5	18.3186419	10.87342918	1.684716165	0.222863526	0.601423236
Zfp26	868.7040419	515.7202529	1.684448181	2.43E-07	1.94E-05
Acn9	269.5215879	160.0174462	1.684326267	6.49E-05	0.001391709
Gabrg1	297.1788993	176.5825625	1.682945899	8.63E-05	0.001735615
St8sia6	105.9562483	62.97255905	1.682578092	0.013525028	0.084680599
Dntt	26.94982275	16.01784749	1.682487161	0.117918774	0.401429479
Ddit4l	70.44437812	41.8725978	1.682350316	0.010583173	0.070597089
Pgbd1	165.0899478	98.20074092	1.68114768	0.000517836	0.007088772
Rgr	30.73217201	18.28231853	1.680977824	0.076486058	0.297419609
Hps3	711.3930045	423.5481131	1.679603763	9.92E-07	5.58E-05
Hltf	744.0726326	443.5058476	1.677706476	5.08E-07	3.42E-05
Lnpep	837.6883601	499.3943847	1.677408449	4.71E-07	3.20E-05
Pus10	424.6204722	253.2765974	1.67650891	7.70E-06	0.000269922
Cdc73	1384.370394	825.7536689	1.67649318	6.42E-08	7.18E-06
Ankrd53	82.0416698	48.93875582	1.676415112	0.034131103	0.168438547
Ccser1	1028.79143	613.7010926	1.676372167	2.74E-07	2.11E-05
Zfp280c	852.9209965	508.9067451	1.675986818	4.09E-07	2.88E-05
P2ry1	271.3015716	161.8921608	1.675816607	0.001499057	0.016079693
Dio2	25.31261	15.10656574	1.675603207	0.129928099	0.430001681
Lig4	636.87053	380.152485	1.67530282	1.32E-06	6.91E-05
Rps16-ps2	39.51408569	23.59724127	1.674521408	0.061589324	0.255132404
Bbs10	129.6449067	77.47511525	1.673374815	0.001551947	0.016500168
Zfp780b	196.902659	117.7812168	1.671766215	0.000330889	0.004987975

Alpk1	90.55588907	54.19661286	1.67087728	0.015342633	0.093308547
Il22	44.28314118	26.50471166	1.670764872	0.047865131	0.214154714
Zbtb6	918.2356928	549.6436546	1.670601826	3.11E-07	2.32E-05
Rpgrip1l	706.8680126	423.3163489	1.669833954	1.08E-06	5.93E-05
Ppp4r4	215.0353729	128.8133588	1.669356151	0.000178399	0.003085728
Rab39	124.5990439	74.64212237	1.66928592	0.002316293	0.022309783
Cabyr	154.6469381	92.64361846	1.669267033	0.000898337	0.010827416
Mis18bp1	600.3160402	359.6486816	1.669173477	0.001239375	0.013907438
Ccdc138	233.6533431	140.0078992	1.668858289	0.000141083	0.002575416
Crispld1	298.6985018	179.0182299	1.668536785	6.30E-05	0.001364261
Ptbp3	1257.959439	754.1401096	1.668071255	9.73E-08	9.85E-06
Zfp626	65.6117408	39.35413544	1.66721337	0.015355315	0.093364261
Txlng	530.5038581	318.4256216	1.666021269	3.38E-06	0.000144038
Rbm48	270.51682	162.4621088	1.665107156	6.66E-05	0.00141934
Sgol2	205.6823942	123.5585939	1.664654701	0.000190384	0.003242267
Hmgn5	497.4390507	298.9318241	1.664055181	0.000392967	0.005693947
Casp8ap2	1220.169318	733.296597	1.663950607	1.18E-07	1.14E-05
Zcchc10	159.8364112	96.08382665	1.663510049	0.000702655	0.008961805
Gpr34	136.7430152	82.22080988	1.663119293	0.019485002	0.11154238
Rbm11	183.6871143	110.4819535	1.662598357	0.000431737	0.006141594
Zfp322a	895.055481	538.4594654	1.662252293	5.79E-07	3.80E-05
Slc26a2	430.7541225	259.2867127	1.661304268	7.68E-06	0.000269772
Mbtps2	728.5593747	438.6240629	1.661010957	9.51E-07	5.46E-05
Dync2h1	1339.268311	806.5891019	1.660409629	2.30E-06	0.000107482
Fam35a	248.4094815	149.62836	1.660176463	0.000125011	0.002331073
Phf16	822.1658629	495.327615	1.659842573	7.28E-07	4.58E-05
Hnmt	149.7697199	90.24901676	1.659516361	0.001278785	0.014187932
Spag16	57.24609642	34.5047599	1.659078243	0.025130448	0.134643421
Cmc1	284.3237516	171.4030378	1.65880229	6.44E-05	0.001385649
Pcdhb18	437.0695115	263.5421336	1.658442639	1.19E-05	0.00038
Pabpc4l	17.56831027	10.59435778	1.658270433	0.198338298	0.559661415
Zbtb32	31.63493331	19.07876329	1.658122847	0.094767955	0.345648843
Csmd3	632.5504645	381.5069453	1.658031321	0.040693527	0.191385168
Ccdc55	732.2741203	441.6543914	1.658025222	9.94E-07	5.59E-05
Ttc14	1331.779663	803.3211788	1.657842091	3.69E-06	0.000154456
Catsper2	135.5338036	81.76743564	1.657552332	0.001693481	0.017637209
Zfp868	587.5356604	354.4624634	1.657539856	1.93E-06	9.28E-05
Narg2	346.5708364	209.0967682	1.65746625	2.18E-05	0.000611373
Fam26e	55.12163706	33.26013757	1.657288306	0.031323712	0.157964921
Oacyl	21.24744076	12.83307104	1.655678574	0.179400373	0.526878124
Trp53inp1	934.1676272	564.4871625	1.654896142	3.14E-07	2.34E-05
Bmpr1b	136.7231132	82.63557569	1.654530897	0.008303299	0.059106113
Mpp7	506.2682055	306.004492	1.654446973	5.35E-06	0.000205737

Myocd	39.13158765	23.65985643	1.653923293	0.070645574	0.280803186
Brca2	741.1418845	448.1408286	1.653814688	9.36E-07	5.41E-05
Mga	2656.849449	1607.119708	1.65317458	1.17E-07	1.14E-05
Zic4	61.70888926	37.33011308	1.653059264	0.019192852	0.11021577
Casc5	758.0351358	458.5849746	1.652987293	0.002336764	0.022474294
Taf4b	187.0669165	113.1789413	1.652842078	0.000427671	0.006090289
Greb1l	440.996455	266.8938981	1.652328728	0.00231783	0.022315576
Has3	376.8147784	228.2019534	1.651233799	0.000214845	0.003542805
Fpgt	550.2957188	333.4042034	1.650536236	4.12E-06	0.000168733
Tenm1	1219.024378	738.6273493	1.650391608	0.020805379	0.117395945
Klhl40	18.99414146	11.51190955	1.64995576	0.219569857	0.595284966
Snord91a	34.37055577	20.83381127	1.64974883	0.086204989	0.323311386
Mlc1	42.35081009	25.67729816	1.649348379	0.508182162	0.926226179
Pign	867.6480577	526.1726462	1.648979786	8.19E-07	4.92E-05
Tab3	1243.968109	754.6191859	1.648471352	3.36E-07	2.47E-05
Gin1	187.352188	113.7161838	1.647541992	0.000368632	0.005421319
Tex26	23.52435035	14.28994125	1.64621743	0.165426434	0.500336184
Zbtb41	2326.653067	1413.394951	1.646145025	7.38E-08	7.90E-06
Cbln3	38.42059944	23.35540863	1.645040772	0.073131983	0.288009454
Tmem150b	30.99955235	18.84633568	1.644858337	0.136862182	0.444567892
Ly75	83.28867497	50.64019802	1.644714638	0.018277316	0.106187843
Slc35a3	429.2912317	261.1489437	1.643855899	9.85E-06	0.000326726
Synj2bp	694.3154809	422.4155313	1.643678865	1.90E-06	9.20E-05
Zfp799	631.6999756	384.3320865	1.643630594	2.58E-06	0.000116793
Ano3	255.7224069	155.5897278	1.643568702	0.001424773	0.015432713
Haus6	700.9421177	426.5237903	1.643383402	1.33E-06	6.95E-05
Esyt3	49.20896278	29.95150048	1.642954843	0.08165242	0.312063357
Taf1d	502.8933268	306.1851499	1.642448456	6.45E-06	0.000235479
Abca8b	120.0196241	73.0881519	1.64212148	0.003502059	0.030718949
Plek2	19.14303043	11.66535496	1.641015682	0.191213935	0.546543776
Mut	521.4668625	318.0477554	1.639586677	5.51E-06	0.000209328
Dpm1	210.8219229	128.6139616	1.639183804	0.005564382	0.043600816
Crebrf	933.4675109	569.7313925	1.638434398	7.30E-07	4.58E-05
Lrrc29	32.53909792	19.85991437	1.638430928	0.095267473	0.346945791
Zfp711	1212.531904	740.3216192	1.637844786	5.50E-07	3.64E-05
Ctbs	100.7621041	61.5233993	1.637785059	0.00459649	0.037675586
Zfp941	554.5210273	338.6723552	1.637337736	2.90E-05	0.000758092
Duox1	34.1759966	20.87418504	1.637237408	0.109450789	0.381786658
Lrch2	1415.142769	864.6690747	1.636629331	2.97E-07	2.25E-05
Glt8d2	39.06448567	23.87591701	1.636145982	0.152325354	0.47429064
Malt1	304.2967433	186.011026	1.635907021	0.000172302	0.003005768
Dclre1c	335.3162182	205.000343	1.635686133	5.20E-05	0.001178152
Eif5a2	750.9177323	459.1162019	1.635572278	2.04E-06	9.73E-05

Gpr21	619.5345726	378.8187978	1.635437777	5.17E-06	0.000200813
Zfp874a	208.1137443	127.2816297	1.63506505	0.000348463	0.005196758
Zfp873	157.1882744	96.20316832	1.633919933	0.000999232	0.011781078
Lysmd3	526.550413	322.2948867	1.633753543	5.47E-06	0.000208436
Gabra1	269.1655273	164.8034439	1.633251836	0.000177115	0.003066037
Trmt11	235.1482666	144.170456	1.631043372	0.00018958	0.003232741
Pi4k2b	188.1460113	115.3550109	1.631017239	0.002066438	0.020490189
Ptprh	43.55147581	26.72390724	1.629682195	0.058542707	0.246517137
Slc10a3	41.30620011	25.34846226	1.62953475	0.06204319	0.256251906
Rbm41	436.1055992	267.6302272	1.629508011	2.03E-05	0.000579984
Lrrc9	215.0288997	131.9831665	1.629214584	0.00192438	0.019357683
Lmo3	26.37584513	16.19843464	1.628295925	0.137233964	0.445070424
Mettl21c	32.53909792	19.99004503	1.627765114	0.107816493	0.377756622
Mpp5	801.3877571	492.5666845	1.626962972	1.63E-06	8.12E-05
Fam217a	21.27413063	13.07940837	1.626536157	0.177489806	0.523914025
Aste1	306.9527567	188.7343928	1.626374251	7.93E-05	0.001629593
Slc25a40	229.4304861	141.1408195	1.625543106	0.000288611	0.004480571
Lrrtm2	920.9578223	566.5816659	1.625463508	1.53E-06	7.72E-05
Ranbp17	495.0257798	304.64749	1.624913371	1.46E-05	0.000448003
Ccr1	32.45332968	19.97887467	1.624382265	0.495429148	0.913038594
Ppp1r1c	360.1771756	221.7599847	1.624175688	6.41E-05	0.00138063
Zfp811	437.276222	269.4889703	1.622612686	2.88E-05	0.000755452
Npy5r	25.73059669	15.86753708	1.621587305	0.162529175	0.494837462
Socs4	1205.684729	743.6147364	1.621383587	5.17E-07	3.46E-05
Il1rapl2	40.7118594	25.11359163	1.621108602	0.072110706	0.284960876
Vcl	748.3630264	461.6930432	1.620910337	0.027918899	0.145584568
Mllt3	1370.783478	845.7107407	1.620865637	6.03E-07	3.92E-05
Agpat9	32.46179384	20.05196821	1.618883169	0.094877879	0.345954599
Pcdhb19	541.010573	334.197753	1.618833664	0.000246656	0.003947086
Pmfbp1	260.2058152	160.7369848	1.618829765	0.000793834	0.009859153
Zfyve16	611.0157234	377.5718032	1.61827689	8.13E-06	0.000282325
Rsbn1l	1076.045961	665.0216131	1.618061639	9.11E-07	5.33E-05
Rasa2	1107.524925	684.5186021	1.617961764	9.78E-07	5.55E-05
Ccdc111	230.2253987	142.3109786	1.61776274	0.000325008	0.004921704
Mysm1	1002.019072	619.9694641	1.616239396	1.55E-06	7.81E-05
Iltifb	91.04022312	56.33127814	1.616157597	0.009843702	0.066944242
Zfp141	406.4912778	251.5911123	1.615682184	2.98E-05	0.000775706
Igsf6	36.20203416	22.41002314	1.615439392	0.081866037	0.312384491
Itpa-ps1	18.89153027	11.69563171	1.615263779	0.207758384	0.57522411
Thoc2	2641.287842	1635.746885	1.614728945	1.66E-07	1.48E-05
Mef2c	226.3083638	140.1661599	1.614572048	0.002667921	0.025032762
Cav2	194.266694	120.3459416	1.614235524	0.004430697	0.036668027
Depdc7	49.11299243	30.43478439	1.613712514	0.058086772	0.244986298

Sdcbp2	31.56119048	19.56379824	1.613244529	0.106469814	0.374324262
Cep70	744.9541091	461.7968227	1.613164215	3.94E-06	0.000162421
Ap4e1	709.711566	439.9612244	1.613122991	5.33E-06	0.000205737
Kif20b	598.7413612	371.2433861	1.612800076	0.002263349	0.021951341
Kcnmb4	115.8701959	71.85330167	1.612593899	0.004506305	0.037166157
Wdr72	66.64225278	41.32770132	1.612532288	0.024879551	0.133719307
Chuk	955.9316447	592.9445968	1.612177006	2.86E-05	0.000751113
Homer1	1305.134402	809.8011909	1.611672614	1.01E-06	5.64E-05
Sst	16.41758951	10.19701807	1.610038288	0.246847655	0.638561718
Twistnb	387.0834861	240.4964259	1.609518664	4.19E-05	0.001009754
Abca5	1345.012279	835.7433395	1.609360452	6.38E-07	4.11E-05
Grik2	641.5758984	398.6964345	1.609183937	7.56E-06	0.000266426
Sema3a	1514.487674	941.2368335	1.609039957	8.17E-07	4.92E-05
Scn7a	80.8417587	50.24424227	1.608975577	0.116565724	0.397793659
Gabrb1	101.4670591	63.081818	1.608499284	0.018209575	0.105956791
Zfp114	29.13815745	18.11633307	1.608391573	0.122828402	0.413087251
Slc25a18	30.6451475	19.05789157	1.608003036	0.218186059	0.592890093
Mtpap	603.1996917	375.1459078	1.607906895	8.40E-06	0.000288403
Zfp420	338.0274552	210.2481316	1.607754859	8.07E-05	0.001653093
Fdxacb1	118.3469227	73.62158168	1.607503126	0.00473472	0.038546468
Dcaf17	821.2445207	511.0403227	1.607005327	3.51E-06	0.000148581
Trdmt1	232.7621461	144.8757034	1.606633415	0.000405616	0.005831049
Pou2f1	1160.286312	722.3214357	1.606329613	2.77E-05	0.000734402
Mmp16	876.7138575	545.7874657	1.60632831	5.67E-05	0.001257146
Klhdc1	203.5333823	126.7656403	1.605587932	0.000777768	0.009704693
Zfp583	158.8056057	98.94328357	1.60501653	0.001617985	0.017045092
Nsun6	327.9059397	204.3020011	1.60500601	0.000120798	0.002268469
Brcc3	827.2031792	515.4718396	1.604749505	2.73E-06	0.000121469
Mtf2	1301.005337	810.822183	1.604550744	7.75E-07	4.74E-05
Stard9	599.9949236	374.0285067	1.604142232	0.034349487	0.169295724
Tsga10	431.7446424	269.2182728	1.603697394	2.82E-05	0.000743109
Fcho2	797.9072287	497.7883196	1.602904683	3.28E-06	0.000141141
Slco5a1	1177.437225	734.6361307	1.602748866	1.26E-06	6.67E-05
Zfp804b	148.4290699	92.66587415	1.601766252	0.047419867	0.212611634
Btaf1	1729.514666	1080.016943	1.601377346	5.50E-07	3.64E-05
Hapln1	16.12783519	10.07904607	1.600135079	0.270377298	0.674852606
B3galt1	1121.534858	700.9260224	1.600075932	2.09E-06	9.94E-05
Mc1r	32.7281055	20.45420827	1.60006709	0.119413215	0.405060868
Znrd1as	45.58105407	28.48836026	1.599988685	0.06184387	0.255816593
Pfn4	106.8571451	66.7906192	1.599882535	0.007793817	0.056557183
Hells	664.1535782	415.3275411	1.599107963	0.003358093	0.029750486
Gipc2	145.6357107	91.19615329	1.59695015	0.00307456	0.027900421
Efcab2	160.1839023	100.3246172	1.596656003	0.001895213	0.019115053

Dzip3	1944.537934	1218.492606	1.595855342	5.92E-07	3.86E-05
Baz1a	709.9281851	444.8857973	1.595753763	5.77E-06	0.000216243
Hrh1	23.56647985	14.77184119	1.595365096	0.173300891	0.51472915
Cenpe	1532.853263	960.8432083	1.595320911	0.000785456	0.009782599
Ythdc2	1082.146428	678.3740205	1.595206177	2.80E-06	0.000123454
Cntnap4	843.5615233	528.8684478	1.595030913	1.37E-05	0.000424506
Rhox5	54.58038258	34.22501081	1.594751361	0.043586049	0.200299425
Zfp248	641.3625053	402.2250442	1.594536478	1.11E-05	0.000359295
Nkap	992.2077993	622.3316566	1.59433927	3.08E-06	0.000133675
Mfsd2b	123.4023389	77.40868739	1.594166534	0.005864257	0.045321082
Zfp472	242.9072102	152.3876381	1.59400863	0.002167261	0.021244768
Ppargc1a	418.6166039	262.6955429	1.593542849	6.90E-05	0.001460831
Rrm2b	646.356163	405.7163301	1.59312336	1.26E-05	0.000397128
Prtg	181.3836825	113.8619188	1.593014455	0.001263817	0.014074817
Slc7a10	19.23684283	12.0759267	1.592991024	0.248141653	0.640300123
Slc4a7	2085.064197	1309.542839	1.592207704	3.80E-07	2.73E-05
Star	247.4783335	155.4572248	1.59193845	0.000464883	0.006504932
Crybg3	263.3711974	165.4415851	1.591928639	0.000263269	0.004155269
Zfp354b	63.23932198	39.74697047	1.5910476	0.031938285	0.1601638
Tmem47	792.8280346	498.4031325	1.59073646	5.67E-06	0.000213305
Ppp2r3a	1723.684071	1083.861354	1.590317862	9.81E-07	5.56E-05
Prox2	32.59356691	20.49525973	1.590297822	0.105779712	0.372773152
Carf	284.3768379	178.8258512	1.590244565	0.000238648	0.003842124
Myo3b	222.6747365	140.0718983	1.589717418	0.000763847	0.009571913
Arntl2	66.73298561	41.99264574	1.589158874	0.035164425	0.172095827
Mog	148.5902983	93.51071374	1.589018973	0.02633649	0.139486298
Rock1	1922.258325	1210.592693	1.587865461	7.28E-07	4.58E-05
Ino80d	1617.032946	1018.490677	1.587675747	2.65E-05	0.000710893
Rp2h	473.8994489	298.690627	1.586589622	3.21E-05	0.000821197
Bambi-ps1	19.70326524	12.42249931	1.586095097	0.209033051	0.577186336
Ccdc39	395.1869677	249.1770936	1.585968285	7.28E-05	0.00152045
Arhgap18	336.8159181	212.4534331	1.585363499	0.000139453	0.002554606
Macrod2	1281.916652	808.6592183	1.585237171	2.14E-06	0.000101405
Slc24a5	41.29463581	26.05445814	1.584935507	0.077246345	0.299235074
Bcap29	338.7671031	213.8511902	1.584125404	0.0001096	0.002099618
Trip4	1557.802147	983.5879707	1.583795445	1.66E-06	8.23E-05
Pclo	3302.364796	2085.452061	1.583524675	0.026481734	0.140129648
Etohd2	54.22595716	34.25459558	1.583027218	0.044057346	0.202114982
Tmem220	17.40044097	10.99277084	1.582898545	0.245661798	0.637523216
AI987944	97.41347471	61.55642969	1.5825069	0.009068274	0.063012144
Ttbk2	2074.573275	1310.969659	1.582472378	9.68E-07	5.53E-05
Rttn	438.5781778	277.3376062	1.581387334	5.25E-05	0.001187484
Pcdhb21	297.304325	188.0513303	1.580974325	0.000214836	0.003542805

Trpc6	96.49634247	61.10061243	1.579302377	0.013686705	0.085292306
Mob1b	1038.791689	657.7849506	1.579226901	2.65E-06	0.000119124
Slc38a6	192.6742299	122.0158081	1.579092356	0.001339117	0.014704931
Shox2	69.93226496	44.2925902	1.578870521	0.028635504	0.148583751
Ovol1	19.43772877	12.31393234	1.578515151	0.227653244	0.608574796
Kif27	100.481128	63.67266628	1.578088902	0.011700777	0.075781301
Zfp788	712.5023909	451.5914587	1.577758784	9.97E-06	0.000329242
Snhg7	94.11866557	59.67438605	1.577203752	0.010773067	0.071558512
Tmem68	695.284149	440.9358697	1.576837352	1.19E-05	0.000380987
Met	238.3310582	151.1659492	1.576618673	0.000422937	0.006042344
Krt222	185.8418043	117.8741087	1.576612594	0.001729872	0.017861868
Csgalnact2	445.0819456	282.3989048	1.576075325	4.33E-05	0.001032368
Pms1	300.4009726	190.6248389	1.575875286	0.000200849	0.003376766
Ercc6l2	710.1626952	450.8304874	1.575232188	1.05E-05	0.00034248
Rabl3	383.9874049	243.7702815	1.575201877	0.000102617	0.00199049
Eif4a-ps4	107.8584953	68.49626975	1.574662323	0.016737419	0.09953115
Akap5	723.6952306	459.6512698	1.574444102	0.000339447	0.005088064
Zfp386	816.477162	518.7464437	1.573942669	6.35E-06	0.0002337
Manea	555.6419574	353.340247	1.572540808	2.30E-05	0.000633481
Zdhhc21	1406.448967	894.6917476	1.571992779	2.38E-06	0.000110839
Ppl	20.19576994	12.85080775	1.57155646	0.246485579	0.638339007
Alg6	340.7718559	216.8790192	1.571253214	0.00018486	0.003178826
Fastkd5	128.512077	81.7938854	1.571169732	0.004799785	0.038944642
Trip11	1643.766002	1046.568184	1.570624855	7.99E-06	0.000277977
Ptchd1	299.9771203	191.0072098	1.570501556	0.000307447	0.004715748
Dusp12	198.4765834	126.378002	1.570499456	0.001269693	0.014116214
Vwa7	50.24441879	31.9950106	1.570382939	0.050531796	0.222390226
Jpx	155.1577745	98.80825256	1.570291655	0.002613069	0.024596424
Gpr137c	335.1281117	213.4371021	1.570149278	0.000152684	0.002741084
Arap2	183.5817582	116.9391309	1.569891591	0.07121371	0.28246862
Ppil3	264.4988743	168.5164392	1.569573127	0.000447517	0.006308537
Srek1ip1	494.6442034	315.2192672	1.569206755	3.80E-05	0.000937981
Naa16	379.5078514	241.9661324	1.568433762	0.00010037	0.001954056
Grhl1	38.01419761	24.24512667	1.567910868	0.097546141	0.352794137
Fmn1	358.3354309	228.5676324	1.567743548	0.001516332	0.016219067
Mier3	1018.217925	649.4926275	1.56771283	4.73E-06	0.000188093
Wdr44	633.8821512	404.3516742	1.567650616	2.77E-05	0.000734402
Xkr4	1295.032431	826.1830504	1.567488501	0.008450278	0.059863095
Fam111a	468.9535577	299.2041733	1.567336286	4.74E-05	0.00110774
Larp1b	369.0897731	235.4929129	1.567307349	0.000124459	0.002325317
Actr6	409.2012791	261.0869641	1.567298775	0.00010512	0.002026805
Tctex1d2	199.0200164	127.012359	1.566934257	0.001245739	0.013955225
Snx31	16.68807	10.65246826	1.566591855	0.295246769	0.706813535

Mtx3	744.0645242	475.101711	1.56611628	1.51E-05	0.000461497
Pigw	222.1059964	141.8241783	1.566065808	0.000860519	0.010500475
Mkln1os	99.12549897	63.30904894	1.565739821	0.029778927	0.152612687
Zfp40	529.0379289	338.0144292	1.565134158	4.41E-05	0.001045437
Tmx3	1067.998311	682.5606684	1.564693602	3.72E-06	0.000155248
Nsun3	442.4728381	282.9044586	1.564036284	8.32E-05	0.001691363
Phykpl	590.2619831	377.5147517	1.563546803	0.015233047	0.092791056
Tceanc	229.2136789	146.6092441	1.563432649	0.003149711	0.02844001
Crip3	53.2887759	34.08478313	1.563418365	0.048216239	0.215199764
Rbm12	905.0101137	579.1890029	1.562547129	7.88E-06	0.00027513
Fsd1l	1897.103967	1214.396448	1.562178455	1.68E-06	8.27E-05
Zdhhc15	348.198434	222.9360889	1.561875584	0.0001948	0.003302127
Hykk	135.9451701	87.05698198	1.56156539	0.006107617	0.046819763
Gcc2	1716.530951	1099.425277	1.561298423	2.60E-06	0.000117336
Dcun1d1	648.8214308	415.6378497	1.561025858	1.99E-05	0.000573091
Snrnp48	411.4828392	263.6236295	1.560872369	0.000113912	0.002166776
Ptpn3	318.1664817	203.8552075	1.560747383	0.000736684	0.009310728
Cdnf	32.72425073	20.97648198	1.560044757	0.204211006	0.569617358
Dsel	594.8911354	381.3915436	1.5597911	4.06E-05	0.000986105
Gyk	447.3387856	286.8345878	1.559570584	6.84E-05	0.001452795
Rgs7bp	751.2463695	481.8232452	1.559174193	0.010826668	0.071841823
Ero1lb	345.2030354	221.4534752	1.558806133	0.000276054	0.004328704
Ednrb	347.9516866	223.2262735	1.558739843	0.120646189	0.408042646
Ptplad2	71.03871883	45.59386828	1.558076152	0.116050857	0.396658858
Ndst4	451.2337811	289.9159661	1.556429565	0.001944291	0.019520948
Ppef1	230.5696015	148.1780848	1.556030379	0.004545256	0.037380934
Plek	532.5819126	342.2855387	1.555957972	0.000427296	0.006088219
Rabgap1l	1998.42618	1284.645436	1.555624707	0.000489242	0.006795568
Iqcb1	708.0678383	455.1693808	1.55561395	2.15E-05	0.000605839
Tnfrsf11b	43.50859169	27.97304855	1.555375404	0.084519491	0.319065892
Ccdc112	676.766151	435.1219458	1.555348236	3.02E-05	0.000780793
Wwp1	1196.208021	769.1598841	1.555213741	6.16E-06	0.000227945
Pi15	25.07162601	16.13161112	1.554192314	0.541192714	0.954520819
Krt26	44.42926463	28.58821417	1.554111228	0.084868629	0.320064936
Jhdm1d	1468.495818	944.9471738	1.554050701	3.29E-06	0.000141246
Ccdc160	84.44850858	54.34135952	1.554037465	0.021935543	0.122322367
Bdnf	709.6264259	456.6892482	1.553849644	0.00439445	0.036459084
Zfp280d	1176.497132	757.186438	1.553774701	4.87E-06	0.000191806
Smc5	1580.62171	1017.46141	1.553495488	3.52E-06	0.000149004
Nr6a1	339.5046964	218.62743	1.552891585	0.003336686	0.029632276
B3gnt5	1299.004169	836.5119648	1.552881756	4.64E-06	0.000186047
Pot1b	444.4655038	286.2601774	1.552662714	8.51E-05	0.001716439
Zfp62	1056.657276	680.7290955	1.552243444	5.92E-06	0.000220685

Fam19a1	216.3368059	139.3892736	1.552033383	0.000905722	0.010906508
Slx4ip	607.4911805	391.6887969	1.550953679	4.68E-05	0.001098504
Ebf2	379.7002732	244.869748	1.550621407	0.003971755	0.033896063
Rmdn2	151.1480164	97.47662697	1.550607783	0.003984046	0.033961387
Hspa1l	54.62079469	35.2462292	1.549691866	0.055508832	0.237360454
Il1a	96.32478542	62.18174879	1.549084535	0.36867456	0.791688909
Sfxn4	160.8148003	103.8810463	1.548066813	0.003439059	0.030336142
Tpo	16.4263883	10.61385983	1.547635693	0.320175388	0.739053928
Kcnj2	60.88343205	39.35482743	1.547038471	0.045003488	0.205106993
Lpp	289.5299829	187.1601576	1.54696377	0.032374871	0.161849941
Arid4b	2502.239363	1617.678608	1.546808711	2.21E-06	0.000104294
Cdkl3	453.5660813	293.3193026	1.546321968	0.000116117	0.002203987
Fam229b	159.3493116	103.0609055	1.546166424	0.004023032	0.034201578
Ndst3	1201.274814	777.1997706	1.545644838	4.83E-05	0.001121199
Gpr158	577.8269558	373.8827296	1.545476456	0.002237635	0.021813653
Nmrk2	22.77263596	14.73705976	1.545263189	0.290889771	0.701769869
Rb1cc1	2148.660375	1391.128446	1.54454492	2.91E-06	0.000127809
Atm	1060.637555	686.72965	1.544476134	7.86E-05	0.001617502
Rbm12b2	494.6281973	320.4086068	1.543741918	0.000101815	0.001977828
Mipol1	122.7158826	79.50471565	1.543504453	0.007688661	0.055962813
C1qtnf9	16.24370751	10.5254726	1.543275834	0.282895601	0.692765031
Muc1	23.55489498	15.26314617	1.543252926	0.207419549	0.57485484
Tatdn1	216.7808749	140.4755788	1.543192609	0.00138557	0.015119248
Odf2l	303.7333872	196.8524695	1.542949336	0.010850926	0.071966773
Npy1r	161.853063	104.920637	1.542623717	0.116512342	0.397791318
Atxn3	811.7044188	526.2509929	1.542428289	1.60E-05	0.000481671
Abcb7	1078.156069	699.0729137	1.542265547	9.12E-06	0.000306528
Slitrk6	387.6079799	251.3391969	1.542170838	0.000185479	0.003187401
Milr1	24.56860514	15.93259527	1.542034096	0.193472269	0.550618299
Ttr	37.09631074	24.05757751	1.541980306	0.101320428	0.361589835
Zfp948	563.6263606	365.5539869	1.541841645	7.19E-05	0.00150661
Catsperg1	46.10626135	29.90731401	1.541638321	0.300603792	0.712825089
Polq	386.4847242	250.7295951	1.541440387	0.000157468	0.002809006
Trappc2	223.4980358	145.0333429	1.541011407	0.001168311	0.013272899
Zfp354a	385.1610019	249.967946	1.540841568	0.000169196	0.002975151
Zfp619	265.5562643	172.3954199	1.540390484	0.000556187	0.007464175
Tmtc3	823.5776577	534.8866406	1.539723738	2.05E-05	0.000585724
Sema3d	72.07247801	46.81273284	1.539591339	0.13253916	0.435328671
Nr3c2	135.3128687	87.89063692	1.539559542	0.005611689	0.043893668
Phip	4074.151286	2646.754718	1.53930066	1.56E-06	7.83E-05
Ccdc175	62.36431924	40.51802454	1.539174724	0.041733495	0.194448316
Hapln4	52.40931082	34.05519836	1.538951859	0.160573784	0.490357533
Arhgap5	1458.872358	948.0829342	1.538760277	5.26E-06	0.000203561

Sgk3	145.6209198	94.6610459	1.53834049	0.003990714	0.033992218
AW146154	49.33116188	32.07154977	1.538159591	0.076219308	0.296678158
Eea1	1513.379083	984.0295533	1.537940683	6.41E-06	0.000234575
Cnot6l	1991.313781	1294.861716	1.537858257	3.82E-06	0.000158749
Cftr	99.7841967	64.90024388	1.537501105	0.015840977	0.095420117
Plag1	229.5141375	149.2957536	1.537311893	0.012027589	0.077492664
Rnf152	782.9436894	509.4184417	1.536936289	0.003492596	0.030702705
Ankdd1b	196.0508054	127.5659121	1.536858885	0.002529861	0.023983481
Krcc1	245.666757	159.8984574	1.53639229	0.000834081	0.010253354
Gsta3	15.42070174	10.03730263	1.536339224	0.321823976	0.740959216
Fnip1	1268.718074	825.9088654	1.536147785	7.01E-06	0.000250975
Gpr88	138.7614902	90.33496095	1.536077381	0.042294906	0.196202192
RP23-344G11.3	22.61524171	14.72382773	1.535962124	0.234329561	0.619119434
Thoc1	1335.445217	869.8288289	1.53529657	7.63E-06	0.000268747
Alms1	1071.594941	698.0433629	1.535140936	4.50E-05	0.001061524
Mfsd7b	650.2550332	423.6636278	1.534837995	5.20E-05	0.001178152
Lactb2	118.9798111	77.56525352	1.533931828	0.008699864	0.061222294
Tmem252	29.61061313	19.30492088	1.533837581	0.167269044	0.503840277
Sepsecs	555.9681432	362.4892156	1.533750852	7.13E-05	0.001497054
Palmd	55.71983254	36.34367613	1.533136943	0.049186015	0.218242363
Tnrc6b	3567.368035	2327.152045	1.532932944	6.39E-06	0.000234179
Ift88	638.9579099	416.927986	1.532537828	5.78E-05	0.001278673
Epha1	39.83515984	25.99390464	1.532480803	0.118512161	0.402813768
Nr1h3	37.01730984	24.15673945	1.532380225	0.107535626	0.376921965
Zfp955b	241.7878299	157.8090097	1.532154788	0.001285883	0.014254376
Samd15	51.08037161	33.34156279	1.532032914	0.076803794	0.298130495
Snap23	348.7497647	227.6692435	1.531826431	0.000323242	0.004900561
Bmp2k	705.1091846	460.3882766	1.531553301	4.01E-05	0.000974939
Slc44a5	290.3953705	189.6199311	1.531460163	0.000816512	0.010079439
Zc2hc1c	129.3835185	84.49080249	1.531332579	0.009031769	0.062824342
Hydin	77.39345121	50.55946478	1.53074111	0.157870658	0.485352195
Klf8	688.1060974	449.6038845	1.530471869	0.000450741	0.006337341
AK129341	559.6926298	365.7916391	1.530085901	6.37E-05	0.001376467
Nek11	54.58285459	35.675611	1.529976728	0.064024339	0.262228818
Mid2	692.6743281	452.8690271	1.529524623	0.003861309	0.033134776
Ube3a	1994.518009	1304.083142	1.529440834	5.25E-06	0.000203398
Nxnl1	48.87938335	31.97375751	1.528734411	0.073575324	0.289239925
Efcab1	244.3333555	159.8709485	1.528316168	0.001242267	0.013927854
Herc4	905.091252	592.2288289	1.528279624	2.32E-05	0.000638729
Ido2	37.82380728	24.75172533	1.528128111	0.121116892	0.409008597
Ptpn4	845.7968468	553.5252052	1.528018668	6.96E-05	0.001469047
RP24-390G17.1	184.7882896	120.9441189	1.527881564	0.002775115	0.025769338
Rif1	2129.013485	1394.268019	1.526975772	3.83E-06	0.000158749

Kif18a	496.7087886	325.3647976	1.526621172	0.004339486	0.036132506
Stk17b	256.7064735	168.2196875	1.526019203	0.019206847	0.110259525
Slc33a1	294.559108	193.1481165	1.525042611	0.000590773	0.007852093
Papolg	612.0473246	401.5038111	1.524387335	6.30E-05	0.001364261
Zfp952	625.3660674	410.3948906	1.523815432	6.94E-05	0.001465294
Zfp54	74.4866917	48.89072807	1.523534106	0.034696907	0.17046937
Fbxo30	1100.093193	722.3998109	1.522831508	1.68E-05	0.000497018
Snx13	1035.062091	679.759209	1.522689325	1.97E-05	0.000566228
Baiap2l1	79.98401891	52.54281705	1.52226362	0.137438652	0.445306645
Dhtkd1	315.2332205	207.0984758	1.522141673	0.004920275	0.039715684
Mospd2	479.2235222	314.9067983	1.521794781	0.000136529	0.002514023
Dppa5a	34.45835546	22.64490807	1.521682285	0.155056235	0.479583708
Cenpp	145.6123939	95.70283968	1.521505468	0.030118067	0.153874889
Gpr182	61.62496489	40.50411482	1.521449491	0.052170681	0.227376361
Atad5	995.8555663	654.9978953	1.520395063	1.69E-05	0.000500198
Tmem167	1960.104841	1289.618644	1.51991044	6.54E-06	0.00023841
Ccdc77	388.3126002	255.5042253	1.519789348	0.000319209	0.004845592
Ccdc66	462.9296411	304.6214638	1.51968819	0.000145606	0.002637227
Eif5b	2386.865506	1570.729179	1.519590734	5.67E-06	0.000213305
Fam227a	369.2551915	243.0079548	1.519518947	0.005375277	0.042419894
Stxbp5l	434.3849438	285.8964328	1.519378677	0.00693732	0.051642333
Spats1	31.58540834	20.78893282	1.51933765	0.165346346	0.500265143
Esco1	703.2871979	462.9172738	1.51925028	5.02E-05	0.001154051
Rmi1	209.1300941	137.689526	1.518852597	0.002510026	0.023803949
Ccdc14	265.7167382	174.9563177	1.518760464	0.000825526	0.010171764
Pygo1	2551.497977	1680.069019	1.518686404	6.81E-06	0.000245743
Vcpip1	1927.289039	1269.108359	1.518616614	8.27E-06	0.000284905
Mir22hg	78.78360558	51.91823211	1.517455475	0.029829314	0.152811843
Epha7	492.2439207	324.4552111	1.51713982	0.000793531	0.009859153
Morc4	295.0704466	194.5782407	1.516461684	0.000856086	0.010465643
Dsc2	26.03053256	17.16713488	1.516300346	0.208606405	0.576308338
Guf1	1107.093847	730.2263898	1.516096737	0.000142733	0.002595815
Col24a1	52.72960978	34.78833598	1.515726702	0.122563452	0.41268476
Usp45	757.6398577	500.0608403	1.515095358	4.62E-05	0.001086637
Cenpf	2162.776922	1427.751093	1.514813704	0.005793693	0.044893501
Tfcp2l1	83.94829434	55.42207234	1.514708685	0.084987327	0.320331046
Usp38	945.6720487	624.3834841	1.514569287	2.92E-05	0.000763269
Grin2b	2228.456035	1471.56928	1.514339872	0.124163194	0.416116184
Adm	20.8336229	13.76139752	1.513917673	0.246189992	0.637933533
Pfkfb2	1009.929691	667.1214969	1.513861711	2.82E-05	0.000743109
Dock11	1461.095868	965.1950371	1.51378303	1.30E-05	0.000408871
Ppwd1	352.8766308	233.1891272	1.513263654	0.000377977	0.005532159
Tmc3	136.0852602	89.94425834	1.512995524	0.008876888	0.062030296

Asxl2	1165.33622	770.2998937	1.512834456	2.08E-05	0.000592595
Camk4	2470.962851	1633.496705	1.512683095	0.035980802	0.175058155
Tmod4	18.26664493	12.08150473	1.511951146	0.277461423	0.684323979
Polr3f	887.4985973	587.0290959	1.511847715	2.87E-05	0.000754387
Rnf138	739.6344128	489.2844407	1.511665508	5.58E-05	0.001243657
Pola1	750.6164778	496.574087	1.511590108	0.003040228	0.027674114
Osgepl1	332.2414218	219.8101006	1.51149297	0.000578986	0.00772642
Mttp	218.7545809	144.7831929	1.51091143	0.002418754	0.023078691
Zbtb44	1434.032894	949.3118529	1.510602538	1.08E-05	0.000350961
Pter	165.7319696	109.7244842	1.510437445	0.011579921	0.075240018
Dmrtc1a	21.4595975	14.20851602	1.510333483	0.26610082	0.667669318
Nol8	905.3814231	600.1835608	1.508507534	4.39E-05	0.001044089
Hmmr	455.8373565	302.1956392	1.508418049	0.067976285	0.272934461
Kdm5a	2530.338545	1677.530361	1.508371236	6.21E-06	0.000228945
Il1rap	104.5708292	69.3285693	1.508336754	0.017257283	0.101678547
Fsip1	48.11224989	31.91496934	1.507513586	0.092923524	0.340667118
Nova1	1673.774701	1110.321315	1.507468765	1.35E-05	0.000418949
Ifit2	264.6826649	175.6247077	1.507092415	0.001256636	0.014023973
Lrrc40	1010.834462	670.805825	1.506895773	3.14E-05	0.00080511
Mier1	1387.139641	920.5333931	1.506886824	1.57E-05	0.000474556
Xpo4	562.0524362	373.022667	1.506751428	0.000169201	0.002975151
Sox5	299.1874863	198.5851346	1.506595581	0.000840975	0.010318971
Lyrm5	225.179536	149.4752817	1.506466711	0.001798206	0.018390116
Tiparp	1013.235268	672.6520846	1.50632889	3.04E-05	0.000784771
Zfp397	1341.619174	890.8370137	1.506020915	1.64E-05	0.000489958
Dio3	85.00273068	56.46354122	1.505444555	0.103201467	0.365987707
Esco2	461.2412265	306.4023117	1.505345126	0.064878047	0.264867833
Pdf	21.22707767	14.1055271	1.504876601	0.254511525	0.648781735
Mctp1	274.3908237	182.3686105	1.504594585	0.009474242	0.064948032
Sfmbt2	113.201696	75.24024327	1.504536549	0.020006165	0.113813233
Rap2b	271.9119184	180.7284416	1.504533077	0.001005853	0.011843335
Pibf1	373.9716213	248.6158444	1.504214755	0.000528388	0.007199765
Taf13	395.8070555	263.2853886	1.503338478	0.000471772	0.006594362
Fancm	1213.435963	807.2364233	1.503197735	2.62E-05	0.000703812
Nespos	20.37706798	13.55611165	1.503164662	0.451626741	0.876560011
Acr	78.92604187	52.50802132	1.503123521	0.0834937	0.316545807
Thumpd2	146.0368956	97.16246352	1.503017629	0.007045453	0.0523151
Ttll13	68.8844489	45.83608229	1.502843294	0.05086726	0.223273675
Bdp1	1694.727172	1127.794253	1.502691797	1.58E-05	0.000479408
Reps2	1258.677926	837.7841385	1.5023893	0.008885553	0.062067147
Cyld	1026.208279	683.0581448	1.502373241	3.81E-05	0.00093977
Spin4	105.2045339	70.0300462	1.502277088	0.01940684	0.11116694
Mphosph8	1065.812994	709.6826502	1.501816331	3.21E-05	0.000821197

Lrat	44.76822985	29.81334875	1.501616951	0.122880133	0.41315628
Pcdhb16	491.0351697	327.0644044	1.501340907	0.00033045	0.004984189
Smarca1	1296.308474	863.4922077	1.501239343	2.99E-05	0.000776913
Rbm46	27.6228503	18.40304416	1.500993534	0.198617472	0.560041904
Trpm7	1619.593596	1079.063155	1.500925676	1.68E-05	0.000497647
Zfp846	594.39046	396.1272194	1.500503957	0.000160828	0.002852522
AW822252	18.67551877	12.44650604	1.500462757	0.286838415	0.698101587
Rfx3	1228.951232	819.0911635	1.500383946	2.74E-05	0.000726244
Zfand4	242.7877972	161.8327228	1.500239216	0.001628837	0.01711861
C2cd2l	1084.054079	1618.299069	0.669872522	2.19E-05	0.000611373
Six3	2230.390043	3329.666987	0.669853788	8.83E-06	0.000299378
Tmem86a	210.9160282	314.8870421	0.669814886	0.114004465	0.391928223
Rltpr	606.7394661	905.8554328	0.669797237	4.44E-05	0.001052258
Fap	12.34611406	18.43607456	0.669671519	0.701626476	1
Rpl3	764.1715105	1141.182416	0.66963134	4.24E-05	0.001017404
Fbxl19	2006.26706	2996.244215	0.66959397	8.48E-06	0.000290657
Cdk5r1	3080.093681	4600.054453	0.669577656	6.37E-06	0.000233939
Dtx3	1406.014413	2099.886738	0.669566786	1.47E-05	0.000450989
Khsrp	2309.227407	3449.304221	0.669476294	9.69E-06	0.000322446
Hmha1	69.17376267	103.3451449	0.669346999	0.178010634	0.52465225
Fzr1	1262.675849	1886.569542	0.669297272	1.55E-05	0.000471892
Tmem151a	841.9238495	1257.952738	0.669280986	0.003009196	0.027476532
Txn1	440.1295606	657.6255458	0.669270778	0.000137992	0.002539211
Atp6v0e2	1199.018414	1791.600151	0.669244426	1.65E-05	0.00049111
Klhdc8b	895.0685128	1337.457991	0.669231123	2.42E-05	0.000657463
Bola1	142.6436441	213.1761631	0.669135057	0.003178399	0.028617465
Dstn	1329.094401	1986.462984	0.669075846	1.54E-05	0.000471185
Pmp22	345.009879	515.6640054	0.669059456	0.095476614	0.347564236
Sncg	888.5790954	1328.455759	0.66888121	2.84E-05	0.000748377
Egr4	322.8501035	482.7190355	0.66881577	0.000264899	0.004176018
Eif3h	868.2371609	1298.178981	0.6688116	3.26E-05	0.000831874
Shroom3	517.8180242	774.3128742	0.668745208	0.00010419	0.00201805
Dgkz	1538.66712	2300.909743	0.668721198	1.30E-05	0.000408871
Dcxr	55.91160562	83.61192991	0.668703685	0.054321763	0.233835766
U2af2	1214.670211	1816.782091	0.668583325	1.90E-05	0.000550884
Tspan3	1319.53268	1973.67698	0.668565674	1.66E-05	0.000494168
Lgmn	1009.478519	1509.962569	0.668545393	0.00513746	0.041118286
Ssc5d	225.9621296	338.0689668	0.66839063	0.078430444	0.302716396
Fance	468.0242536	700.4244792	0.66820088	0.000157606	0.002809006
Angptl4	28.75073595	43.02739247	0.668196103	0.309104575	0.724111398
Megf6	337.5137711	505.1294958	0.668172763	0.133090671	0.436653332
Ppapdc3	322.2037247	482.2513416	0.668124061	0.000254505	0.00405069
Slc25a5	705.348536	1055.715599	0.668123628	4.35E-05	0.001038255

Cpe	2451.538621	3669.353937	0.668111788	7.24E-06	0.000258547
Fzd9	49.21373919	73.66124917	0.668108941	0.108906627	0.380520242
Dctpp1	255.4117667	382.3290066	0.668041823	0.000613303	0.008086698
Iscu	293.9437966	440.0687745	0.667949679	0.000350968	0.005225311
Tubb6	356.1585982	533.3509433	0.667775322	0.109123525	0.381027046
Hnrnpl	2526.002267	3783.001293	0.667724399	7.43E-06	0.000263052
Adrb2	36.00808258	53.92725711	0.667715818	0.355014214	0.778811429
Lasp1	2221.603352	3327.241756	0.667701212	7.38E-06	0.000261581
Kcnk4	67.28938621	100.8092851	0.667491949	0.134448576	0.439693936
Gnb1	3313.608716	4964.43153	0.66746992	5.34E-06	0.000205737
Id3	280.021098	419.5934539	0.667362885	0.29753698	0.709678021
Baiap3	80.03000317	119.9225192	0.667347582	0.012169291	0.078206718
Akt1s1	713.4477223	1069.390756	0.667153441	3.92E-05	0.000960874
Trip6	283.2588197	424.7134705	0.666940983	0.006230208	0.047525949
Efs	394.4943311	591.540654	0.666893016	0.039596633	0.187624074
Raly	1132.429479	1698.242656	0.666824305	1.88E-05	0.00054458
Necab2	769.8766786	1154.987577	0.666567064	2.27E-05	0.000629832
Stk11	1208.926789	1813.732727	0.66654076	1.62E-05	0.000486753
Dpysl3	6267.635266	9403.793411	0.66650074	3.58E-06	0.000150172
Jun	2138.699187	3209.363396	0.666393587	7.31E-06	0.000260289
Cyth4	108.5321715	162.8824526	0.666322061	0.1069123	0.375313947
Ndn	1523.848321	2287.726667	0.666097197	1.02E-05	0.000335061
Hist3h2ba	258.3097274	387.8947857	0.6659273	0.000496107	0.006869536
Fam20c	1333.570141	2002.617544	0.665913542	8.25E-06	0.000284797
Emid1	56.20877598	84.44835278	0.665599436	0.067788608	0.272552347
Sez6l2	2184.864106	3283.11775	0.66548454	4.85E-06	0.000191533
Klhdc3	1218.026718	1830.368762	0.665454275	1.26E-05	0.000397128
Cyr61	425.994203	640.2293578	0.66537749	0.390055392	0.819678933
Mapk7	1119.901521	1683.381821	0.665268869	1.44E-05	0.000444617
Atp5g3	678.0443289	1019.210145	0.665264501	3.85E-05	0.000948143
Tm4sf1	22.48736453	33.80357932	0.665236196	0.665913902	1
Amotl2	2389.916504	3593.245639	0.665113589	6.66E-06	0.000242221
Trmt112	165.4597923	248.8103269	0.665003717	0.001829993	0.018634443
Sapcd1	61.89079487	93.07756141	0.664937864	0.203411041	0.568819379
Nwd1	153.9644631	231.5579399	0.664906862	0.002286576	0.022079696
Cd37	45.31859719	68.17928191	0.664697485	0.059646485	0.249737278
B4galt2	1379.233402	2074.990096	0.664693969	9.45E-06	0.00031513
Rpl18a	328.6048819	494.4271644	0.664617371	0.000224986	0.003664448
Lsmd1	106.9168517	160.8925483	0.664523328	0.005965744	0.045971427
Nr4a1	1932.249219	2907.942215	0.664473045	1.68E-05	0.000497705
Krt1	110.7242145	166.6465685	0.66442541	0.004477467	0.036997359
Vars	1483.36846	2232.702655	0.66438245	0.000128935	0.002390795
Atf4	1276.925613	1922.108441	0.664335886	1.34E-05	0.000416571

Ssr2	615.5650385	926.8246979	0.664165553	5.11E-05	0.001163938
Tkt	1738.741887	2618.11801	0.664118989	7.97E-06	0.000277666
Pias3	800.6830545	1205.723025	0.66406881	2.59E-05	0.000698295
Hmgb3	605.4531175	911.9283041	0.663926226	5.13E-05	0.001169117
Mapk3	965.9302289	1454.88844	0.663920478	1.66E-05	0.000494898
Mrps24	288.2596198	434.20373	0.663881031	0.000366782	0.005403108
Ccnf	285.6310503	430.3113617	0.663777617	0.319386213	0.738254734
Agpat1	1080.204713	1627.515638	0.663713877	1.26E-05	0.000397128
Mycl	1443.386107	2174.720658	0.663711039	0.010889887	0.072189064
Prmt2	1474.514855	2221.636452	0.663706636	7.65E-06	0.000269164
Psmb2	504.4376693	760.07089	0.663671871	7.23E-05	0.001512043
Tomm40l	113.4138527	170.9033869	0.663613839	0.005198145	0.041441376
Irx3	561.1724689	845.6567821	0.663593648	3.66E-05	0.000910122
Trp53i13	157.9108475	237.9637289	0.663592087	0.012239284	0.078532901
Hes7	13.37708717	20.15985748	0.663550681	0.306058485	0.719656485
Tbxas1	43.75870909	65.94683867	0.663545213	0.062350081	0.257118935
Zdhhc8	868.1432432	1308.438149	0.66349582	1.31E-05	0.000411584
Zfp853	26.33480488	39.69230562	0.663473801	0.303767311	0.717311783
Scrib	1758.758981	2651.013698	0.663428854	0.004542304	0.037380934
Opn4	669.5285805	1009.436762	0.663269464	0.024701566	0.133100329
Numbl	1376.930893	2076.116833	0.663224184	6.79E-06	0.000245743
Gpx1	500.6613328	754.9098083	0.663206819	6.87E-05	0.00145454
Actr1b	1194.834252	1802.074206	0.663032769	1.13E-05	0.000364706
Bmp6	89.80972627	135.4625901	0.66298545	0.032868888	0.163825455
Cst3	620.3703578	935.7475089	0.662967683	4.36E-05	0.001038255
Clic1	634.746009	957.4502937	0.66295453	0.003028944	0.027590342
Smdt1	415.9547037	627.6194655	0.662749845	0.000104733	0.00202561
Tsku	107.6843197	162.5167593	0.6626044	0.041663623	0.194361826
Tmem145	1115.551647	1684.100273	0.662402153	8.98E-06	0.000303055
Barhl2	1232.527208	1860.879605	0.662335814	7.78E-06	0.000272043
Npepl1	484.7332943	731.9316304	0.66226581	7.52E-05	0.001555633
Pom121	1370.371187	2070.091831	0.661985699	1.07E-05	0.000349546
Prr12	2349.718664	3549.794019	0.661930988	3.37E-06	0.000143458
Grik5	2141.337877	3235.433686	0.66183952	3.86E-06	0.000160109
Slco2b1	103.6005048	156.5444611	0.661796042	0.27459215	0.680857066
Lrfn1	994.2636481	1502.478399	0.661749047	0.0002628	0.004153893
Ssna1	445.3587117	673.1041456	0.66164904	8.92E-05	0.001779583
Nrarp	986.7792504	1491.760687	0.661486295	0.12049581	0.407794115
Rps21	310.7531441	469.8080128	0.661447093	0.000227148	0.003688345
Ecm1	95.39594243	144.223906	0.661443342	0.171673926	0.511932728
Scd2	4689.137889	7091.040861	0.661276388	2.98E-06	0.000130338
Tmsb4x	687.2780006	1039.380071	0.661238386	2.89E-05	0.000757044
Cadm4	1553.239655	2349.11858	0.661201043	6.76E-06	0.000245143

Dmrta2	44.67412394	67.57076768	0.661145721	0.064266748	0.262952437
Snrpd2	236.1096657	357.2265003	0.660952268	0.000532949	0.007250732
Fam195b	638.1045265	965.6322144	0.660815284	3.05E-05	0.000785031
Cacna1f	70.4668786	106.6399431	0.660792538	0.201424848	0.565063613
Fn1	1646.300546	2491.853314	0.660673137	0.083548979	0.316709755
Plod1	178.4047299	270.0897877	0.660538599	0.013185596	0.083107276
Atp6v0d1	1163.824979	1762.043985	0.660497121	9.15E-06	0.000307066
Ddx39b	1869.598436	2831.55613	0.660272426	5.42E-06	0.000206938
Tuba1c	102.8976461	155.842448	0.660267131	0.248745482	0.641079943
Gna12	1259.120259	1907.08758	0.660232006	9.91E-06	0.000328166
Mvd	808.6913609	1224.874331	0.660223943	1.51E-05	0.0004619
Prrt3	278.4425436	421.7658669	0.660182735	0.017195809	0.101438056
Irx4	1261.866251	1911.391424	0.66018202	5.40E-06	0.00020666
Dlg4	2320.97566	3516.116292	0.660096387	3.28E-06	0.000141141
Slc1a6	462.2405863	700.2727426	0.660086504	6.91E-05	0.001461211
Csk	1488.739794	2255.563285	0.660030159	5.79E-06	0.000216454
Cenpm	114.5728906	173.5996684	0.659983349	0.097729297	0.353242916
Nrg2	232.8957631	352.93692	0.659879287	0.019068385	0.109702171
Timm13	412.6481833	625.3397008	0.659878435	9.12E-05	0.00181606
Eid2	590.4267921	894.8181642	0.659828796	3.58E-05	0.000895812
Gpc1	1908.565193	2892.943624	0.659731209	4.05E-06	0.000165971
Slc2a6	193.8561028	293.8934311	0.659613596	0.00063974	0.008335796
Ctnnb1	3203.577178	4857.008035	0.659578316	3.32E-06	0.000142016
Actb	2503.562982	3796.201143	0.659491657	4.01E-06	0.000164797
Foxc1	32.33467128	49.03958375	0.6593586	0.467262316	0.885374272
Dazap1	1182.721432	1793.797982	0.659339259	8.97E-06	0.000302897
Ctdsp2	1415.997494	2147.611005	0.659336114	0.034524469	0.169968588
Uchl1	1198.555993	1818.25972	0.659177553	8.26E-06	0.000284831
Hspa8	1039.465243	1577.151249	0.659077716	1.03E-05	0.000337301
Pld4	111.5726738	169.2895406	0.659064189	0.12124855	0.409401064
Ywhag	3727.787352	5656.417457	0.659036816	2.45E-06	0.000112514
Cope	750.4435592	1138.740267	0.659012051	2.09E-05	0.000592595
Rprm	1073.289494	1628.697081	0.658986565	7.73E-06	0.000270852
Calb2	1646.162746	2498.774689	0.658787987	5.13E-06	0.000200004
Onecut1	182.0131567	276.3330798	0.658673065	0.012281819	0.078737066
Sf3b4	353.3262308	536.5200168	0.658551815	0.000147013	0.002651838
Mrps28	150.4240606	228.4344089	0.65850001	0.001987855	0.019823228
Jup	2220.51634	3372.118332	0.658493007	2.59E-06	0.000117137
Ino80b	332.336617	504.7122591	0.658467495	0.000163063	0.002887264
Cend1	1056.694734	1604.78144	0.658466448	6.77E-06	0.00024533
Pim3	677.5837722	1029.143471	0.658395833	2.49E-05	0.000673559
Snord37	12.78274646	19.41662285	0.658340359	0.254354896	0.648487038
Shank3	1616.378921	2455.394455	0.658297048	0.002627521	0.024714914

Tnfaip2	10.74962749	16.33031635	0.658262048	0.489272386	0.90800298
Cdk2ap2	355.4179876	540.0211177	0.65815572	0.000122076	0.002289297
Scand1	246.7890911	375.0015425	0.658101536	0.000385145	0.005608181
Cyp26c1	66.17798832	100.5692599	0.658033959	0.139432886	0.448744071
Med29	152.9918137	232.5019414	0.658023812	0.001908168	0.019223781
Prkaca	1484.190878	2255.781054	0.657949882	5.42E-06	0.000206938
Tle2	199.0050173	302.4847654	0.657900959	0.0214421	0.120177282
Arf1	1405.599359	2136.795589	0.657807123	5.77E-06	0.000216243
Rab1b	1196.128917	1818.409409	0.657788566	7.91E-06	0.000275859
Taf4a	781.9975835	1189.02727	0.657678426	1.56E-05	0.000473244
Thbd	18.66319984	28.38391661	0.657527292	0.704492997	1
Akna	319.7256151	486.2754925	0.657498928	0.001501126	0.016088863
Fbln1	373.317113	567.795658	0.657484973	0.000112816	0.002149022
Npdc1	1414.56982	2151.626149	0.657442196	4.68E-06	0.000187635
Eif5a	1165.884934	1773.760135	0.657295714	8.33E-06	0.000286389
Spata20	11.46832524	17.45071096	0.65718384	0.27602773	0.682391125
Cdt1	470.0891735	715.3435944	0.65715158	0.08194531	0.31264199
Caly	455.5896058	693.4862493	0.6569555	3.93E-05	0.000963052
Zfp444	900.7140803	1371.04284	0.65695546	1.20E-05	0.000380991
Alpl	34.45588345	52.45373784	0.656881375	0.066612382	0.269375313
Sorbs3	132.8623912	202.3103736	0.656725549	0.004094285	0.03464073
Zfp574	997.6578006	1519.419997	0.656604364	9.83E-06	0.000326682
Nfatc4	156.8222847	238.8990317	0.656437507	0.001443673	0.015580149
Evpl	125.8801344	191.7712597	0.656407715	0.09179383	0.337727932
Ppp2r5b	1798.502189	2739.964854	0.65639608	3.02E-06	0.000131544
Hras	719.703196	1096.629226	0.656286718	1.64E-05	0.000491031
Arap1	277.8138446	423.3643195	0.656205145	0.001091196	0.012574598
Crybb3	21.63595152	32.97449983	0.656141916	0.179272299	0.526829688
Llgl1	1359.279831	2071.8328	0.656076027	0.014349262	0.088464317
Mrps12	277.6090628	423.1378799	0.656072349	0.000241805	0.003881179
Pdlim4	65.04719009	99.14924713	0.656053293	0.03289141	0.163873112
Hspbp1	719.4369049	1096.712417	0.655994128	1.64E-05	0.000491031
Crmp1	3787.569589	5774.588063	0.655902992	1.85E-06	9.03E-05
Tmbim6	1668.70657	2544.877783	0.65571187	4.54E-06	0.00018336
Agap3	1711.020134	2609.859269	0.655598619	3.88E-06	0.00016052
Slc25a4	1430.298669	2181.714472	0.655584719	5.09E-06	0.000198674
Phc2	1470.265475	2243.607434	0.655313159	0.003324517	0.029561668
Rpl19	277.7840341	423.9569474	0.655217554	0.000235086	0.003794005
Csnk1g2	1311.014585	2000.894834	0.655214139	5.70E-06	0.000214074
Vps37d	573.7559851	875.69602	0.655199946	2.29E-05	0.000631132
Lrp3	1542.609943	2354.764416	0.655101603	3.44E-06	0.00014621
Arl10	515.5548163	787.1271924	0.654982856	3.19E-05	0.0008168
Gadd45gip1	209.6642466	320.1065735	0.654982634	0.000552363	0.00742721

Fau	340.4647975	519.8487202	0.654930529	0.000119722	0.002254642
Fbxw4	265.8364447	406.0045381	0.654762249	0.000271323	0.004264619
Fbrsl1	1603.994512	2450.149393	0.654651719	2.96E-06	0.000129705
Tmem88	19.7412259	30.16442604	0.654453888	0.16232044	0.494385461
Srf	2102.72919	3213.3878	0.654365212	0.044626262	0.203878352
Cntfr	887.6776963	1356.692967	0.6542952	9.86E-06	0.000326726
Pipox	85.00377881	129.9205636	0.654275016	0.333952696	0.754852885
Cd52	19.20937778	29.36171126	0.654232228	0.24983604	0.642309791
Itgb5	693.4341508	1059.923471	0.654230395	0.054642864	0.234586964
Ncdn	1986.9081	3037.637568	0.6540965	4.79E-06	0.000190018
Paqr4	578.1332826	883.9368133	0.654043676	2.31E-05	0.000635775
Csf1r	718.490627	1098.814021	0.653878284	0.052825138	0.229136488
Flnc	96.088599	146.9635405	0.653826103	0.513386181	0.931883076
Rtn1	3092.408936	4730.174866	0.653762075	1.62E-06	8.09E-05
Pebp1	403.48595	617.2237422	0.653711	6.41E-05	0.00138063
Tpt1	595.9430791	911.6426099	0.653702528	2.35E-05	0.000641827
Mri1	339.074602	518.801462	0.65357295	8.48E-05	0.001713029
B4galnt3	20.63906374	31.58093677	0.653529181	0.149941928	0.469128044
Olig2	167.0355811	255.6013392	0.653500415	0.262259586	0.661517983
Adrm1	582.195267	891.1668665	0.653295459	2.17E-05	0.000610031
Sag	398.2219589	609.5858988	0.653266356	0.000311107	0.004751911
Actg1	581.8298848	890.823104	0.653137399	2.34E-05	0.000641827
Tmem107	142.6428689	218.4093214	0.653098815	0.105941405	0.373060066
Furin	1094.913588	1676.51009	0.653090962	6.18E-06	0.000228343
Phf1	635.5137712	973.1332758	0.653059336	1.40E-05	0.000433493
Tob2	1532.917223	2347.438292	0.653017048	0.000104383	0.002020306
Gpr135	185.0022902	283.324464	0.6529697	0.011033511	0.072849682
Calhm2	11.45319968	17.54046786	0.652958619	0.3017772	0.714628315
Shmt2	876.5475405	1342.514614	0.652914711	0.029827769	0.152811843
Naglu	281.7471997	431.7364304	0.652590747	0.000197096	0.003322445
Ddr2	31.00298712	47.50962001	0.652562305	0.692446461	1
Crybb1	28.99482008	44.4397513	0.652452348	0.303192516	0.716556065
Hmox1	179.0506476	274.4352332	0.652433164	0.211639884	0.581409505
Dvl3	1387.057646	2126.079503	0.652401589	3.21E-06	0.000138372
Ksr1	699.613032	1072.383478	0.652390722	1.44E-05	0.000444617
Nfatc1	32.86574422	50.38757638	0.652258882	0.167049731	0.503408418
Rps13	337.1317547	516.9179351	0.652195894	9.83E-05	0.001922835
Ggn	49.30338276	75.60386061	0.652127846	0.027580196	0.144215438
Pafah1b3	843.657285	1293.702685	0.652126099	8.84E-06	0.000299378
Nptxr	2639.273054	4047.315851	0.652104543	1.73E-06	8.51E-05
Polr2e	497.7549079	763.3708848	0.652048588	3.58E-05	0.000896337
Crx	1237.203581	1897.453604	0.65203364	0.001513992	0.016207112
Mxra8	76.67150626	117.593964	0.652002056	0.157540804	0.484949147

Npb	10.36080268	15.89467883	0.651840958	0.271278019	0.676385631
Tprn	426.1779936	653.8723605	0.651775514	0.02583712	0.137486869
Kank2	147.9377803	227.0067278	0.651688969	0.02025532	0.114861004
Pecam1	49.55907231	76.05402908	0.651629807	0.341012821	0.761428716
Eef2	3858.382408	5921.722814	0.65156417	1.46E-06	7.53E-05
Adap1	1322.637498	2029.955543	0.651559835	2.62E-06	0.00011796
Rps27	187.958093	288.508592	0.651481787	0.000675662	0.008701237
Syngr3	1001.239035	1537.116452	0.651374873	4.49E-06	0.000181706
Myl12b	771.9860124	1185.187942	0.651361683	1.01E-05	0.000332156
Dbn1	2957.499663	4541.127213	0.651269943	1.48E-06	7.60E-05
Gpx3	225.4060636	346.1967449	0.651092383	0.001106781	0.012710011
Fam134a	1726.193398	2651.558651	0.65101083	2.48E-06	0.000113556
Dlx3	54.58947487	83.85513223	0.650997422	0.036013495	0.1750885
Eif4h	1877.164685	2884.108775	0.650864732	2.59E-06	0.000117037
Prex1	949.909696	1459.752995	0.650733171	0.061356999	0.254527876
Rnf5	363.543382	558.6792266	0.650719348	6.80E-05	0.001446631
Exosc5	208.2422702	320.0822418	0.650589889	0.009181715	0.0635506
Cited2	1219.95756	1875.360315	0.650519023	3.91E-06	0.000161356
Acot7	1303.261948	2003.658249	0.650441236	3.03E-06	0.000132053
Rps25	208.0316638	319.8508311	0.650402136	0.000409983	0.005885821
Rps15	508.8766849	782.426037	0.650383117	2.68E-05	0.000715281
Vasp	874.1739271	1344.136072	0.650361184	6.67E-06	0.000242264
Pcbp3	1707.987989	2626.327101	0.650333307	2.26E-06	0.000106197
Postn	330.6761428	508.5338755	0.650253914	0.629877289	1
Csnk2b	631.024162	970.5619276	0.650163729	1.59E-05	0.00048071
Ltbr	283.4603338	436.0304168	0.650093028	0.000180321	0.003116937
Fbll1	414.9088986	638.2423068	0.650080532	3.76E-05	0.000931434
Il10ra	30.74620832	47.30059219	0.650017408	0.085123899	0.320459548
Ttll11	393.7134959	605.7215204	0.649990933	5.55E-05	0.001240708
Lzts2	967.4389158	1488.500418	0.649941985	6.01E-06	0.000223272
Gnb2l1	1259.934507	1938.741144	0.649872476	4.39E-06	0.000177793
Maged1	2624.516394	4039.192322	0.649762672	1.54E-06	7.77E-05
Puf60	1315.213832	2024.149851	0.649761099	3.57E-06	0.000150172
Fbxo24	13.37708717	20.59305198	0.649592259	0.233141718	0.617087822
Scap	1250.642324	1925.880112	0.649387424	3.16E-06	0.000136373
Arhgdig	493.3867026	759.8530505	0.649318579	2.06E-05	0.000586869
Ctsd	1607.19381	2475.778167	0.649167131	3.14E-06	0.000135645
Gtpbp6	294.28802	453.3364525	0.649160283	9.63E-05	0.001889087
Zcchc24	143.1451351	220.5237219	0.649114453	0.02715516	0.142724724
Mzb1	11.22947864	17.29970857	0.649113746	0.27481835	0.68097477
Mrpl4	363.1868398	559.6015793	0.649009676	6.84E-05	0.001452226
Cdc34	464.9769634	716.6156269	0.648851275	0.004144266	0.034963209
Rnf10	2407.211339	3709.968469	0.648849541	1.47E-06	7.57E-05

Ndufb6	215.6153427	332.3536823	0.648752682	0.000355562	0.005275932
Pip5k1c	3112.012022	4796.925183	0.648751419	9.36E-07	5.41E-05
Psd	1965.281033	3029.614035	0.648690232	1.20E-06	6.43E-05
Ebf4	367.2576871	566.1719277	0.648668133	5.09E-05	0.001163292
Tmub1	327.1477309	504.4544129	0.648517929	7.35E-05	0.001529468
Sox9	1254.157399	1934.085799	0.648449722	0.009463	0.064932025
Inppl1	658.3388646	1015.297993	0.64841935	0.005838417	0.045187232
Pnoc	132.4039718	204.2223412	0.648332455	0.001453145	0.015675994
Lmcd1	12.73460425	19.64522347	0.648229035	0.254287612	0.648487038
Cox11	125.7665871	194.0698767	0.648047957	0.002153379	0.021124308
Nenf	268.5778069	414.5167066	0.647929993	0.000154005	0.002761046
Dock6	230.0721733	355.2700357	0.647598025	0.074288169	0.291308059
Ndfip1	981.9867933	1516.711768	0.647444567	4.87E-06	0.000191806
Men1	1012.604414	1564.04662	0.647425979	4.56E-06	0.000183595
G0s2	15.38521309	23.7645964	0.647400563	0.218415254	0.593361012
Gapdh	507.9287145	784.7774405	0.647226447	2.10E-05	0.000595241
Hps6	112.3118817	173.5829772	0.647021289	0.009520146	0.065194944
Cabp1	689.0590118	1065.635325	0.646618028	8.93E-06	0.000302061
Get4	510.0274262	789.07709	0.64635944	2.19E-05	0.000611712
H2afz	432.6160839	669.461151	0.646215368	0.000690354	0.008857713
Slc6a1	3235.840118	5007.443305	0.646206042	8.04E-07	4.89E-05
Ube2m	1014.483427	1570.000249	0.646167686	3.97E-06	0.000163395
Arhgap23	2139.592897	3311.425171	0.646124489	9.38E-07	5.41E-05
Tbkbp1	810.7605356	1254.917821	0.646066637	4.54E-06	0.00018336
Galk1	298.703237	462.34138	0.646066413	0.001601253	0.016935184
Vps37b	1086.642175	1682.264914	0.645939986	0.000397563	0.005744839
Sox8	980.2751679	1517.706621	0.645892397	0.065731428	0.267075473
Notum	27.68287087	42.8639208	0.645831514	0.372876363	0.796816745
Nudt3	1776.78091	2751.246934	0.645809319	1.72E-06	8.47E-05
Ehd2	692.8122192	1073.173977	0.64557307	0.00012038	0.002263836
Rsph3b	49.4241991	76.58333555	0.645364931	0.025908594	0.137811891
Irf5	79.14574053	122.6528902	0.645282312	0.106263231	0.373845804
Syp	1316.153612	2039.807767	0.645234141	2.12E-06	0.000100655
Nnat	1470.735753	2279.47243	0.645208836	2.22E-06	0.000104561
Vstm2b	295.8066154	458.4759562	0.645195482	8.87E-05	0.001771246
Gstm1	230.591996	357.4593236	0.645085974	0.000251575	0.004012171
Rbpms2	1283.626601	1990.089231	0.645009571	1.88E-06	9.15E-05
Pou4f1	2704.041198	4192.920356	0.644906406	8.27E-07	4.95E-05
F8a	351.1206126	544.552757	0.644787136	5.07E-05	0.001160094
Fam104a	350.7205375	543.9442285	0.644772973	5.82E-05	0.001285394
Xrcc1	590.9007571	916.6406863	0.644637278	0.000114325	0.002173077
Ncor2	3032.820588	4706.652864	0.64436887	7.68E-07	4.72E-05
Tek	47.53829283	73.78152268	0.644311626	0.532617625	0.948608875

Cav1	33.69541139	52.31617876	0.644072488	0.692158485	1
Tpi1	1001.576617	1555.213481	0.644012304	3.68E-06	0.000154309
Kdm6b	2056.547699	3193.869517	0.643904733	9.83E-07	5.56E-05
Ppp1ca	1076.337958	1671.656844	0.643874945	3.35E-06	0.000143102
Bsg	1106.360692	1718.296023	0.643870834	3.14E-06	0.000135645
Dda1	1132.480114	1759.141133	0.643768765	2.43E-06	0.000112001
Fkbp8	1473.047147	2288.250212	0.643743914	1.90E-06	9.19E-05
Cyc1	784.4648006	1218.606778	0.643739075	6.14E-06	0.000227524
Rnf44	2254.30791	3503.001552	0.643536087	8.79E-07	5.18E-05
Gaa	1447.706755	2249.640032	0.64352818	1.21E-06	6.45E-05
Mta1	1502.626167	2335.554674	0.643370153	1.68E-06	8.27E-05
Tppp3	815.1830628	1267.209736	0.643289772	4.92E-06	0.000193647
Rps23	212.2080754	329.9197802	0.643211132	0.000286805	0.004462562
Mgat4b	1374.598131	2137.753811	0.643010492	1.50E-06	7.63E-05
Dad1	418.4831135	650.8405494	0.642988692	2.66E-05	0.000710999
Sema6c	1546.19746	2404.944123	0.642924484	1.09E-06	5.95E-05
Fxyd6	1216.160673	1891.813489	0.642854425	2.16E-06	0.000102289
Psenen	144.539773	224.8606244	0.64279717	0.001134855	0.012963498
Psap	2213.384753	3443.81546	0.642712938	1.08E-06	5.93E-05
Slc12a5	2127.401787	3310.712636	0.642581227	0.011614047	0.075375458
Mrpl12	264.9091515	412.2836421	0.642541019	0.000140069	0.002559707
Gli1	557.0326994	867.0042094	0.64247981	0.308207147	0.722775334
Ppia	375.3900159	584.4194927	0.642329732	0.000471175	0.006589492
Kctd17	851.9277342	1326.401446	0.642284986	3.33E-06	0.000142256
Tgfbr2	144.5967751	225.1351616	0.642266513	0.518191575	0.937099613
Spint2	572.8904093	892.0569516	0.642212819	1.19E-05	0.000379897
Rps6	332.2765964	517.4788172	0.642106663	5.44E-05	0.001218594
Drap1	671.7546877	1046.515185	0.641896742	7.45E-06	0.000263497
Ddah2	1165.937686	1816.446674	0.64187829	2.50E-06	0.000113687
Gadd45g	281.8599719	439.173577	0.641796289	0.054083651	0.233146168
Vim	1235.002886	1924.307034	0.641790974	5.51E-06	0.000209328
Pianp	809.9686172	1262.315639	0.641653001	0.004830882	0.039125131
Snrpb	828.6708053	1291.478601	0.641645014	5.08E-06	0.000198536
Setd1b	1489.331934	2321.315782	0.641589544	9.88E-07	5.57E-05
Mocs3	229.4164292	357.754762	0.641267297	0.000175671	0.003054486
Cacnb3	1966.320617	3066.827433	0.641157894	7.94E-07	4.84E-05
Rps7	320.7805126	500.4518957	0.640981711	6.23E-05	0.001355331
Mtss1l	3227.339809	5035.623447	0.640901736	4.16E-07	2.89E-05
Crocc	1118.687618	1745.554024	0.640878256	0.003247424	0.029090776
Fen1	39.76558585	62.05644772	0.640797005	0.076949243	0.298563963
Igf2os	22.0514662	34.41392965	0.640771525	0.119914378	0.406345001
Fbxl16	1751.828947	2734.176448	0.640715396	8.23E-07	4.94E-05
Tecr	1205.721016	1881.899116	0.640693758	1.87E-06	9.10E-05

Fcgrt	97.24057601	151.8170394	0.640511608	0.605439943	0.988955325
Zfp524	63.88597373	99.75050304	0.64045766	0.012983728	0.08210745
Rtn2	1159.663525	1810.812603	0.640410567	1.92E-06	9.24E-05
Ptp4a3	787.2342149	1229.388629	0.640346101	4.14E-06	0.000169114
Whrn	671.8893939	1049.337771	0.640298493	4.93E-06	0.000193647
Tpgs1	457.3588851	714.5793472	0.640039328	1.71E-05	0.00050422
n-R5-8s1	48.80809198	76.29279746	0.639747048	0.023739236	0.129493846
Tesk1	930.7663315	1455.089857	0.639662442	2.43E-06	0.000112001
Rpl7a	698.563331	1092.122963	0.639637984	5.64E-06	0.000213126
Csdc2	1931.391103	3019.609	0.639616289	6.41E-07	4.12E-05
Akt1	2223.348813	3476.24152	0.639584103	7.42E-07	4.62E-05
Mt3	86.34384176	135.0057702	0.639556677	0.004173379	0.035147293
Slc25a10	301.093274	470.8080488	0.639524483	0.000220778	0.003611438
Atp13a2	2623.861343	4102.97052	0.63950285	5.30E-07	3.54E-05
Trip10	227.0646876	355.1155598	0.639410697	0.000195005	0.003302127
Tmem173	15.54399009	24.31018486	0.639402381	0.309323048	0.724176957
Erh	105.006707	164.2332132	0.639375586	0.002799241	0.025919682
Ptn	620.1125309	970.1328707	0.639203711	6.96E-06	0.000249808
B4galnt1	1169.200588	1829.241999	0.639172175	1.21E-06	6.46E-05
Rplp0	972.032833	1520.917805	0.639109378	2.75E-06	0.000122008
Dalrd3	346.9938319	543.0261405	0.639000236	0.017561941	0.103060868
Rpl36	240.0581875	375.7861392	0.638815971	0.000139008	0.002553207
Bag6	3065.983672	4799.70769	0.638785499	4.98E-07	3.37E-05
Gstt1	18.39378804	28.79594307	0.638763176	0.146100385	0.460915861
Rell2	346.8385102	543.0186278	0.638723043	2.55E-05	0.000688657
Fignl2	308.2423525	482.6060625	0.638703855	0.009472403	0.064948032
Pamr1	58.34796146	91.35571292	0.638689794	0.611605082	0.994021485
Itga5	94.43299293	147.856168	0.638681458	0.095239077	0.346890022
Ung	202.9688316	317.8275994	0.638612984	0.205270746	0.571619312
Igsf9	1375.188702	2153.508319	0.638580632	0.045465647	0.206503444
Cic	3587.952863	5618.643673	0.638579891	3.59E-07	2.61E-05
Kctd13	842.2762434	1319.174203	0.638487503	2.58E-06	0.000116793
Ankrd34a	450.0533963	705.0005033	0.638373156	0.026756643	0.141161786
Frat2	759.1167019	1189.298616	0.638289402	0.084430476	0.318866046
Efna2	722.8296953	1132.799729	0.638091338	4.82E-06	0.00019091
Foxd1	253.0870495	396.7551223	0.63789233	0.300298699	0.712536782
Plekhh3	526.9787071	826.2070574	0.637828862	1.06E-05	0.000344566
Tinagl1	24.09369802	37.7782199	0.637766895	0.391049383	0.821051262
Tspo	41.39399981	64.90649961	0.637748146	0.065699909	0.267029242
Wbp2	1294.37713	2029.986991	0.637628288	1.20E-06	6.43E-05
Ptpn18	16.74423581	26.26424869	0.637529594	0.341421579	0.761920098
Cfl1	1231.386522	1931.64252	0.637481578	1.52E-06	7.69E-05
Trim8	1747.79114	2741.847872	0.637450078	7.33E-07	4.58E-05

Map2k2	1195.230786	1875.295328	0.63735603	1.39E-06	7.24E-05
Shf	607.042775	952.5045385	0.637312213	1.49E-05	0.00045699
Rpl8	873.9167283	1371.764708	0.637074801	2.92E-06	0.000127809
Bai1	1216.92571	1910.45978	0.636980544	9.44E-07	5.43E-05
Ndufs5	74.97318369	117.7537645	0.636694581	0.006504396	0.049150916
Dvl2	511.2525179	803.3103891	0.6364321	0.021241462	0.119254416
Cd81	936.1506778	1471.097706	0.636362	2.28E-06	0.000106686
Ubc	361.9595696	568.9133983	0.636229645	2.66E-05	0.000710999
Klf16	553.3101389	869.7567379	0.636166545	7.05E-06	0.000251888
Dpm3	113.3613741	178.247218	0.635978364	0.00201263	0.020032621
Rps16	259.715489	408.3900454	0.635949607	9.28E-05	0.001835246
Col16a1	82.53547188	129.8040596	0.635846615	0.574846869	0.980234056
Thra	2501.776713	3935.535711	0.635688988	4.43E-07	3.06E-05
Otud1	458.5693535	721.4962532	0.63558106	0.005367443	0.042383296
Cd93	46.82269522	73.67294143	0.635548063	0.634403159	1
Polr2j	210.2852772	330.8864188	0.635521029	0.000196125	0.003315249
Angptl2	142.3300271	224.0956703	0.635130643	0.110851571	0.384982354
Adamts12	36.15636397	56.92793002	0.635125218	0.460744469	0.88015336
Sdc3	3241.298354	5103.456255	0.635118279	2.58E-07	2.03E-05
Glrx5	500.7059343	788.7635621	0.63479851	9.17E-06	0.000307341
Ppdpf	560.8238886	883.5422837	0.634744821	6.81E-06	0.000245743
Ywhaz	1598.284546	2518.797468	0.6345427	6.49E-07	4.16E-05
Rxrb	992.9639349	1565.000605	0.634481502	1.79E-06	8.75E-05
Tubb4a	2232.589942	3518.951064	0.634447567	4.10E-07	2.88E-05
Celf3	1635.020859	2577.766819	0.63427803	6.93E-07	4.40E-05
Ubald1	842.624028	1328.524277	0.634255649	0.004361938	0.036280158
Atf6b	1219.731452	1923.591041	0.634090836	1.09E-06	5.95E-05
Tmem121	315.9415485	498.416421	0.633890729	3.71E-05	0.000919125
Crym	615.1783716	970.4958661	0.633880466	0.005746432	0.044644705
Mrpl34	303.7347905	479.2579835	0.633760524	4.88E-05	0.001130783
Mmp15	1516.609306	2393.099126	0.633742785	6.47E-07	4.15E-05
Aprt	349.6407735	551.7233301	0.63372483	2.61E-05	0.000701804
Rpl14	532.0510927	839.6347895	0.633669661	7.38E-06	0.000261581
Tsen34	731.7328658	1154.962666	0.633555427	2.82E-06	0.00012387
Rsph3a	62.62493225	98.87852171	0.63335223	0.011111991	0.073076774
Mycn	1337.199559	2111.400455	0.633323515	9.75E-07	5.55E-05
Rlbp1	357.9687713	565.2464957	0.633296755	0.072186856	0.285049393
Prr24	593.7669163	937.7892401	0.633156034	4.29E-06	0.000174608
Atn1	2816.79411	4449.180356	0.633104052	2.46E-07	1.96E-05
Irx5	1088.872745	1719.933	0.633090211	1.07E-06	5.92E-05
Map1s	940.5954331	1486.7453	0.632654049	1.12E-06	6.06E-05
Metrnl	28.94391236	45.75358372	0.632604268	0.152802499	0.475218281
Tubg1	577.1799071	912.4655609	0.632549799	1.56E-05	0.000473946

Islr2	4310.212227	6815.078079	0.632452362	2.05E-07	1.69E-05
Vgf	1145.510547	1811.511963	0.632350528	7.84E-07	4.79E-05
Prdm8	475.0704062	751.3050129	0.632326949	8.83E-06	0.000299378
Mfge8	682.9367781	1080.42637	0.632099324	0.038534828	0.183742044
Grin1	1013.420547	1603.43582	0.632030627	0.006280562	0.047800055
Ybx3	1140.469399	1805.236331	0.631756285	1.03E-06	5.71E-05
Rhbdl3	686.6808526	1087.330611	0.631529036	0.064722775	0.264395178
Pdlim7	640.4891383	1014.229397	0.631503228	5.98E-05	0.001311351
Prcc	954.2979097	1511.330474	0.631429013	1.49E-06	7.62E-05
Wsb2	1289.849079	2042.961618	0.631362365	6.17E-07	3.99E-05
Prrc2a	4669.850726	7396.810766	0.631332999	1.74E-07	1.50E-05
Atp9a	3125.122776	4951.860133	0.631100777	2.24E-07	1.82E-05
Proser2	280.6014229	444.6480744	0.631064069	0.000326249	0.004937687
Cdh22	305.2280378	483.8405592	0.630844256	0.054413946	0.234037341
Spsb3	234.5107277	371.9176083	0.630544837	9.96E-05	0.00194167
Ltbp3	427.0124789	677.212588	0.630544214	1.12E-05	0.000362604
Rbm38	255.2997902	404.90647	0.630515463	0.005607311	0.04388532
Hnrnpf	291.4825094	462.5891013	0.630111061	4.85E-05	0.001124381
Nt5dc2	1116.975174	1772.728933	0.630087969	8.73E-07	5.18E-05
Pfn1	813.8865532	1292.034894	0.62992614	1.99E-06	9.52E-05
Wdr6	3014.901896	4786.563153	0.629867778	1.94E-07	1.63E-05
Capn6	145.2337681	230.5845781	0.62985031	0.655332134	1
Sap30l	374.0014113	593.7943082	0.629850112	8.58E-05	0.001726805
Lyl1	39.69877737	63.02996325	0.629839767	0.165698429	0.500873182
Impdh1	1151.031569	1827.702884	0.629769521	8.18E-07	4.92E-05
Palm	2131.477602	3384.688571	0.629741129	2.67E-07	2.07E-05
Doc2b	415.496786	659.8531595	0.62968068	2.29E-05	0.000631132
Asic4	208.872079	331.7134931	0.629676161	0.010375722	0.069564642
Fbxl15	199.6954954	317.2551519	0.629447605	0.000157963	0.002809217
Prkcdbp	107.3538187	170.5905366	0.629306999	0.001555826	0.016528162
Sox13	451.1221624	716.8864224	0.629279825	0.189406034	0.542891622
Igfbp3	211.3323797	335.8557419	0.629235572	0.354274332	0.778030328
Fos	1887.046138	2999.042976	0.629216105	3.84E-07	2.75E-05
Irf2bp1	1104.908171	1756.045153	0.629202597	8.53E-07	5.10E-05
Srebf2	3593.631907	5712.126566	0.629123299	1.71E-07	1.49E-05
Zmiz2	2831.427609	4501.68705	0.628970334	1.72E-07	1.50E-05
Mbd3	1030.555222	1638.982344	0.628777501	1.10E-06	5.99E-05
Ppp2r1a	2249.254695	3577.664756	0.628693533	2.51E-07	1.99E-05
Lemd2	1040.488987	1655.523991	0.628495264	8.79E-07	5.18E-05
Neurl1a	616.694768	981.5201997	0.628305732	2.41E-06	0.000111518
Ppib	251.1594543	399.7936273	0.628222756	7.21E-05	0.001509115
Ttyh1	1181.329163	1880.585579	0.628170915	0.03757533	0.180171329
Gltscr1	684.3409052	1089.48016	0.628135261	1.79E-06	8.75E-05

Cenpb	1411.690587	2247.475414	0.628122816	5.87E-07	3.83E-05
Nat6	169.7767757	270.3111586	0.62807905	0.000251631	0.004012171
Capns1	1021.426026	1627.006088	0.62779484	6.76E-07	4.31E-05
Mdh2	1100.583478	1753.102335	0.627791918	7.59E-07	4.68E-05
Rpl27	324.7623445	517.3761954	0.627710257	2.72E-05	0.000724652
Sirt6	458.0346576	730.0728451	0.627382131	7.01E-06	0.000250975
Map7d1	2310.730877	3683.948836	0.627242934	1.79E-07	1.54E-05
Igfbp5	1327.918801	2117.69082	0.627059809	0.415097483	0.849677696
Fam222a	804.5031121	1283.131628	0.626984087	1.47E-06	7.56E-05
Lypla2	922.9917395	1472.316599	0.626897598	9.32E-07	5.41E-05
Pitpnm1	3058.466259	4879.385538	0.626813814	1.70E-07	1.49E-05
Btbd2	1358.547286	2167.92849	0.626656872	3.64E-07	2.63E-05
Lmtk3	2756.377353	4398.57823	0.626651888	1.51E-07	1.37E-05
Abcc3	57.17821926	91.25561915	0.626572038	0.084656105	0.31944518
Hnrnpa0	2336.473854	3729.775907	0.626438132	2.40E-07	1.94E-05
Slc29a4	1796.062836	2867.794869	0.626287066	1.97E-07	1.65E-05
Ier2	894.4301576	1428.26865	0.626233837	0.013949167	0.08664233
Hmgn2	625.6661709	999.107113	0.626225319	0.001429878	0.015471265
Slc27a1	586.6550444	936.8402963	0.626206032	0.002346553	0.022543916
Pkn3	90.46869695	144.4790978	0.626171524	0.123661223	0.415010884
Arhgdia	1554.429761	2482.683155	0.626108796	3.35E-07	2.47E-05
Prdm13	735.2557325	1174.568476	0.625979453	0.02976287	0.152581951
Tulp1	644.1125018	1029.079556	0.62591128	0.008078288	0.058034105
Kcnk12	36.32777396	58.05116332	0.625788906	0.157047419	0.483799497
Podxl2	1378.856121	2203.59574	0.625730072	4.07E-07	2.88E-05
Zfp428	656.269943	1048.935813	0.6256531	1.91E-06	9.22E-05
Gpr153	2426.564703	3878.748659	0.625605038	1.26E-07	1.20E-05
Gpr124	382.1973014	611.085797	0.625439674	0.00135147	0.014820338
Sgta	1289.235884	2061.329258	0.625439084	4.65E-07	3.19E-05
Piezo1	488.3953294	780.903091	0.625423737	0.034983306	0.171368433
Olfm1	3203.68488	5122.832349	0.625373751	1.15E-07	1.13E-05
Drd4	20.94641564	33.49431622	0.625372242	0.107378107	0.376519161
Pdxp	471.0116903	753.2243661	0.625327209	5.01E-06	0.000196305
Pax2	40.93034291	65.48826776	0.625002681	0.024676818	0.133048089
Tmem160	284.8435538	455.7515868	0.624997393	3.33E-05	0.000847081
Ramp2	59.04763776	94.490485	0.624905648	0.162088371	0.494015546
Rap1gap	1770.205704	2833.026713	0.624846104	1.66E-07	1.48E-05
Ap2a1	1638.851098	2623.616176	0.624653527	2.12E-07	1.74E-05
Dyrk1b	406.6127229	650.9819069	0.624614476	8.71E-06	0.000297122
Endog	62.53080578	100.1433381	0.624413036	0.007932634	0.057267007
Inf2	44.06604043	70.62885908	0.623909844	0.021312878	0.119528821
Rpl10a	597.7103476	958.2742194	0.623736229	2.40E-06	0.000111518
Hcn4	1193.150174	1913.208692	0.623638278	2.73E-07	2.10E-05

Laptm5	379.7602931	609.0541207	0.623524709	0.037438629	0.17972824
Eno1	809.2026789	1297.82371	0.623507394	1.01E-06	5.66E-05
Rpsa	518.3839371	831.4673718	0.62345674	3.58E-06	0.000150172
P2ry6	40.9777305	65.74361446	0.623295978	0.269329073	0.673527902
Trim28	2638.541054	4234.110428	0.623163023	1.50E-07	1.37E-05
Mlf2	1416.728426	2273.483765	0.623153087	2.96E-07	2.25E-05
Sema6b	891.2720425	1430.76781	0.622932691	5.04E-07	3.40E-05
Rpl13	385.2882921	618.5894716	0.622849741	9.24E-06	0.000308461
Fcna	26.87423604	43.14772245	0.622842517	0.687652076	1
Pold2	480.5605698	771.6678541	0.62275572	0.00803189	0.057763381
Tubb3	1976.532739	3174.792165	0.62257075	1.81E-07	1.54E-05
Ptbp1	1128.04003	1812.097528	0.622505142	0.007773276	0.056485664
Amh	54.25264703	87.174262	0.622347076	0.028639748	0.148583751
Psmb5	229.6528038	369.1152592	0.622170983	7.14E-05	0.001497976
Pak4	401.9666205	646.0810294	0.622161311	0.006968487	0.051845231
Ankrd13b	2228.232505	3581.453967	0.622158633	1.09E-07	1.09E-05
Srm	574.9157981	924.1085472	0.62213016	2.22E-06	0.000104561
Nfatc2	44.13873512	70.95104359	0.622101281	0.067117782	0.270758421
Kcnj11	94.88198538	152.5253663	0.62207348	0.001541407	0.016421752
Mob3a	216.3189354	347.7828425	0.621994271	6.57E-05	0.001405665
Cspg5	1545.418847	2485.341238	0.621813546	2.62E-07	2.05E-05
Aplp1	2602.513069	4186.458433	0.621650283	1.17E-07	1.14E-05
Aldoa	1371.535841	2206.787026	0.621508023	2.67E-07	2.07E-05
Mtfp1	159.3207573	256.4537763	0.621245511	0.013497063	0.084547159
Zyx	1023.139789	1647.266039	0.621113873	6.84E-06	0.000246167
Hpca	1302.911398	2097.754106	0.621098247	2.05E-07	1.69E-05
Ptov1	1864.594116	3002.72369	0.620967598	1.18E-05	0.000379341
Syngr2	94.84679024	152.805539	0.620702567	0.152783258	0.475218281
Sbk1	4144.935012	6678.100296	0.620675765	6.90E-08	7.50E-06
Tsc22d4	344.9345452	555.9349172	0.620458501	1.14E-05	0.000366737
Uqcrq	312.6143714	503.9839368	0.620286379	1.73E-05	0.00050851
RP23-448C3.1	23.32268923	37.6056681	0.620190796	0.073301834	0.288482408
Vat1	1289.802739	2079.80743	0.620154886	2.50E-07	1.99E-05
Pigt	1291.980011	2083.999571	0.619952148	2.60E-07	2.04E-05
Chst14	39.37816435	63.53763526	0.619761252	0.02762653	0.144388038
Lingo1	2225.983081	3592.058563	0.619695654	7.49E-08	7.94E-06
Tmem198	694.0735753	1120.252946	0.619568623	9.21E-07	5.37E-05
Aqp11	123.3292036	199.1036099	0.619422238	0.001484907	0.015947259
Cited1	76.88471113	124.1386675	0.619345388	0.059342911	0.24889855
Adra1d	55.34368183	89.36356354	0.619309254	0.154195702	0.47798144
Otop3	128.1301865	206.9955712	0.618999652	0.239445186	0.628004802
Tnf	23.43856154	37.86735557	0.618964836	0.597914325	0.988955325
Rara	1122.360504	1813.406207	0.618923934	2.51E-07	1.99E-05

Neurog2	426.9451476	689.8337643	0.618910192	0.210703924	0.579807518
Loxl3	69.27002652	111.9443874	0.618789634	0.029692191	0.152447652
Ccnd1	2506.116407	4050.315194	0.61874602	0.009271956	0.064024791
Sepw1	644.2977163	1041.410689	0.618677841	1.15E-06	6.24E-05
Ntng2	470.031499	759.7370411	0.61867656	0.011182566	0.073358963
Pgls	593.3879795	959.1676937	0.618648838	1.58E-06	7.93E-05
Cotl1	973.2171986	1573.14862	0.618642884	4.58E-07	3.15E-05
Dcakd	1041.794002	1684.673991	0.618395018	3.40E-07	2.49E-05
Iglon5	1440.574437	2329.574228	0.618385291	1.42E-07	1.32E-05
Ypel3	612.8837385	991.1363675	0.618364696	0.0008923	0.010769865
Dll4	371.3288273	600.5864217	0.618277094	0.216729569	0.59099149
Cacna2d4	327.7610725	530.17135	0.618217247	1.40E-05	0.00043205
Pear1	30.92396565	50.02532872	0.618166166	0.391101358	0.821051262
Rpl34	294.5279558	476.5540336	0.618036854	1.93E-05	0.00055569
Tusc5	408.2061909	660.5163247	0.618010753	0.172660794	0.513837059
Clstn1	4250.247477	6885.715227	0.617255773	4.78E-08	5.71E-06
Sf3a2	733.8579739	1189.12341	0.617141979	1.03E-06	5.75E-05
Josd2	251.625228	407.7571996	0.617095733	0.010049533	0.067960811
Prelid1	595.8225769	965.701763	0.616984042	1.42E-06	7.35E-05
Ttc9b	446.4381411	723.6924753	0.616889295	2.76E-06	0.000122008
Ecscr	12.64326385	20.50152975	0.616698559	0.209376752	0.577607493
Lrfn4	1409.488803	2285.688623	0.616658275	1.59E-07	1.44E-05
Lrrc73	324.3243088	526.5300221	0.615965463	8.19E-06	0.000283617
Trim14	12.76623814	20.73464934	0.615695878	0.171472008	0.511503179
Lsr	42.30061587	68.77364657	0.615070132	0.215037574	0.588070988
Atp1b2	2032.147444	3304.900518	0.614889142	7.48E-08	7.94E-06
Ube2s	222.6767268	362.1795568	0.614824119	4.73E-05	0.001107449
Spr	171.1533755	278.4194346	0.614732142	0.003037997	0.027663302
Ifrd2	166.6291999	271.1296049	0.614573978	0.000276953	0.004340243
Vash1	1385.576591	2254.723077	0.614521847	1.29E-07	1.22E-05
Gamt	95.12328343	154.8281352	0.61437983	0.001297852	0.014351064
Atp1a3	3731.09825	6073.075875	0.614367139	3.26E-08	4.30E-06
Csf3r	23.16917031	37.71285109	0.614357431	0.087171369	0.32592897
Ctdsp1	282.9235005	460.5724502	0.614286635	0.08745263	0.326541167
Stub1	448.0477218	729.6486742	0.61405953	2.46E-06	0.000113054
Chpf	1056.999341	1721.510554	0.613995272	1.89E-07	1.60E-05
Nphp4	372.5561393	606.7943816	0.613974273	4.98E-06	0.000195219
Fam110a	427.8826018	696.9750068	0.613913839	0.119591721	0.405522205
Pkm	2117.247278	3448.839775	0.613901316	7.52E-08	7.94E-06
Ehmt2	2390.661663	3894.883673	0.613795395	5.53E-08	6.41E-06
Crabp1	389.2699563	634.3042831	0.613695929	0.000755764	0.00949756
Slc38a3	690.2769605	1124.791447	0.613693287	0.034866691	0.170923496
Kcna3	109.0933286	177.8188531	0.613508223	0.000636797	0.008313795

Svep1	19.69341832	32.10174147	0.613468847	0.704004002	1
Fxyd7	376.6274882	614.0134907	0.613386341	8.41E-05	0.001705648
Bax	457.7575774	746.3286982	0.613345807	2.41E-06	0.000111739
Marcksl1	1502.83912	2450.777619	0.613209092	1.13E-07	1.11E-05
Zfp628	502.3936142	819.8264757	0.612804818	5.36E-06	0.000205737
Mtch1	1707.737872	2787.712042	0.61259479	8.16E-08	8.46E-06
Map3k11	508.7060289	830.4740715	0.61254896	0.008394228	0.059564445
Pold1	660.7329882	1078.718757	0.612516454	0.023668185	0.129212597
C1qb	307.9570599	503.0729792	0.61215186	0.052451979	0.228152341
Rcn3	182.5120081	298.1557282	0.612136514	0.001881158	0.019016697
Tusc1	262.7791405	429.5314538	0.611780903	1.73E-05	0.00050851
Cckbr	15.79655894	25.82133855	0.611763752	0.188408008	0.541229267
Ptms	1528.760086	2499.234164	0.611691417	9.16E-08	9.31E-06
Emc10	1004.426268	1642.100947	0.611671451	2.22E-07	1.81E-05
Atxn2l	2736.469282	4473.849427	0.61165878	3.35E-08	4.36E-06
Ctxn1	985.3093199	1611.987613	0.611238766	2.02E-07	1.68E-05
Rab3a	1226.740461	2007.420613	0.611102852	1.11E-07	1.10E-05
Fam131c	350.404848	573.5003523	0.610993257	4.95E-05	0.001143091
Rpl32	396.1568509	648.5597678	0.61082551	3.77E-06	0.000156925
Ldb1	1758.63923	2879.930812	0.610653292	7.24E-08	7.84E-06
Notch3	359.8378757	589.3050891	0.610613895	0.003425586	0.030247362
Hspb1	92.53101891	151.6182222	0.610289565	0.083406798	0.316439646
Zbtb45	476.6712087	781.3664786	0.610048193	1.30E-06	6.88E-05
Grina	1375.621101	2255.286699	0.609953981	8.47E-08	8.71E-06
Entpd2	50.14321091	82.23258719	0.609772994	0.062918725	0.258739595
Ldha	1316.147452	2159.239489	0.609542137	1.15E-07	1.13E-05
Nod1	14.57347812	23.90932877	0.609531044	0.14709354	0.463057378
Fosb	1943.024763	3188.027292	0.609475574	1.04E-07	1.05E-05
Tuba1b	245.9421815	403.5385939	0.609463841	2.42E-05	0.000656179
Mlxipl	12.12622723	19.91350586	0.608944869	0.238079522	0.625722727
Gga1	1022.411517	1679.084629	0.608910057	1.32E-07	1.24E-05
Xylt1	78.08851811	128.2514033	0.608870672	0.002294104	0.022136269
Rps5	762.8293307	1253.030099	0.608787715	3.58E-07	2.61E-05
Rps26	278.1739276	457.0218961	0.608666521	1.18E-05	0.000378722
Sox4	2052.193741	3375.504523	0.607966521	4.17E-08	5.19E-06
Rorc	27.25470264	44.83121665	0.607940285	0.052731412	0.228842244
Gpr62	110.0153637	180.9741013	0.607906672	0.000583715	0.007781695
Ncln	985.2509961	1620.794754	0.607881407	1.47E-07	1.36E-05
Tubb4b	588.862547	969.2182562	0.60756444	5.97E-05	0.00130946
Gpr133	21.57023229	35.50610916	0.607507632	0.465447589	0.884083687
Ppp1r35	386.4655145	636.2250488	0.607435239	4.11E-05	0.000995532
Nkain1	759.0670305	1249.766002	0.607367323	2.63E-07	2.05E-05
Mxd3	195.3141755	321.6529321	0.607220255	0.091508081	0.337071743

Mrps34	91.01877078	149.9175142	0.607125667	0.001215964	0.013737754
Rhob	1522.328992	2507.468052	0.607118001	6.78E-08	7.46E-06
Map3k10	1702.539873	2804.384384	0.607099327	3.75E-08	4.77E-06
Mafa	20.7317869	34.14985561	0.60708271	0.074112859	0.290964847
Calm3	2254.250739	3713.359124	0.607065103	3.05E-08	4.13E-06
Abhd8	1207.465637	1989.117579	0.607035828	7.85E-08	8.19E-06
Gnb3	569.6417957	938.4823992	0.606981864	0.130531065	0.431404927
Tysnd1	492.6616987	811.7042471	0.606947297	1.26E-06	6.67E-05
Ppp1r14b	419.7560333	691.7368495	0.60681462	4.56E-06	0.000183595
Unc119	856.256009	1411.085827	0.606806469	2.14E-07	1.75E-05
Snord104	48.34089439	79.68951117	0.606615522	0.024661915	0.132997941
Reep4	155.1003929	255.696632	0.606579726	0.006903091	0.051445298
Mapk8ip1	2223.331991	3665.654316	0.606530731	2.55E-08	3.60E-06
Ccdc85b	285.3596893	470.5442854	0.606445978	9.95E-06	0.000329101
Prrt1	289.0377511	476.6391872	0.606407863	0.000850672	0.010409055
Panx2	688.2380375	1135.214582	0.606262506	0.002493432	0.023663511
Rpl28	465.809229	768.4453589	0.606170919	1.24E-06	6.59E-05
Kcnk9	41.73790905	68.85776897	0.606146694	0.194219582	0.551797538
Mbd6	1576.861694	2602.212346	0.605969646	3.97E-08	5.00E-06
Srebf1	1116.702955	1843.240794	0.605836719	0.015540388	0.094251814
Rcor2	1228.672395	2028.576383	0.605682096	0.001308178	0.014441157
Thbs4	35.35652792	58.41019092	0.60531437	0.524645929	0.940765474
Pkmyt1	342.5951225	566.0227328	0.605267426	2.02E-05	0.000579474
Dusp5	902.7746843	1491.568492	0.605251914	0.032535811	0.162440135
Gstp1	193.1054777	319.068621	0.605216135	3.96E-05	0.000968994
Lhx2	1174.030105	1940.171253	0.60511674	0.034402124	0.169460657
Epn1	1762.658937	2913.787715	0.604937322	3.23E-08	4.28E-06
Pnrc1	720.5799161	1191.417083	0.604809119	0.006262074	0.04768672
Dtx1	2436.773517	4029.318266	0.604760745	1.65E-08	2.52E-06
Nsg1	1569.882871	2597.509552	0.604380019	3.96E-08	5.00E-06
Pcolce	66.61422128	110.2403464	0.604263534	0.385222346	0.813455102
Sac3d1	324.3980721	536.8812054	0.604226911	5.14E-06	0.00020008
Clec2l	783.9653615	1297.56239	0.604183173	1.49E-07	1.36E-05
Med25	991.4702683	1641.083711	0.604155816	1.07E-07	1.07E-05
Ctbp1	1866.632913	3090.287242	0.604032171	3.04E-08	4.13E-06
Ptprs	5480.194976	9073.645751	0.603968364	1.04E-08	1.82E-06
Lamc3	86.56972074	143.3510635	0.603900094	0.159449747	0.488358173
Tead3	193.5333113	320.4970219	0.603853696	0.002356406	0.02261399
Phyhip	32.93810429	54.54870705	0.603829239	0.052550042	0.228354132
B3gat3	799.2051852	1323.798904	0.60372099	1.68E-07	1.49E-05
Pcbp2	840.9411451	1393.202303	0.60360304	1.60E-07	1.44E-05
Sox12	1954.93921	3239.463301	0.603476264	2.29E-08	3.37E-06
Bak1	351.5196189	582.8442412	0.603110735	2.51E-06	0.000114107

Zfp579	724.2545637	1200.970355	0.60305782	1.70E-07	1.49E-05
Zfp865	707.4239726	1173.102329	0.603036884	2.10E-07	1.73E-05
Emilin3	24.47340998	40.60113005	0.602776572	0.325279768	0.745601514
Gpx4	401.9593515	666.8511665	0.60277221	0.00020519	0.003437021
Rps29	166.230487	276.0022244	0.602279519	7.31E-05	0.001523148
Pld3	984.1192763	1634.43479	0.602115962	6.56E-08	7.28E-06
Fhl3	21.51159447	35.73126415	0.602038438	0.076082831	0.296487385
Tcf7l1	123.9280273	205.8830704	0.601934035	0.02316576	0.127229346
Atp5l	15.22999735	25.30358381	0.60189092	0.122671178	0.412914122
Fth1	789.7450476	1314.185672	0.600938714	1.56E-07	1.41E-05
Apoe	857.5160223	1427.173025	0.600849377	1.81E-05	0.000529632
Samd14	2564.634018	4271.888973	0.600351281	1.08E-08	1.85E-06
Sfrp2	301.8035076	502.808284	0.60023575	0.063141468	0.259373999
Thy1	1424.389429	2373.557286	0.600107458	2.48E-08	3.52E-06
Slc2a1	1407.122082	2345.966875	0.599804753	0.018059986	0.105317483
Cd34	126.6467625	211.1671085	0.599746634	0.45567708	0.876872611
Atoh8	74.32713951	123.9407107	0.599699155	0.27563836	0.681933618
Dmwd	1297.90733	2164.740903	0.599567056	3.18E-08	4.26E-06
Fgfr4	40.5861196	67.71129168	0.599399577	0.01659632	0.098936155
Wtip	309.9029668	517.0867449	0.59932491	4.02E-06	0.000165067
Zfp385a	1503.61679	2509.724742	0.599116216	2.39E-08	3.43E-06
Rhog	86.85348291	144.9783674	0.599078914	0.001853321	0.018806985
Pdgfb	147.2187891	245.762601	0.599028446	0.008735065	0.061404818
Scara5	37.10158939	61.98986405	0.598510578	0.600357146	0.988955325
Stab1	377.702325	631.1233443	0.598460394	0.466054625	0.884368812
Emp1	70.94697899	118.5816148	0.598296617	0.649057091	1
Mfap4	247.8003704	414.2006785	0.598261624	0.000526211	0.00717748
Dlk2	454.7120899	760.1732727	0.598169005	4.32E-07	2.99E-05
Gnai2	1596.334429	2669.793139	0.597924388	2.49E-08	3.52E-06
Ftl1	420.6040321	703.6982641	0.597705087	8.99E-07	5.29E-05
Gtf3a	141.0383793	236.0816288	0.597413615	0.000110135	0.00210703
Rasl10b	1631.580889	2732.341132	0.597136598	1.83E-08	2.76E-06
Kcnq4	297.1491093	497.7068387	0.59703642	0.02416841	0.131079886
Ascl1	1016.818827	1703.745791	0.596813699	0.058394734	0.246011243
Nid2	85.69182599	143.5905229	0.596779121	0.452613528	0.876560011
Rpl38	135.7603108	227.9058795	0.595685864	0.000124981	0.002331073
Cnih2	955.7363534	1604.488599	0.59566416	4.21E-08	5.22E-06
Cherp	1184.724027	1989.30558	0.595546526	2.60E-08	3.63E-06
Ywhah	925.063656	1553.587377	0.59543716	4.67E-08	5.65E-06
Ttyh3	3017.602512	5068.66597	0.595344521	5.99E-09	1.24E-06
Il17rc	24.72103481	41.53679989	0.595159831	0.0559183	0.238364928
Fam195a	52.63334593	88.45711139	0.595015427	0.005379529	0.042428196
Selplg	63.81776194	107.270021	0.594926349	0.005993193	0.046129319

Fam132a	271.1478439	455.8824373	0.594775806	0.009849705	0.066957748
Lrp5	938.9111668	1578.616041	0.594768546	0.002076276	0.02056576
Mesdc1	938.1461502	1577.356634	0.594758427	4.58E-08	5.60E-06
Scrt1	2782.973774	4679.618128	0.594701042	4.43E-09	9.86E-07
Rasal3	32.24115238	54.22238493	0.594609633	0.155719716	0.481081051
Stmn3	965.7262634	1624.725038	0.594393661	4.01E-08	5.01E-06
Emilin2	158.1093231	266.0381559	0.594310702	0.105163683	0.371012694
Igsf8	1039.8795	1749.884953	0.594255925	3.21E-08	4.27E-06
Foxp4	875.2635381	1473.927579	0.593830763	3.76E-08	4.77E-06
Rtn4r	354.2268753	596.5318396	0.593810509	0.001429191	0.015471265
Chst12	167.7370365	282.4994085	0.593760664	0.002246931	0.021880142
Crip2	887.1747165	1494.281947	0.593713066	4.90E-08	5.75E-06
Mdga1	326.2198301	549.5565663	0.593605554	1.67E-06	8.26E-05
Foxj1	244.1602281	411.342465	0.593569225	0.000142491	0.002594983
Ankle1	81.89860537	138.0392758	0.593299298	0.273057563	0.678851966
Hmg20b	335.7295133	566.4285171	0.592712943	0.042014027	0.195343491
Zfp219	1260.179912	2126.192673	0.592693187	0.003337973	0.029632276
Cldn5	151.7707233	256.0703742	0.592691457	0.317823293	0.736600635
Pcbp4	1814.733663	3062.523786	0.592561492	1.04E-08	1.82E-06
C1s	11.20942962	18.9179745	0.592528001	0.639282693	1
Rasl10a	21.16242714	35.71804642	0.592485571	0.057148434	0.242004354
Cxx1a	114.9256192	194.1955027	0.591803711	0.000215563	0.00355244
Slc22a17	1674.72458	2830.111051	0.591752249	7.54E-09	1.47E-06
Rgma	589.7177095	996.6262854	0.591713984	0.000756673	0.009499974
Hes6	1082.224216	1829.218655	0.591631959	0.000673142	0.008673002
Atp5g2	63.29517372	106.9857672	0.591622375	0.002680036	0.025102028
Znhit2	219.3688441	370.8532625	0.591524644	1.23E-05	0.00039141
Fkbp10	86.28059455	145.8961448	0.59138365	0.113980464	0.391928223
Cdk2ap1	175.3513865	296.5347937	0.591334947	0.000513646	0.00704231
Ngfr	920.5608705	1557.060008	0.591217336	4.43E-08	5.44E-06
Zfp575	268.551117	454.2445142	0.591203875	2.75E-06	0.000122008
Gas2l1	367.5571824	621.7686135	0.591147855	7.72E-07	4.74E-05
Ndufs7	438.0964416	741.1160724	0.591130672	4.66E-07	3.19E-05
Slc30a3	41.91704914	70.91212455	0.591112583	0.018618472	0.107650985
Tagln2	570.2831451	965.2537405	0.59081164	0.005916158	0.045664739
Abcc9	48.11151582	81.44930369	0.590692782	0.461753156	0.880915655
Adra1b	42.12951994	71.36720766	0.590320419	0.057990024	0.244695016
Spint1	56.99660715	96.55561529	0.590298213	0.134228184	0.43924405
Pgp	455.7704427	772.5254314	0.589974678	3.63E-07	2.63E-05
Enho	583.8331926	989.7266493	0.589893374	1.20E-07	1.15E-05
Nrxn2	4295.097119	7281.849623	0.589836009	1.04E-08	1.82E-06
Nlgn2	3279.596293	5561.406153	0.589706309	2.27E-09	5.90E-07
Fam57b	1192.918828	2023.356105	0.589574334	1.07E-08	1.85E-06

Lrrc32	33.90228948	57.50450151	0.58955888	0.378880715	0.805088727
Adra2c	99.03493376	168.0870716	0.589188287	0.000313083	0.004776593
Midn	2610.798729	4431.760938	0.589110912	3.73E-09	8.83E-07
Il3ra	97.86384991	166.2158027	0.588775846	0.000358817	0.005318267
Tmem151b	1319.244875	2242.77803	0.5882191	6.32E-08	7.10E-06
Rbm15b	1391.252115	2365.349551	0.588180345	1.02E-08	1.81E-06
Olfml2a	54.86663732	93.28476743	0.588162878	0.475147996	0.893104435
Sstr4	24.20154673	41.15094116	0.588116482	0.091552338	0.337096561
Rhbdf1	140.8986032	239.631378	0.587980607	0.00106806	0.012377919
Ppard	256.0247942	435.4612737	0.587939295	0.000604567	0.007995358
Eef1a2	1319.950332	2246.492381	0.587560565	6.24E-09	1.26E-06
St14	259.2725093	441.2904348	0.587532584	3.45E-05	0.000870138
Klf4	301.1577775	512.6506941	0.587452199	0.052188453	0.227387236
Npy	77.26337496	131.6421433	0.586919758	0.001264318	0.014074817
Rbp3	2527.687012	4308.340084	0.58669626	4.83E-08	5.71E-06
Foxo6	379.4547446	646.7800354	0.586682835	5.81E-07	3.81E-05
Mdk	409.3680591	697.859076	0.586605625	0.042592653	0.196928823
Efcab6	12.54142785	21.38184276	0.586545696	0.152499742	0.474666411
Itpripl2	89.20970747	152.2148455	0.586077575	0.294624947	0.706353987
Col2a1	2005.254318	3423.072465	0.585805395	0.037413048	0.179653093
Zfp580	478.857512	817.5956992	0.585689862	1.43E-07	1.33E-05
Tcf3	1277.72239	2181.863201	0.585610679	0.003229564	0.028960133
Guca1a	23.49657123	40.12588149	0.585571466	0.046385985	0.209392367
Junb	1033.026712	1764.182151	0.585555585	1.68E-08	2.54E-06
Rnf208	675.1949716	1153.099292	0.585547989	1.22E-07	1.18E-05
Lrrc25	14.27276706	24.37662702	0.585510336	0.279342496	0.687684117
Steap3	50.69326418	86.64563322	0.58506427	0.038114997	0.182067602
Notch1	3354.29057	5733.390094	0.585044889	0.000263054	0.004154347
Rabac1	613.8682868	1049.327236	0.585011297	6.83E-08	7.49E-06
Tssk6	59.70491161	102.121098	0.584648156	0.00268382	0.02512191
Zdhhc22	135.6419664	232.0206912	0.584611509	0.000209088	0.003482589
Sparc	881.1313541	1508.135809	0.584251994	0.022794628	0.125815804
Kif19a	542.0651589	927.8544317	0.58421358	0.009867897	0.067022839
Rplp1	351.6089485	602.0249623	0.584043803	6.12E-07	3.97E-05
Adamtsl4	45.26322712	77.5004209	0.584038468	0.363544866	0.788463574
Plxnb3	11.79465747	20.19670058	0.583989322	0.355384188	0.779499307
Rom1	472.7419608	809.5750748	0.583938384	2.76E-07	2.12E-05
Atp5d	672.8580414	1153.368322	0.583385228	3.69E-08	4.72E-06
Il21r	12.58741211	21.60800034	0.582534798	0.179962382	0.527687993
Dbp	135.5583561	232.7862237	0.582329804	5.96E-05	0.00130946
Dexi	317.9932072	546.0712255	0.582329177	9.27E-07	5.39E-05
Egr1	2008.539947	3453.250757	0.581637445	6.51E-08	7.25E-06
Ost4	127.3412867	218.9761065	0.581530509	9.10E-05	0.001814416

Slc17a7	176.7971402	304.0925387	0.581392562	1.84E-05	0.000535635
Tead2	735.2249174	1265.2877	0.581073314	0.072866082	0.287132861
Slit3	28.31131748	48.74523297	0.580801768	0.296976794	0.708851982
Ednra	27.01510141	46.53955009	0.580476205	0.548841537	0.961551515
Wnt7b	97.64628804	168.2360545	0.580412375	0.05394683	0.232631998
Aes	1207.700629	2081.728506	0.580143196	5.07E-09	1.10E-06
Mmp17	287.4042255	495.4594259	0.580076209	9.59E-07	5.49E-05
Kcne1l	221.3706433	381.7482976	0.579886393	0.011848635	0.076572852
Siglec1	18.46755143	31.84844214	0.579857292	0.602368277	0.988955325
Lama2	110.1196274	189.919816	0.579821683	0.587583107	0.988955325
Fbn1	619.0303677	1068.041458	0.57959395	0.537739935	0.952157162
Scrt2	1594.582433	2752.561444	0.57930857	2.18E-09	5.73E-07
C1qc	316.4449034	546.5450329	0.578991454	0.029134234	0.150385092
Cdc42ep5	51.59464272	89.14254616	0.578788075	0.005079225	0.040750603
Rpl23a	33.33817934	57.62391393	0.578547639	0.017792061	0.104052558
Espn	933.2347849	1613.370513	0.578437983	0.045110517	0.205418248
Parp4	32.7955421	56.7227292	0.578172852	0.017368447	0.102229022
Hcn2	561.6066498	971.5500598	0.5780522	2.83E-08	3.89E-06
Cox6a1	455.7202896	788.3884638	0.578040282	1.06E-07	1.07E-05
Naprt1	22.22011068	38.44179464	0.578019598	0.082137552	0.313195186
Susd3	14.5913692	25.24547334	0.577979624	0.108318496	0.379143875
Rplp2	340.7542583	589.8902474	0.577657047	4.11E-07	2.88E-05
Ttyh2	564.9728557	978.6270762	0.577311695	5.56E-06	0.000210436
Chadl	247.744184	429.1910947	0.577235145	1.31E-06	6.89E-05
Chchd10	111.9016251	193.9762928	0.576882997	0.000127799	0.002373047
Pgam1	187.960565	325.8654797	0.576804162	7.36E-06	0.000261429
Tceb2	285.4945214	495.1968488	0.576527339	9.20E-07	5.37E-05
Crlf2	334.260525	579.8267634	0.576483436	2.78E-07	2.13E-05
Ckb	1682.474159	2919.179274	0.576351776	1.85E-09	4.90E-07
Igfbp2	502.0918787	871.7127079	0.575983204	0.001380225	0.015085758
Bai2	1967.97176	3417.258331	0.575892007	1.03E-09	3.03E-07
Erbb3	18.04847548	31.37839025	0.575188062	0.3574301	0.782059058
Sh3bgrl3	282.9113492	492.3526998	0.574611146	7.27E-07	4.58E-05
Cpne6	53.72157409	93.61124535	0.573879494	0.016210453	0.097010877
Cryab	14.72871442	25.67270843	0.573710969	0.593210528	0.988955325
Zfp771	382.7692476	667.1828132	0.573709694	1.40E-07	1.31E-05
E2f1	690.093798	1202.925236	0.573679708	0.068217654	0.273486814
Fscn1	2386.130112	4164.151074	0.573017182	5.91E-10	1.89E-07
Dll3	511.9001972	894.0345132	0.572573194	0.079445379	0.305527924
Ssbp4	894.8273001	1563.34229	0.572380921	4.93E-09	1.08E-06
Cks2	75.26926479	131.7104073	0.571475454	0.10025434	0.359284238
Tmem204	10.26175274	17.95661764	0.571474704	0.157959723	0.48550597
Cxcl14	267.3581197	467.9614847	0.571325052	0.01095265	0.072442124

Eif4ebp1	85.13726926	149.0236442	0.571300411	0.027196094	0.142854919
Tmem132a	1337.386241	2341.409804	0.571188452	1.44E-09	3.97E-07
Cited4	23.92335671	41.88406448	0.5711804	0.035880377	0.174644579
Mpv17l2	199.5626331	349.5191083	0.570963442	0.001395786	0.015199503
Rps4y2	405.6261425	710.6032662	0.570819418	7.62E-08	8.02E-06
Nbl1	115.5132131	202.5032742	0.570426397	0.200532482	0.563872878
Ube2ql1	1757.570798	3081.224472	0.570413098	4.92E-10	1.61E-07
Htra1	190.7023877	334.4726439	0.570158401	0.000258256	0.004095183
Tgfbi	69.00763131	121.0827075	0.569921442	0.536624874	0.951684154
Gnb2	1491.760121	2618.497021	0.569700905	9.68E-10	2.88E-07
Tpsb2	39.76623454	69.81099114	0.56962713	0.685786109	1
Nrl	530.6994854	931.7653273	0.569563462	0.001180514	0.013377144
Vps25	17.60241616	30.91912736	0.569305076	0.059894659	0.250381016
Tmco4	12.95941454	22.77188944	0.569097025	0.126244972	0.420805994
Nupr1l	12.00819697	21.10209367	0.569052396	0.135988368	0.443090142
Gse1	1270.966996	2233.812585	0.568967605	1.28E-09	3.65E-07
Cdk5r2	1378.741379	2423.260191	0.568961346	6.54E-10	2.04E-07
Vtn	23.49303053	41.29565923	0.568898305	0.072810242	0.287021081
Mif	283.8355628	498.9487774	0.568867138	4.69E-07	3.20E-05
Slc1a7	141.8564616	249.5296113	0.568495502	0.021523848	0.120559009
Irf2bpl	1876.909309	3303.634432	0.568134686	4.28E-10	1.45E-07
Hes5	290.8315418	512.0714404	0.567951108	0.001169738	0.01327773
Lox	79.18193955	139.4359164	0.56787334	0.625635051	1
Arsi	17.76150723	31.29971867	0.567465395	0.646453031	1
Ddr1	2543.122884	4482.547477	0.56733875	1.54E-08	2.38E-06
Csf2ra	402.2233382	709.0790649	0.567247516	4.38E-08	5.40E-06
Unc5a	578.6513032	1020.291847	0.567142926	8.19E-09	1.53E-06
Mcam	564.7688279	995.8350219	0.567130916	0.044426008	0.203313776
Erbb2	139.0726764	245.4648039	0.566568706	0.054322987	0.233835766
Prrt4	314.5055769	555.1129831	0.566561378	4.72E-06	0.000188093
Rnf187	1837.686569	3244.015416	0.566485153	4.06E-10	1.45E-07
Epas1	251.9630597	445.2457567	0.5658966	8.73E-05	0.001749203
C1qtnf1	27.68534288	48.93209014	0.565791136	0.178835311	0.526070237
Abhd17a	802.8821989	1419.187113	0.565733857	3.53E-09	8.58E-07
Apba3	244.8448611	432.8982578	0.565594471	8.76E-07	5.18E-05
Arhgap9	15.66449236	27.70965221	0.565308155	0.08339085	0.316426988
Tmem119	78.65463914	139.1881111	0.565095959	0.055470615	0.237235296
Sf3b5	300.2829218	531.5645174	0.564903999	4.53E-07	3.12E-05
Mfng	346.9762748	614.9323553	0.564251128	0.06623819	0.268558386
Igsf21	666.5935807	1181.428345	0.564226839	5.88E-09	1.23E-06
Atf5	386.8406171	687.2396103	0.562890455	0.000256895	0.004080017
Gja4	50.8913846	90.57814898	0.56185057	0.056382212	0.239818626
Kcng2	16.19032777	28.8269118	0.561639342	0.122187741	0.411932073

Ubald2	163.9089965	292.0615047	0.56121397	5.96E-06	0.000222047
Unc93b1	171.1918378	305.3366238	0.560665916	0.054834673	0.235160309
Sardh	65.77466607	117.3925616	0.56029671	0.02049435	0.115943716
Rcsd1	29.69594081	53.00902765	0.560205349	0.071567313	0.283277934
Nfkb2	50.87209021	90.81828704	0.560152496	0.005540027	0.043448496
Dos	1955.302015	3491.617442	0.559998925	1.11E-10	5.36E-08
Msi1	2411.934684	4307.244304	0.559971646	1.50E-06	7.63E-05
Lgals1	125.0229168	223.3753976	0.559698687	0.082868749	0.31516749
Dpep1	16.83697952	30.08781612	0.559594603	0.635040075	1
Rgl3	163.8538994	292.8611974	0.559493374	0.120524415	0.407812568
Blvrb	47.02925925	84.07013374	0.559405072	0.00315011	0.02844001
Spata5l1	206.8127518	369.7384038	0.559348852	1.04E-06	5.77E-05
Lingo3	356.59898	637.9785999	0.558951319	4.63E-08	5.63E-06
Scn1b	224.2047287	401.2928486	0.558706016	0.003313491	0.029499643
Trnp1	619.6626168	1109.166054	0.558674343	3.06E-09	7.66E-07
Zfp541	10.23011886	18.31328726	0.558617288	0.140962931	0.451312858
Pim1	296.701479	531.209726	0.558539244	0.029637945	0.152243266
Pcsk6	93.33644766	167.245974	0.558078891	0.08597205	0.322749696
H2-Ab1	25.4348091	45.6029627	0.557744664	0.023856665	0.129893908
Romo1	239.7761427	430.0467655	0.557558298	3.95E-07	2.81E-05
Tbx2	1675.830741	3007.427414	0.557230653	1.85E-10	7.44E-08
Tmem259	1179.05416	2117.542099	0.556803173	3.66E-10	1.33E-07
Klc3	190.751264	342.6502858	0.556693725	0.028447797	0.147819281
Epha2	686.7474935	1233.700635	0.556656513	0.12243662	0.412310061
Vegfb	547.752477	984.1312291	0.556584794	4.39E-09	9.86E-07
Btbd17	528.0414194	948.9914762	0.556423775	0.107517442	0.376908049
Cox8a	325.6612716	585.4879904	0.55622195	6.74E-08	7.45E-06
Pcbp1	1274.644996	2293.003772	0.555884387	2.80E-10	1.06E-07
Dlgap3	2242.066209	4033.861188	0.555811443	5.59E-11	2.80E-08
Il17d	231.3092904	416.3410354	0.555576488	3.97E-07	2.82E-05
Ass1	162.7616694	293.1815744	0.555156543	0.000587706	0.007823095
Neurog1	22.33736576	40.29186695	0.554388949	0.060880509	0.253007728
Creb3l1	43.23813176	78.04533166	0.554013044	0.291480661	0.702381469
Fcgbp	19.38682105	34.99675686	0.553960503	0.03911787	0.185787109
Islr	120.4487962	217.6204875	0.55348096	0.45636438	0.876872611
Evl	1422.576576	2572.514003	0.5529908	1.23E-10	5.54E-08
Hrh3	23.42421117	42.3917365	0.552565502	0.023657352	0.129180056
Slc8b1	21.54599388	38.99746582	0.552497282	0.032051634	0.160608269
C1qa	259.6522617	470.2758894	0.552127523	0.008856772	0.061937722
Fam171a2	2007.62979	3636.512831	0.552075542	4.80E-11	2.45E-08
Ephb4	157.3814713	285.2172816	0.551795005	0.00450512	0.037166157
Pdgfrb	134.286085	243.3929515	0.551725447	0.023609391	0.129024448
Slc6a9	216.3464005	392.4083919	0.551329699	3.73E-07	2.69E-05

Prr19	31.62860653	57.42070401	0.55082234	0.007947531	0.057327472
Cxx1c	474.3546211	861.721569	0.55047319	5.48E-09	1.18E-06
Jund	889.535844	1616.83326	0.550171663	4.54E-10	1.50E-07
Mfap2	333.0377171	605.4095452	0.550103182	0.002805997	0.02594586
Tspan11	45.63830913	82.99844458	0.549869451	0.002678919	0.025100427
Rhpn1	161.7329807	294.2025673	0.549733411	0.052514469	0.228346368
Mmp28	40.97215835	74.53981114	0.549668127	0.003758906	0.032450544
Arid3c	43.37929063	78.94244954	0.549505252	0.068219029	0.273486814
Pde6g	95.02840234	172.9702117	0.549391721	0.001833192	0.01864554
Zfp36l2	929.3730353	1693.23571	0.548873987	0.004287493	0.035791954
Mfap5	93.96093356	171.1929357	0.548859877	0.500183368	0.918002252
Alx4	16.00733292	29.17202969	0.548721946	0.494132649	0.912170796
Uba52	133.8919815	244.0138652	0.548706449	7.49E-06	0.000264466
Boc	96.84722718	176.5338427	0.548604311	0.000738993	0.009326583
Tmem158	490.7573991	895.2496784	0.548179364	3.85E-09	9.05E-07
Yif1b	291.4869718	532.0982863	0.547806635	5.93E-08	6.73E-06
Spi1	76.90819492	140.5938471	0.547023903	0.00160372	0.016935184
Prph2	413.2772785	755.8868259	0.546744915	9.33E-09	1.69E-06
Rgcc	33.71820537	61.67615273	0.546697611	0.172519957	0.513576341
Slc12a4	121.4452877	222.186937	0.546590584	0.006419921	0.048581802
Grin2d	639.9771074	1172.523287	0.545811853	0.037191931	0.17911029
Lrrc17	29.80026939	54.61956884	0.545596936	0.665455206	1
C2cd4b	83.91001961	153.8114072	0.545538339	0.000109669	0.002099618
Fam89a	68.04248336	125.0715129	0.544028626	0.000864029	0.010523927
Mrps36	38.53123423	70.8282563	0.544009358	0.00391958	0.033558792
Maz	2017.571852	3724.377582	0.541720545	1.53E-11	9.43E-09
Bcl2l12	86.49488867	159.7579191	0.541412214	0.041258468	0.19318317
Rax	516.0518039	954.2362288	0.540800892	4.60E-06	0.000184615
Slc32a1	290.5081427	537.5210555	0.540459094	3.35E-08	4.36E-06
Rab4b	26.56238069	49.15656744	0.540362805	0.011491086	0.074809142
Sema5b	573.8986526	1063.357805	0.539704181	0.012000057	0.07738169
Map1lc3a	737.9049174	1368.39131	0.53924993	1.46E-10	6.34E-08
Lrrc4b	1611.387674	2988.207379	0.539248944	1.15E-11	7.76E-09
Hhipl1	85.11799542	157.8590991	0.539202339	0.001001171	0.011798685
Cyp2d22	48.30402298	89.60049584	0.539104416	0.038325407	0.1829738
Elfn1	506.0307828	940.0207934	0.538318712	1.51E-06	7.68E-05
Gpr150	43.52725795	80.92886607	0.537845889	0.001649817	0.017304801
Rapgef3	21.46592428	39.92717626	0.537626907	0.042380118	0.196383992
Nfix	634.4579309	1180.718084	0.537349211	1.89E-07	1.60E-05
Rcvrn	39.91585757	74.37488474	0.536684631	0.062955303	0.258751581
Kcns1	27.48336769	51.24458893	0.536317458	0.055403134	0.236984917
Arf5	717.9557036	1338.682432	0.536315175	1.51E-10	6.45E-08
Fxyd2	28.19326665	52.5915082	0.536080208	0.008682494	0.06114874

Irf2bp2	1442.191854	2693.055995	0.535522417	1.17E-11	7.76E-09
Trem2	97.57841087	182.23783	0.535445417	0.018591814	0.10760626
Aebp1	58.17621684	108.6641203	0.535376504	0.335277126	0.75597009
Dact3	1396.846649	2610.249759	0.535139078	1.06E-11	7.40E-09
Spon2	38.96434649	72.85257499	0.534838288	0.024116135	0.130876676
Hs6st3	40.61528148	75.97613453	0.534579467	0.068368863	0.273847246
Flt4	12.09568259	22.64558576	0.534129818	0.086753895	0.324772876
Slc5a5	48.93632435	91.83151294	0.532892498	0.018972561	0.109340673
Prima1	18.43945824	34.63939525	0.532326217	0.033377153	0.165549679
Fbln5	37.83710952	71.09707488	0.532189399	0.540081682	0.95368088
Sh3bp1	70.83596845	133.1066532	0.532174514	0.001225869	0.013820208
Zbtb7b	180.0852674	338.6463854	0.531779683	5.12E-07	3.44E-05
Nr2e3	396.644558	746.243516	0.531521614	0.00997638	0.067603803
Anxa11	18.929805	35.63523723	0.531210298	0.031176095	0.1575258
Scn4a	17.33826245	32.65505467	0.530951873	0.038365322	0.183032545
Fgf22	11.63340846	21.92568017	0.530583698	0.074469129	0.291758779
Col14a1	177.3333416	334.3331063	0.530409159	0.587617265	0.988955325
C1qtnf6	39.54249294	74.59646689	0.530085332	0.187670228	0.540076379
Esrrb	71.20767738	134.5955091	0.52904943	0.014993737	0.09167026
Tnfaip8l2	28.20977498	53.32395384	0.529026318	0.007839027	0.056807774
Gas1	1025.246274	1938.152695	0.528981167	0.016464992	0.098307979
Ccdc80	27.04426328	51.1394533	0.528833641	0.40161101	0.834308802
Col15a1	128.1287184	242.294176	0.528814685	0.443594826	0.876560011
Rpl29	242.3351176	458.4321225	0.528617227	2.89E-08	3.95E-06
Bmp3	24.40490468	46.1874702	0.528387993	0.096874042	0.351204824
Tns1	133.1448117	252.0267488	0.528296351	0.341029093	0.761428716
C1qtnf4	860.4936569	1628.916756	0.528261284	1.96E-11	1.10E-08
Ptger3	10.62881115	20.15495714	0.527354689	0.089227442	0.331041675
Fcrlb	11.78585869	22.36966367	0.526867943	0.079769596	0.306368861
Cebpa	120.1259225	228.0481967	0.52675673	0.061444353	0.254730842
Epha8	1834.917534	3486.48545	0.526294333	2.92E-09	7.45E-07
Gng10	30.39672692	57.77247329	0.526145501	0.004994192	0.040153425
Rnf126	667.8499717	1270.064689	0.525839335	4.78E-11	2.45E-08
Snord55	37.04647171	70.47335201	0.525680568	0.002254588	0.021914465
Cox16	49.79620153	94.7323884	0.525651283	0.000614968	0.008103664
Gpr116	27.05691684	51.51522931	0.52522171	0.423434105	0.85964387
Gjd3	20.76480354	39.55384328	0.524975624	0.016276743	0.097315473
H2afx	659.3177143	1256.899477	0.524558826	0.000299262	0.004613509
Rd3	146.8850408	281.0401939	0.522647806	0.000797588	0.009896507
Emilin1	110.3258363	211.1398262	0.522524993	0.000661659	0.008558322
Slc16a3	315.631871	604.0595052	0.522517845	0.000112279	0.002140317
Cox5a	243.953036	467.4614388	0.521867721	1.23E-08	1.99E-06
Hmga1	470.8719347	902.961321	0.521475199	2.21E-10	8.63E-08

Erf	665.3402488	1276.032863	0.521413098	1.93E-06	9.28E-05
Itih5	115.5788675	221.8823467	0.520901591	0.582894635	0.988300667
Lrrc24	73.6559764	141.4198909	0.5208318	4.23E-05	0.001017404
Sncb	742.2257762	1427.267331	0.520032765	1.67E-10	6.93E-08
Bgn	143.2213468	276.2528246	0.518443013	0.573364816	0.979123177
Ly6h	1078.447753	2080.174889	0.518440906	1.85E-12	1.88E-09
Cyba	78.72283037	151.9237712	0.518173224	0.015069707	0.09204984
Copz2	18.48405975	35.67590732	0.518110432	0.345561671	0.767863342
Wnt9a	23.76598303	45.89831608	0.517796404	0.193833751	0.551055616
Zfp36l1	561.0238323	1084.446553	0.517336544	4.33E-05	0.001032368
Mrc2	91.93097955	177.7115565	0.517304453	0.095195713	0.346827362
Six5	107.9586139	208.7763769	0.517101673	0.183984132	0.533944839
Baiap2l2	51.1794421	99.38894736	0.514940982	0.000287567	0.004469592
Tmem238	77.03190327	149.9539902	0.513703591	0.00089202	0.010769865
Ltb4r2	17.91362283	34.90142192	0.51326341	0.018608474	0.107616639
Ppp1r1b	16.13939949	31.44628713	0.513237045	0.031697069	0.159393036
Rin3	45.63336511	88.93272702	0.513122296	0.005721038	0.044517224
Plxnd1	666.7227758	1301.092382	0.512433079	2.03E-08	3.01E-06
Pde6b	141.4949342	276.3929978	0.51193386	2.94E-07	2.24E-05
Llgl2	119.9434093	235.1853158	0.509995315	3.54E-06	0.000149094
Rtn4rl2	346.3751879	679.4485627	0.509788683	5.17E-06	0.000200813
Tfeb	29.3643505	57.83439647	0.507731597	0.003814084	0.0328358
Pcsk1n	721.8249104	1422.596636	0.507399562	2.02E-12	1.99E-09
Caskin2	396.5723862	781.6599955	0.507346402	0.026204449	0.138995235
Acta2	20.5340011	40.56420191	0.506209913	0.559533502	0.970917502
Ankrd33	33.71018177	66.70819138	0.505337966	0.001933722	0.019436909
Hmx1	838.0897762	1660.206307	0.504810621	1.66E-09	4.48E-07
Ndufa4l2	256.2838157	507.7154879	0.504778408	0.011427167	0.074593686
Mtag2	26.76821065	53.07654316	0.504332216	0.004206978	0.035318447
Fxyd1	60.1204263	119.4295053	0.503396762	0.022330119	0.123897349
Lactbl1	51.86897798	103.1357358	0.502919552	0.021637454	0.120939865
Col12a1	87.96586412	175.5404285	0.501114557	0.585995521	0.988955325
Mapk15	11.64467926	23.25730576	0.50068909	0.373970341	0.798299904
Mex3d	1180.156885	2357.62551	0.50057012	1.20E-08	1.97E-06
Col3a1	1169.235003	2335.89851	0.50055043	0.478660447	0.895453676
Gnas	687.4167491	1375.609373	0.499717989	7.50E-13	9.47E-10
Irx1	229.0805436	458.5824336	0.499540599	8.15E-05	0.001664062
Igf2	1010.233594	2028.701169	0.497970627	0.274151711	0.680210234
Derl3	20.60219233	41.47870371	0.496693254	0.008418683	0.059687112
Prelp	166.7467484	335.7524291	0.496636015	8.80E-05	0.001760722
Smad6	77.58721462	156.3981192	0.496087901	0.135396192	0.441812136
Ism1	23.81303598	48.01668508	0.495932527	0.005746158	0.044644705
Osr2	19.8290256	40.02457285	0.495421292	0.272891996	0.67867231

Vstm2l	947.0408772	1917.406877	0.493917534	6.87E-14	1.21E-10
Oaf	43.90512604	89.34574178	0.491407035	0.015015848	0.09176313
Phlda3	93.17272664	189.6494873	0.491289104	1.62E-06	8.08E-05
Nr2f6	359.12628	732.0851323	0.49055262	1.56E-05	0.000473244
Cmklr1	27.14052714	55.33611385	0.490466808	0.034695143	0.17046937
Hmcn2	45.35873635	92.63511669	0.489649476	0.389897269	0.819541498
Ninj1	78.15765154	159.6559327	0.489538035	0.000548977	0.007400449
H1fx	444.2894433	908.6903883	0.488933799	1.48E-06	7.60E-05
Fzd2	228.4594925	467.5672514	0.488613118	0.018602621	0.10760626
Tgfb1	61.45663448	125.8988414	0.488142971	0.004999183	0.040181371
Camk2n2	705.4638213	1446.944243	0.487554254	8.75E-14	1.45E-10
Prr7	411.9426207	845.3091498	0.487327767	3.29E-12	3.00E-09
Cebpb	125.0915692	256.9825742	0.486770629	6.28E-08	7.09E-06
Robo3	135.0528633	278.5844317	0.484782521	0.00224911	0.021893317
Snx20	16.60443914	34.47134814	0.481688128	0.014797233	0.090636043
Rras	56.28006735	116.9346684	0.481294967	0.00331943	0.029526333
Ppp2r3d	133.117118	276.9897347	0.480585023	2.34E-08	3.41E-06
Hic1	45.74894393	95.37631959	0.479667743	0.287956816	0.698898243
Ppp1r13l	76.03857676	158.6580434	0.479260775	2.69E-06	0.000120031
Zfpm1	187.2011411	391.0461504	0.478718793	9.31E-10	2.81E-07
Ier5l	477.559872	998.9209764	0.478075727	4.12E-09	9.50E-07
Ccdc85c	838.6250385	1757.902158	0.477060134	5.48E-07	3.64E-05
Reep6	142.99809	299.8826182	0.476846877	0.000736366	0.009310728
Samd11	429.4223767	901.1700654	0.476516468	5.34E-13	7.45E-10
Bbc3	675.6135864	1418.308863	0.476351523	1.81E-14	4.81E-11
Ppm1n	26.77948144	56.70047351	0.472297316	0.046164065	0.208745966
Insm1	1144.818999	2426.705044	0.47175861	5.57E-05	0.001243657
Samd1	736.6162227	1563.201765	0.471222742	8.87E-08	9.04E-06
Rho	87.81349927	186.9698691	0.469666582	0.001130596	0.012921929
Mcpt4	13.79785993	29.4228717	0.468950144	0.629422579	1
AI848285	111.4421371	237.8022624	0.468633628	4.77E-08	5.71E-06
Klhl35	95.7270305	204.4294203	0.468264452	0.006111877	0.046838879
Socs1	51.11726358	109.2217126	0.468013753	0.004967787	0.040013549
Metrn	75.34409687	161.1708991	0.467479534	0.004307006	0.03591339
Smoc2	31.14416655	66.63425005	0.467389766	0.565184014	0.975726398
Trim47	31.44100228	67.37060772	0.46668723	0.120358996	0.407435101
Col1a2	861.5572689	1848.03106	0.466202808	0.48236589	0.899340033
Prob1	126.7216594	272.4357833	0.465143227	8.40E-09	1.56E-06
Cngb1	110.4234829	238.4792805	0.463031768	2.39E-08	3.43E-06
Ly6a	10.30388224	22.4190468	0.45960394	0.382644544	0.810438972
Nid1	309.4694283	675.1393201	0.458378618	0.444177574	0.876560011
Prrt2	40.85134201	89.13665751	0.458300133	5.34E-05	0.001204791
Eln	518.3212875	1136.894693	0.455909673	0.386725441	0.815225329

Egr3	150.0097405	329.3120987	0.455524535	0.232484298	0.616085712
Mal	12.30044386	27.10136354	0.453868081	0.604221058	0.988955325
Cd248	86.99964748	192.2570986	0.452517218	0.305426504	0.719373168
Adamts2	48.82774156	107.9744207	0.452215824	0.450225299	0.876560011
Lgi4	81.4999542	180.5761117	0.451332978	0.118805948	0.403310526
Fbln2	275.2435958	611.0750346	0.450425202	0.20791232	0.575498999
Tfap2e	133.768232	296.9975586	0.45040179	0.002175032	0.02130519
Nxph4	234.0919864	520.4796031	0.449762075	3.43E-05	0.000867025
Aqp1	18.49424129	41.31232259	0.447668883	0.318770246	0.737461583
Thbs2	65.23795617	146.5320406	0.44521291	0.42140736	0.857973275
Ntn1	69.37022738	156.0791116	0.44445555	0.316401952	0.734779661
Lor	203.5642616	458.4365429	0.444040216	0.000104863	0.002026636
Kcnj8	16.59813292	37.39686585	0.443837539	0.299909228	0.712454492
Gpr27	368.1825495	830.7467042	0.443194716	5.08E-14	9.63E-11
C1ql2	219.1935588	495.5750392	0.442301451	2.76E-06	0.000122008
Igfbp6	28.04255437	63.79919552	0.439544012	0.452954213	0.876560011
Nme2	34.69441599	79.10945189	0.438562209	5.26E-05	0.001188154
Six2	37.83151681	86.36488153	0.438042826	4.64E-05	0.001090293
Col5a1	524.8055328	1206.118226	0.435119478	0.309682088	0.724632038
Acvrl1	23.04481327	53.00864629	0.434736876	0.16808492	0.505207406
Mir5125	28.33660403	65.24260813	0.434326659	0.000213568	0.003537139
Ptrf	83.59904163	192.5583263	0.434149191	0.436877677	0.874811092
Mgp	27.75556556	63.98849572	0.433758682	0.37161731	0.794918087
Mpz	108.0375057	249.3339112	0.4333045	0.637715003	1
Frat1	96.18542931	222.0032999	0.433261259	0.000656242	0.008496536
Arhgef19	138.8151635	320.4921781	0.433131206	0.012448264	0.07953691
Doc2a	188.3986007	435.4030789	0.432699284	8.33E-13	1.00E-09
Bcas1	10.2859706	23.91246378	0.430151017	0.020358977	0.115276242
Lfng	340.7970185	795.6036322	0.428350255	0.200802454	0.564153198
Anpep	24.01641449	56.15335958	0.427693279	0.45304594	0.876560011
Atoh7	64.78408452	152.8953523	0.423715198	0.23000628	0.612234901
Cdh5	38.12686385	90.2826255	0.422305661	0.397113674	0.828405805
Ltbp4	340.3464295	810.8453686	0.419742706	0.068613515	0.274495467
Pi16	216.0326127	519.7510243	0.415646343	0.370304742	0.793178244
Inhbb	250.8708946	605.9889546	0.413985919	0.024684548	0.133062712
Htra3	58.73079412	141.8816666	0.413942094	0.461509184	0.880893861
Cspg4	58.44701139	141.8163828	0.41213159	0.165223086	0.499949259
Pax7	14.49479128	35.26465789	0.411028836	0.004521874	0.037253864
Scarf2	73.47610224	179.8350657	0.408574946	0.084034374	0.317777448
Col5a3	89.33722633	219.1544752	0.407645001	0.428031632	0.862831592
Hspg2	532.5673067	1308.017656	0.407156053	0.223508013	0.602596694
Cdc42ep1	51.95183365	127.6998976	0.406827528	0.019772238	0.112704256
Dpt	23.04483383	56.91433092	0.404903887	0.429863661	0.865012131

Bri3	222.87623	553.1078552	0.402952567	1.29E-15	4.88E-12
Sox3	260.7123973	649.0726929	0.401669027	8.14E-09	1.53E-06
Eng	39.82809899	100.1063249	0.397857968	0.166748916	0.502901986
Col6a6	47.64400416	119.8693645	0.397466061	0.445837119	0.876560011
Col6a3	214.4324795	544.1381566	0.39407727	0.457552486	0.877510613
Bloc1s1	31.23238624	81.48630259	0.38328388	5.64E-06	0.000213126
Tie1	21.96923865	57.53476397	0.381842857	0.163284532	0.496462866
Vwf	22.02123563	58.34894544	0.377405889	0.249727296	0.642300583
Col1a1	911.8508132	2453.265071	0.371688663	0.379551318	0.805972832
C1ql1	126.4332878	341.5795285	0.370143048	4.17E-09	9.54E-07
Glcci1	151.6732237	427.4642611	0.354820829	4.85E-08	5.71E-06
Loxl1	77.5801949	221.3385944	0.350504597	0.306371932	0.720127484
Klf2	111.3654611	318.4360286	0.349726322	0.00327251	0.029246335
Pou3f3	64.709273	187.1780358	0.345709756	1.39E-06	7.23E-05
Igfbp7	71.60347766	212.6260546	0.336757778	0.168232303	0.505478497
Rhbdl1	17.69513932	54.53585639	0.324467983	1.25E-05	0.000395386
Foxd2	10.53747076	32.98106618	0.31950061	0.000590485	0.007852093
Dlk1	157.3526204	494.891424	0.317953823	0.306117268	0.719656485
Galnt2	19.77671456	64.77844489	0.305297767	8.13E-07	4.92E-05
Ltbp2	13.40518035	45.88989936	0.292116142	0.388163271	0.817127446
Nrtn	42.11793508	146.2063121	0.288071934	0.001723139	0.017813535
Tnxb	164.0813866	576.3850261	0.284673229	0.340596903	0.760848146
Adnp	193.394645	687.9141431	0.281131951	1.99E-29	5.27E-25
C1ql4	12.45255946	44.39869984	0.280471264	2.01E-05	0.00057688
Aldob	10.73280511	93.01349153	0.115389767	1.92E-11	1.10E-08
